# Turnerbactin, a Novel Triscatecholate Siderophore from the Shipworm Endosymbiont *Teredinibacter turnerae* T7901

**DOI:** 10.1371/journal.pone.0076151

**Published:** 2013-10-11

**Authors:** Andrew W. Han, Moriah Sandy, Brian Fishman, Amaro E. Trindade-Silva, Carlos A. G. Soares, Daniel L. Distel, Alison Butler, Margo G. Haygood

**Affiliations:** 1 Institute of Environmental Health, Oregon Health & Science University, Beaverton, Oregon, United States of America; 2 Department of Chemistry and Biochemistry, University of California Santa Barbara, Santa Barbara, California, United States of America; 3 Universidade Federal do Rio de Janeiro, Instituto de Biologia, Ilha do Fundão, CCS, Rio de Janeiro, Rio de Janeiro, Brasil; 4 Ocean Genome Legacy, Inc., Ipswich, Massachusetts, United States of America; University of New South Wales, Australia

## Abstract

Shipworms are marine bivalve mollusks (Family Teredinidae) that use wood for shelter and food. They harbor a group of closely related, yet phylogenetically distinct, bacterial endosymbionts in bacteriocytes located in the gills. This endosymbiotic community is believed to support the host's nutrition in multiple ways, through the production of cellulolytic enzymes and the fixation of nitrogen. The genome of the shipworm endosymbiont *Teredinibacter turnerae* T7901 was recently sequenced and in addition to the potential for cellulolytic enzymes and diazotrophy, the genome also revealed a rich potential for secondary metabolites. With nine distinct biosynthetic gene clusters, nearly 7% of the genome is dedicated to secondary metabolites. Bioinformatic analyses predict that one of the gene clusters is responsible for the production of a catecholate siderophore. Here we describe this gene cluster in detail and present the siderophore product from this cluster. Genes similar to the *entCEBA* genes of enterobactin biosynthesis involved in the production and activation of dihydroxybenzoic acid (DHB) are present in this cluster, as well as a two-module non-ribosomal peptide synthetase (NRPS). A novel triscatecholate siderophore, turnerbactin, was isolated from the supernatant of iron-limited *T. turnerae* T7901 cultures. Turnerbactin is a trimer of *N*-(2,3-DHB)-L-Orn-L-Ser with the three monomeric units linked by Ser ester linkages. A monomer, dimer, dehydrated dimer, and dehydrated trimer of 2,3-DHB-L-Orn-L-Ser were also found in the supernatant. A link between the gene cluster and siderophore product was made by constructing a NRPS mutant, TtAH03. Siderophores could not be detected in cultures of TtAH03 by HPLC analysis and Fe-binding activity of culture supernatant was significantly reduced. Regulation of the pathway by iron is supported by identification of putative Fur box sequences and observation of increased Fe-binding activity under iron restriction. Evidence of a turnerbactin fragment was found in shipworm extracts, suggesting the production of turnerbactin in the symbiosis.

## Introduction

Iron is required by all but a few organisms and serves as an important cofactor for many essential enzymes. The insolubility of Fe(III) at physiological pH and aerobic conditions severely limits its bioavailability. In the world's oceans, 99% of the Fe(III) is bound by uncharacterized organic ligands [1], [2]. Similarly, iron is also a limiting nutrient for bacteria in animals, where it is often tightly bound to proteins such as ferritin, transferrin, lactoferrin, or incorporated into heme containing proteins [3], [4]. The ability of a bacterium to acquire iron may be critical to the successful colonization of its host. In response to iron limitation, many bacteria and some fungi produce siderophores, low-molecular weight compounds with a high affinity for Fe(III). Hundreds of siderophores have been characterized, the majority being from terrestrial organisms [5], [6], [7]. Siderophores can be classified by their mode of biosynthesis, non-ribosomal peptide synthetase (NRPS)-dependent or NRPS-independent biosynthesis [8], as well as by their major type of functional binding groups, catechols, hydroxamic acids, and α-hydroxycarboxylic acids.

The class of siderophores that possess the highest stability constants measured to date are the triscatecholate siderophores, including enterobactin [9], [10], a cyclic trimeric ester of 2,3-dihydroxybenzoate (DHB)-L-Ser (K_f_ = 10^49^, [11]) and bacillibactin [12], a cyclic trimeric ester of 2,3-DHB-Gly-L-Thr (K_f_ = 10^47.6^, [13]). Other examples of cyclic triscatecholate siderophores include salmochelin, a glucosylated derivative of enterobactin [14], cyclic trichrysobactin [15], and streptobactin [16]. The recently reported cyclic trichrysobactin is a trimeric ester of the previously reported chrysobactin, 2,3-DHB-D-Lys-L-Ser [17], and was isolated from the plant pathogen *Dickeya chrysanthemi*. Streptobactin, a cyclic trimeric ester of 2,3-DHB-L-Arg-L-Thr, was recently reported from the marine-derived actinomycete *Streptomyces* sp. YM5-799. In addition to the cyclic triscatecholate siderophores, a linear (non-cyclized) triscatecholate siderophore, trivanchrobactin, was reported from the marine isolate *Vibrio campbelli* DS40M4 [18], [19]. Trivanchrobactin is a linear trimeric ester of the previously reported vanchrobactin, 2,3-DHB-D-Arg-L-Ser [20].

The shipworm endosymbiont *Teredinibacter turnerae* has been found in numerous genera and species of teredinid bivalves from across the globe [21]. Shipworm endosymbionts have been shown to supplement the nitrogen-poor wood diet by providing a source of fixed nitrogen and are thought to produce cellulolytic enzymes that may assist the shipworm host in the degradation of wood [21]. The genome of *T. turnerae* T7901 was recently sequenced [22]. Although *T. turnerae* is an intracellular symbiont, its genome does not show the typical modifications experienced by obligate endosymbionts, most notably reduced genome size, higher A+T content, and loss of DNA repair and transcriptional regulatory genes [23]. Its genome size is 5.1 Mb, with a 50.8% G+C content, and includes genes involved in almost all core metabolic functions, including DNA repair [22]. These observations suggest either *T. turnerae* is a facultative symbiont, or is part of a recently established symbiosis. To date, *T. turnerae* has not been detected outside of the shipworm host. Analysis of *T. turnerae*'s genome also reveals nine gene clusters predicted to code for secondary metabolites, constituting nearly 7% of its genome. One of the nine gene clusters was recently reported to synthesize an antibacterial compound in the tartrolon family [24].

Here we report on another secondary metabolite gene cluster from *T. turnerae* T7901, a cluster that was previously proposed to encode a siderophore biosynthesis gene cluster [22]. This work presents the structural characterization of a novel triscatecholate siderophore, *N*-(2,3-DHB)-L-Orn-L-Ser, named turnerbactin. The gene cluster responsible for the biosynthesis of turnerbactin is analyzed and a model for its biosynthesis is proposed. The biosynthetic genes involved in turnerbactin production are herein referred to as *tnb*, for *t*ur*n*erbactin *b*iosynthesis. Turnerbactin represents the first described siderophore from a shipworm symbiont.

## Methods

### Strain information

*Teredinibacter turnerae* T7901 was originally isolated from the shipworm *Bankia gouldi* collected in the wild near Duke University Marine Lab, Beaufort, NC [21].

### Culture conditions

*T. turnerae* T7901 was grown in a low-iron, modified version of shipworm basal medium (SBM) [21], [25] containing 750 ml artificial seawater [26], 250 ml distilled water, 0.1 mM KH_2_PO_4_, 0.094 mM Na_2_CO_3_, 0.01 mM Na_2_MoO_4_•2H_2_O, 0.5% (w/v) sucrose, 5 mM NH_4_Cl, 20 mM HEPES buffer (pH 8.0), 0.1 μM EDTA-chelated ferric iron (Sigma), and 1 ml A5+Co trace metal mix [27]. Cultures were grown in 2.8 L Fernbach flasks with 2 L culture medium at 30°C on an orbital shaker (110 rpm). A pre-culture of *T. turnerae* was prepared by inoculating an iron-replete (10 μM EDTA-chelated ferric iron) SBM liquid culture with a single colony from an iron-replete SBM agar plate. The 2 L cultures were inoculated with 1 mL of overnight-grown pre-culture. Cultures were grown for 2 days, when Fe(III)-binding activity of culture supernatants reached maximum activity as measured by the chrome azurol sulfonate (CAS) assay [28].

To determine CAS activity of iron-replete and iron-limited cultures, duplicate 50 mL SBM cultures of *T. turnerae* T7901 were grown in 100 mL flasks with the same conditions described above, containing either 10 μM EDTA-chelated ferric iron (iron-replete) or 0.1 μM EDTA-chelated ferric iron (iron-limited). After growth for approximately 38 hours at which point cultures were at late log phase, 1 mL aliquots of all cultures were collected and centrifuged at 10000 rpm for 3 min. Supernatants were tested for CAS activity. Uninoculated iron-replete media showed no CAS activity, indicating that the EDTA from the iron source did not interfere with the assay.

All growth and CAS activity measurements were recorded on a SpectraMax M2 Multidetection Reader (Molecular Devices) with absorbance measured at 600 nm and 630 nm, respectively. CAS activity was calculated as (1-(Ab_sample_/Ab_blank_) ×100.

### Compound isolation

Culture supernatant was cleared of cells and debris by centrifugation at 10000 rpm for 25 min. Decanted supernatant was incubated with ∼20 g/L Dianion HP20 resin in 2.8 L Fernbach flasks at 4°C on an orbital shaker (110 rpm) for 4 hrs. The resin was collected and washed sequentially with MilliQ water followed by 25%, 50%, and finally 100% isopropanol (IPA). The siderophores eluted with the 25% IPA fraction as detected by the CAS assay. The 25% IPA fraction was concentrated by rotary evaporation *in vacuo*. The siderophores were purified by reverse-phase high–performance liquid chromatography (RP-HPLC) on a semi-preparative C_18_ column (10 mm internal diameter ×25 cm length, 5 μm particle size, Ascentis) using water and acetonitrile (ACN) as solvents at a flow-rate of 3 mL/min at 25°C. Both solvents contained 0.05% trifluoroacetic acid (TFA). The mobile phases consisted of a gradient of 10% ACN in water to 15% ACN from 0–10 min, followed by an isocratic step of 15% ACN from 10–15 min, then a gradient from 15% to 25% ACN from 15–35 min, then a final gradient from 25% to 100% ACN from 35–40 min. The eluent was continuously monitored at 215 nm and 320 nm. Fractions were collected manually and immediately tested for CAS activity and then concentrated under vacuum. Collected and pooled fractions were further purified on an analytical RP-Amide-C_16_ column (4.6 mm internal diameter ×25 cm length, 5 μm particle size, Ascentis) using the same method as above at a flow-rate of 1 mL/min. Pure siderophores were lyophilized. The approximate yields of siderophores from a 4 L culture are 1.2 mg DHB-Orn-Ser, 2.1 mg (DHB-Orn-Ser)_2_, 2.7 mg turnerbactin, 2.4 mg dehydro-(DHB-Orn-Ser)_2_, and 1.2 mg dehydro-(DHB-Orn-Ser)_3_.

The triscatecholate siderophores that have been reported in the literature have not contained dehydrated amino acid constituents. Additionally, with the exception of linear trivanchrobactin, the triscatecholate siderophores have been found in the cyclic form. A control experiment was carried out to rule out the possibility that the dehydrated siderophores and the lack of a cyclic trimer were the result of the purification method. A side-by-side purification of the structurally similar siderophore cyclic trichrysobactin [15] was carried out with siderophores from *T. turnerae* T7901. Cultures of *Dickeya chrysanthemi* were grown and siderophores were purified as described by Sandy and Butler [15]. Siderophores from culture extracts of *T. turnerae* T7901 were purified concurrently and in the same manner. The identity of siderophores from both cultures was confirmed by HPLC retention time, high-resolution electrospray ionization mass spectrometry (HRESIMS), and ^1^H-NMR.

Mass spectra were obtained on a ThermoElectron LTQ-Orbitrap high-resolution mass spectrometer. Samples were dissolved in 50% methanol and MS analysis was performed in positive mode using electrospray ionization (ESI). All 1-D and 2-D NMR experiments were carried out on a Bruker Avance II Ultrashield Plus 800 MHz instrument with a cryoprobe in *d*_4_-methanol (CD_3_OD, Cambridge Isotope Laboratories).

UV-visible absorption spectrum of purified turnerbactin in water was collected on a Varian Cary 50 spectrophometer.

### Amino acid analysis

A dried preparation of purified turnerbactin (∼1 mg) was hydrolyzed in 200 μL 6 M HCl for 17 hours at 110°C. The solution was brought to room temperature and evaporated to dryness. The dried, hydrolyzed sample was redissolved in 100 μL H_2_O, to which 200 μL of a 1% (w:v) solution of Marfey's reagent (1-fluoro-2–4-dinitrophenyl-5-L-alanine amide [FDAA]) [29] in acetone and 40 μL 1 M NaHCO_3_ were added to derivatize the primary amines of the amino acids. The reaction was heated for 1 hour at 40°C, after which 20 μL 2 M HCl was added to stop the reaction. The derivatized sample was analyzed by HPLC on an analytical YMC ODS-AQ C_18_ column (4.6 mm, i.d. ×250 mm L, Waters Corp.) using a linear gradient from 90% water with 0.1% TFA/10% CH_3_CN to 60% water with 0.1% TFA/40% CH_3_CN over 45 min. The eluent was continuously monitored on a Waters UV-visible detector at 340 nm. The derivatized sample was compared to chiral amino acid standards prepared the same way. Peaks were collected and verified by MS.

### Chemical extraction of shipworms

A laboratory culture of *Lyrodus pedicellatus* maintained at the Ocean Genome Legacy was sent to Oregon Health & Science University and stored at room temperature in an aerated aquarium until further processed. *L. pedicellatus* specimens were removed from wood using pliers, slowly peeling away layers of wood until shipworms were exposed, careful to not damage the shipworms. Six *L. pedicellatus* shipworms were removed from the wood, rinsed in filter-seawater, pooled, and lyophilized. The resulting dry weight was 85.4 mg. The tissue was then homogenized with a plastic pestle. The homogenized tissue was extracted three times with three volumes of methanol on a rotary mixer for one hour. The methanolic extract was separated from the tissue by centrifugation at 15,000 rpm for 3 min and subsequently dried. Approximately 1 mg of the crude methanolic extract was dissolved in water and injected onto a ThermoElectron LTQ-Orbitrap high-resolution mass spectrometer with an Accela HPLC system using a C_18_ column (2.1 mm internal diameter ×10 cm length, 3 μm particle size, Ascentis). Mass spectra were recorded in positive mode using ESI. The following gradient was used at a flow rate of 200 ul/min: 10% methanol in water to 50% methanol over 30 min, followed by an increase to 100% methanol over 5 min. Both solvents contained 0.1% formic acid. Pure siderophores (approximately 10 μg) were used as standards using the same method. The *L. pedicellatus* extract was analyzed first, followed by washing of the column, a blank injection, and then the pure siderophore standard.

### Bioinformatic analysis of NRPS domains

Module identification and domain organization of the NRPS was carried out using the online tools NRPS-PKS [30] and the PKS/NRPS analysis website [31]. Additional analyses utilized the software package HMMER [32] available from the website http://hmmer.org/. Alignments for Profile Hidden Markov Models (pHMMs) were downloaded from either the Pfam database [33] or the files supplied by Rausch et al. [34]. The hmmbuild command in HMMER was then used to build pHMMs.

Adenylation (A) domain: Protein sequences of A domains in TnbF were retrieved using the PKS/NRPS analysis website [31]. The specificity-conferring code of TnbF A domains were determined using the NRPSpredictor2 tool [35].

Condensation (C) domain: Protein sequences of C domains in TnbF were retrieved using the PKS/NRPS analysis website [31]. Aligned reference protein sequences of C domains were downloaded from Rausch et al. [34] and a subset of 91 sequences was used for the current study. The TnbF C domains were added to the downloaded C domain alignment using the multiple sequence alignment program MUSCLE [36]. The alignment was edited in Geneious v5.4 [37]. A maximum likelihood (ML) tree of C domains was reconstructed using RAxML 7.2.7 [38], implemented through the CIPRES portal [39]. The amino acid substitution matrix used in this analysis was the JTT matrix [40], with the Γ model of rate heterogeneity. RAxML's rapid bootstrap was performed with 100 replicates and the best scoring ML tree was saved.

### Construction of *tnbF* plasmid insertion disruption mutant

For construction of the plasmids used for gene disruption, a region targeting the first C domain within *tnbF* was amplified with specific PCR primers TnbF851F and TnbF1193R (Table 1) and the high-fidelity Phusion DNA polymerase (Finnzymes). The addition of 3′ A-overhangs for TA cloning was carried out by DyNAzyme II DNA polymerase (Finnzymes) following the manufacturer's protocol. The amplicon was purified using the QIAquick PCR Purification Kit (Qiagen) and then cloned into pCR2.1 (Invitrogen). Plasmid DNA was isolated and double digested with XhoI and SacI. The resulting fragment was gel purified with the GENECLEAN II Kit (MP Biomedicals) and then ligated with the Quick Ligation Kit (NEB) into suicide vector pDM4, which had been double digested with the same restriction enzymes, resulting in plasmid pDMtnbF. The plasmid pDMtnbF was transformed into *E. coli* S17–1 λpir. This plasmid was then conjugated into *T. turnerae* T7901, and plasmid cointegrates were selected on SBM plates with 0.5% (w/v) Sigmacell cellulose Type 101 (Sigma) as the sole carbon source and 10 μg/ml chlorampenicol (Cm). The location of integration by pDMtnbF into the chromosome of T7901 was confirmed by PCR and DNA sequencing with vector-specific forward primers pNQ705 and pDM4CAT189 and the T7901 chromosomal-specific reverse primer TnbF1604R (Table 1). A list of all strains and plasmids used in this study are provided in Table 2.

**Table 1:** List of primers used in this study.

Primer	Sequence (5′–3′)	Reference
TnbF851F	AAACCCTGGGAATGCCGTTTATGC	This study
TnbF1193R	TGCACGCCAAATTCAAAGTCGTCC	This study
TnbF1604R	TTTGCATAATGGCGAACATCGCGG	This study
pNQ705	TTTGCGTAACGGCAAAAGCAC	Modified from Rock and Nelson, 2006 [64]
pDM4CAT189	GAGCATTCATCAGGCGGGCA	This study

**Table 2:** List of strains and plasmids used in this study.

Strain or plasmid	Description	Reference
*T. turnerae* strains		
T7901	Wild-type	Distel et al., 2002 [21]
TtAH03	*tnbF*::Cm^r^	This study
*E. coli* strains		
TOP10	F- *mcr*A Δ(*mrr*-*hsd*RMS-*mcr*BC) Φ80*lac*ZΔM15 Δ*lac*X74 *rec*A1 *ara*D139 Δ(*ara-leu*) 7697 *gal*U *gal*K *rps*L (Str^r^) *end*A1 *nup*G	Invitrogen
S17–1 λpir	*thi pro hsdR hsdM^+^ recA* RP4–2-Tc::Mu-Km::Tn7λpir	Simon et al., 1983 [65]
Plasmids		
pCR2.1	T-vector, Km^r^, Amp^r^	Invitrogen
pDM4	Suicide vector, *sacB* gene, R6K origin, Cm^r^	Milton et al., 1996 [66]
pDMtnbF	Portion of tnbF gene cloned into pDM4	This study

Crude extracts from TtAH03 were analyzed and compared to those from wild-type strain T7901. Each strain was grown in iron-limited SBM medium with 0.5% (w/v) Sigmacell cellulose Type 101, with 10 μg/ml Cm in the case of TtAH03. Spent supernatant was extracted with HP20 resin. HP20 resin was washed with MilliQ water, 50% IPA, and 100% IPA. The 50% IPA fraction from each culture was dried by lyophilization and approximately 0.1 mg of each extract was used for HPLC analysis using the same method as described for compound isolation. Pure DHB was purchased from Sigma.

## Results

### Biosynthetic gene cluster

**Figure 1:**
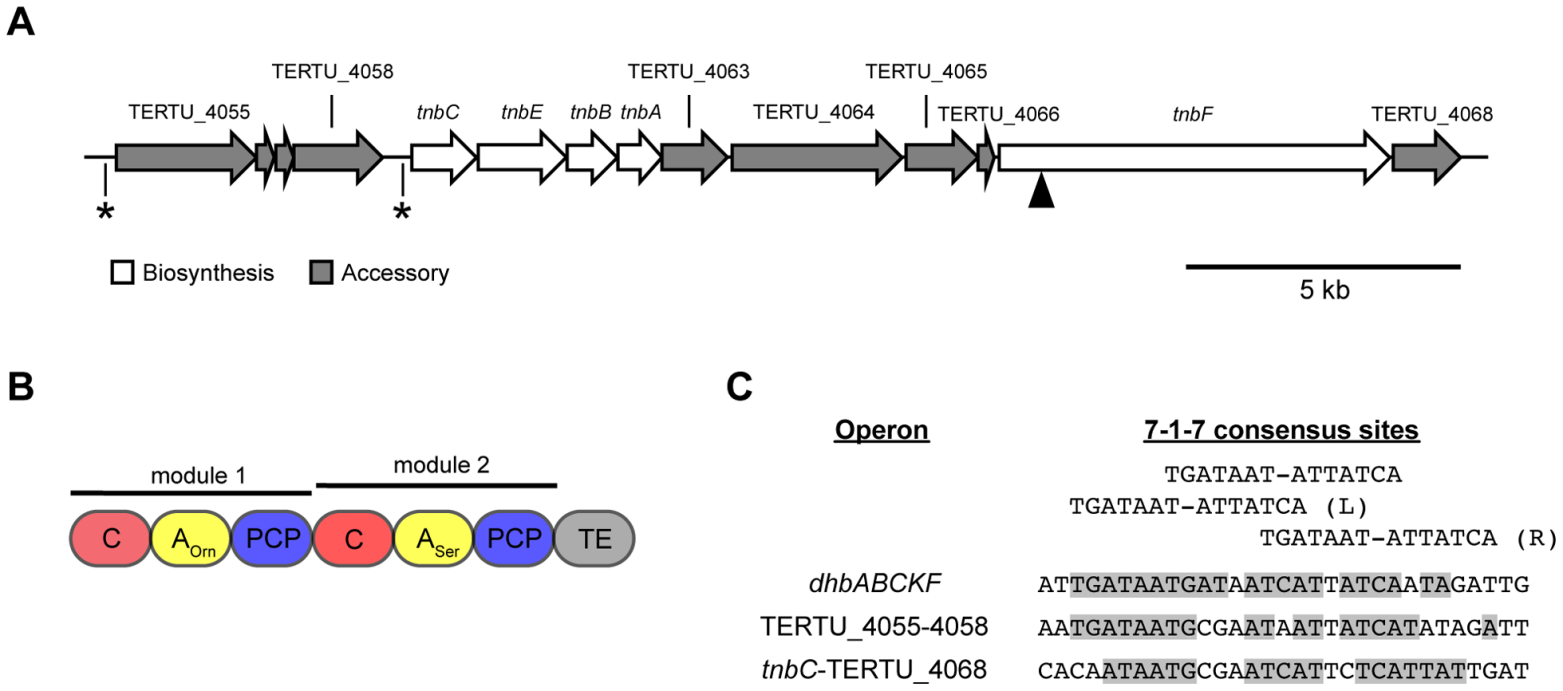
Overview of the turnerbactin biosynthetic gene cluster. A. Organization of genes involved in turnerbactin biosynthesis. The locations of putative Fur boxes are indicated by asterisks (*). The black triangle indicates the location of NRPS disruption. B. Domain organization of the NRPS modules of TnbF. C, condensation domain; A, adenylation domain; PCP, peptidyl-carrier protein; TE, thioesterase. C. Putative Fur box sequences of turnerbactin's biosynthetic gene cluster compared to the bacillibactin biosynthetic operon of *Bacillus subtilis*. Shaded bases match the revised view of Fur box sequences consisting of two overlapping 7–1–7 motifs proposed by Baichoo and Helmann.

Annotation of the genome of *Teredinibacter turnerae* T7901 revealed a gene cluster with similarity to siderophore biosynthesis and iron transport genes [22] (Figure 1A). Proteins with closest similarity to the gene products of this cluster are shown in Table 3 and suggest the production of a catecholate siderophore. The genes *tnbCEBA* are homologous to the *entCEBA* genes, responsible for the biosynthesis and activation of 2,3-dihydroxybenzoate (DHB) via the shikimate pathway [41]. EntC isomerizes chorismate into isochorismate, then the N-terminal portion of EntB hydrolyzes isochorismate into 2,3-dihydro-DHB, which is oxidized to DHB by EntA. EntE then activates and transfers DHB to the aryl carrier C-terminal portion of EntB.

**Table 3:** Proteins with similarity to the products of the turnerbactin biosynthetic gene cluster. S: similarity, I: identity.

Gene	Protein size	Proposed function	Homolog (Accession, organism)	S/I (%)
TERTU_4055	648	TonB-dependent receptor	CCD03052, *Azospirillum brasilense* Sp245	68/48
TERTU_4056	87	Hypothetical protein	ABL99859, *Shewanella amazonensis*	51/37
TERTU_4057	100	Hypothetical protein	EGM6880, *Shewanella* sp. HN-41	72/56
TERTU_4058	550	PepSY-associated TM helix domain protein	ABK46690, *Shewanella* sp. ANA-3	64/48
*tnbC*	394	Isochorismate synthase	AAN33228 *Brucella suis* 1330	66/51
*tnbE*	538	2,3-dihydroxybenzoate-AMP ligase	EHK70948 *Pseudomonas psychrotolerans* L19	76/61
*tnbB*	292	Isochorismatase	CAG23680 *Photobacterium profundum* SS9	73/54
*tnbA*	255	2,3-dihydroxybenzoate-2,3-dehydrogenase	EHK70946 *Pseudomonas psychrotolerans* L19	72/58
TERTU_4063	389	RND family efflux transporter	ABE56876 *Shewanella denitrificans* OS217	66/44
TERTU_4064	1047	HAE1 family RND transporter	ABC30520 *Hahella chejuensis* KCTC 2396	76/60
TERTU_4065	445	Esterase	ACS85292 *Dickeya dadantii* Ech703	50/38
TERTU_4066	85	MbtH domain protein	ACZ77654 *Dickeya dadantii* Ech586	73/56
*tnbF*	2399	NRPS	AAN28936 *Acinetobacter baumannii*	62/45
TERTU_4068	417	Enterobactin exporter	ZP_10160264 *Vibrio campbellii* DS40M4	73/52

The gene *tnbF* codes for a 2399 amino acid, two module NRPS. Each module contains a C, A, and peptidyl-carrier protein (PCP) domain, followed by a C-terminus thioesterase (TE) domain (Figure 1B). A phosphopantetheinyl (P-pant) transferase, which is required for NRPS biosynthesis [42], was not found at this locus. The genome of *T. turnerae* T7901 codes for two P-pant transferases, TERTU_1510 and TERTU_4652. Due to its homology to the *entD* gene of enterobactin biosynthesis, the gene product of TERTU_1510 is proposed to be the P-pant transferase that posttranslationally attaches a P-pant moiety to a conserved Ser residue of the apo-PCP domains of TnbF.

In addition to biosynthesis genes, a number of other genes are also found in this cluster that are likely to assist in siderophore transport and uptake. TERTU_4066 codes for an MbtH-like protein [43], a protein of unknown function that is found in many but not all NRPS gene clusters. TERTU_4068 codes for a putative homologue of EntS, a 12 trans-membrane domain-containing efflux pump belonging to the Major Facilitator Superfamily (MFS) shown to excrete enterobactin in *Escherichia coli* [44]. TERTU_4055 codes for a putative TonB-dependent receptor, required for the recognition and uptake of the ferric siderophore complex. TERTU_4065 shares similarity to the gene encoding the enterobactin esterase, *fes*. In *E. coli*, once ferric enterobactin has entered the cell, iron is removed from the siderophore by hydrolyzing the ester bonds of the siderophore's backbone with the *fes* gene product [45].

**Figure 2:**
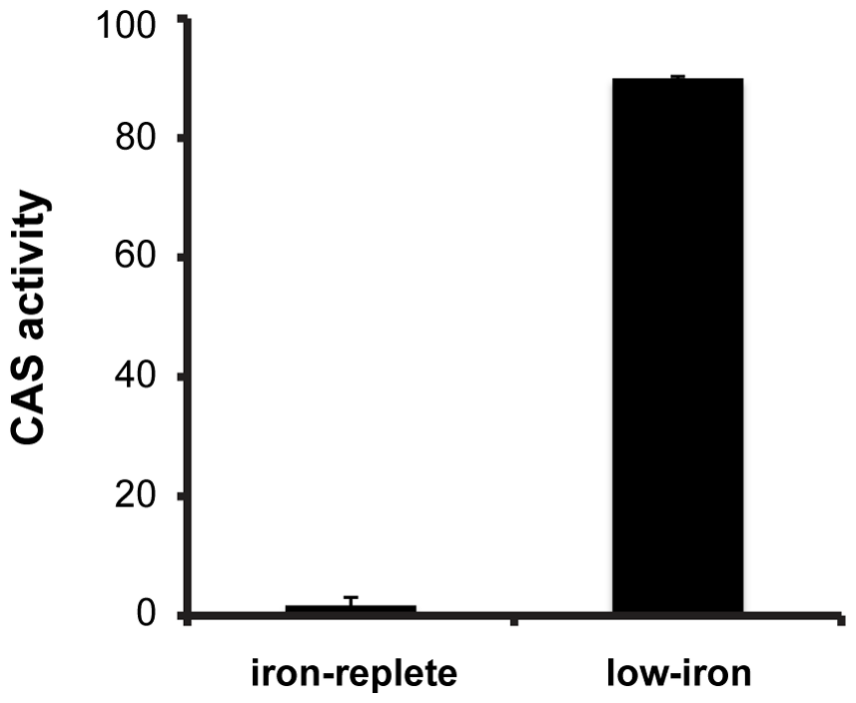
Fe(III)-binding activity of *T. turnerae* T7901 cultures. The CAS assay was used to measure Fe(III)-binding activity of iron-replete and iron-limited culture supernatants. Iron-replete conditions contained 10 μM ferric iron, while iron-limited conditions contained 0.1 μM ferric iron.

This gene cluster also shows two putative ferric uptake regulator (Fur) box sequences (Figure 1C), suggesting transcriptional regulation by one of the three Fur homologs found in the genome, TERTU_0053, TERTU_3299, and TERTU_3389. These sequences are compared to the bacillibactin biosynthetic operon of *Bacillus subtilis* in Figure 1C, whose Fur box matches exactly to the classic Fur box sequence proposed by de Lorenzo et al. [46]. The Fur box sequences of turnerbactin's biosynthetic gene cluster show high similarity with the two overlapping 7–1–7 motifs proposed by Baichoo and Helmann [47]. This similarity suggests that the turnerbactin biosynthetic gene cluster is composed of two iron-regulated operons: the first operon consisting of the genes TERTU_4055–4058, and the second operon consisting of the genes *tnbC*-TERTU_4068. Support for the role of iron in regulating expression of this gene cluster was provided by assaying CAS activity of iron-limited and iron-replete cultures (Figure 2). The high CAS activity observed in iron-limited cultures in conjunction with the negligible activity observed in iron-replete conditions suggests that low iron conditions leads to increased production of the siderophore.

### NRPS domain specificities

The crystal structure of the Phe-activating A domain of gramicidin synthetase A (GrsA) by Conti et al. [48] identified 10 residue positions critical in substrate binding. Stachelhaus et al. [49] and Challis et al. [50] determined that these 10 residues correspond to similar residues in other A domains and that these residues would form a “specificity-conferring code” that could be used to infer specificity of uncharacterized A domains. The amino acid specificity of A domains involved in turnerbactin biosynthesis was analyzed by comparing their respective active-site residues to those of known A domains.

The online tool NRPSpredictor2 [35] was used to determine specificity-conferring codes. The specificity-conferring code of TnbE, believed to be involved in 2,3-DHB biosynthesis and activation, is PLPAQGVVNK, which matches exactly that of DhbE, the homologue in bacillibactin biosynthesis. The specificity-conferring code of the second module (M2) A domain was determined to be DVWHFSLVDK, matching exactly with that of the Ser-activating A domain of the enterobactin synthetase as well as several other characterized Ser-activating A domains. However, the prediction for the first module (M1) A domain was inconclusive, as the specificity-conferring code of DSDDGGLVDK did not match any previously characterized specificity-conferring codes. The closest matches to known specificity-conferring codes at 60% were that of the M1 A domain of the chrysobactin synthetase (CbsF) and the M1 A domain of the vanchrobactin synthetase (VabF) which activate Lys and Arg, respectively. BLAST analysis of the entire M1 A domain showed highest similarity to the M1 A domains of CbsF and VabF at 65%/44% (similarity/identity) and 59%/42%, respectively.

In addition to A domain specificity prediction, C domains can also be subject to bioinformatic prediction. Rausch et al. [34] demonstrated that the reconstructed phylogeny of C domains shows a grouping according to function rather than species phylogeny or substrate specificity. The results of a maximum likelihood phylogenetic analysis with TnbF's C domains show that the TnbF M1 C domain groups with other Starter domains, as expected from its location in the NRPS (Figure S1). The M2 C domain of TnbF groups within the ^D^C_L_ functional domains and this placement is strongly supported, evidenced by the high bootstrap values at the base of the Dual E/C and ^D^C_L_ groups. In most cases, ^D^C_L_ domains are preceded by an epimerase (E) domain, catalyzing the epimerization of amino acids from L to D configuration. However, an E domain was not detected in TnbF through pHMM analysis and an external racemase was not detected in the gene cluster. In conjunction with the chemical analysis of the siderophore product (below), this suggests that the M2 C domain of TnbF exhibits ^L^C_L_ activity as opposed to its predicted ^D^C_L_ activity.

### Chemical characterization of the siderophore

**Figure 3:**
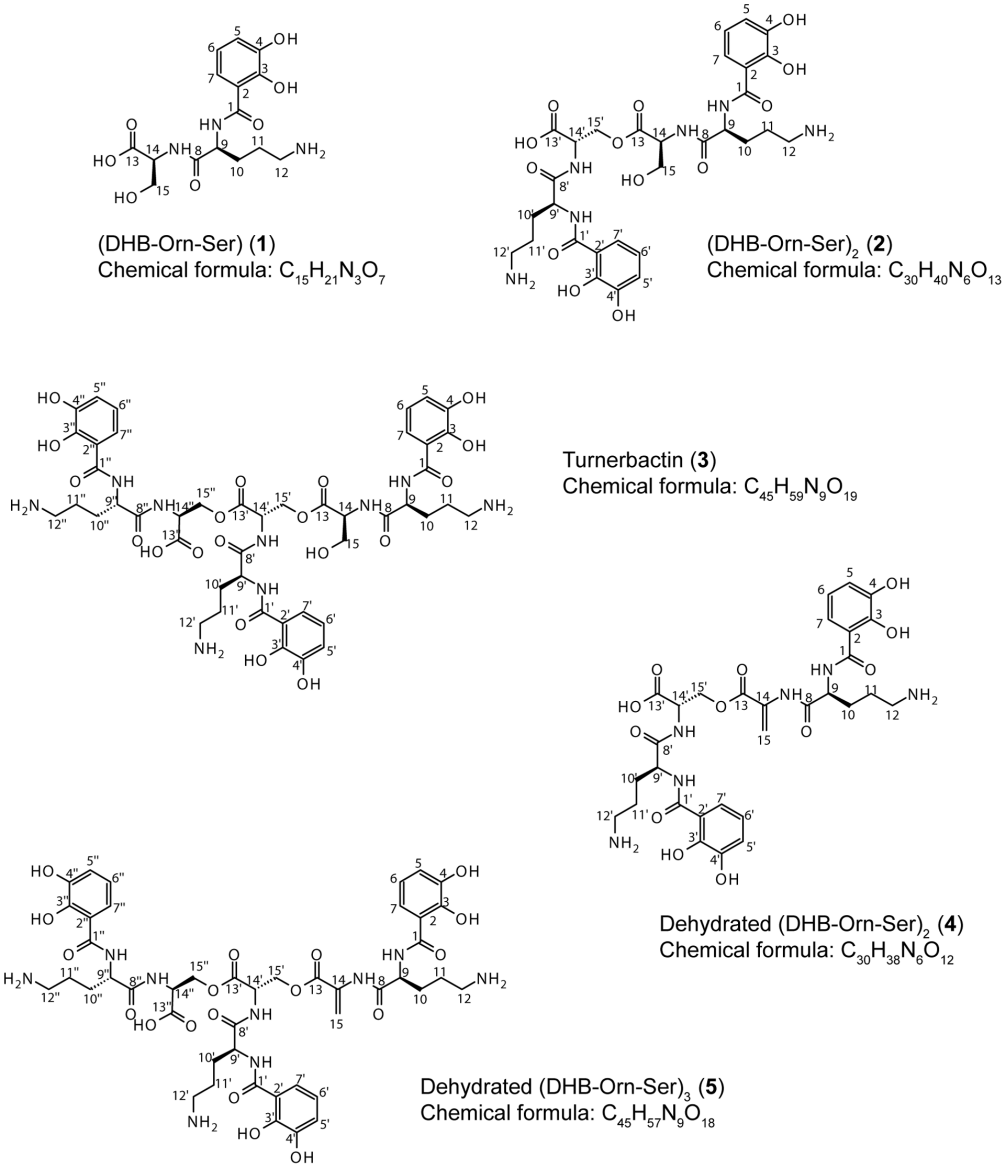
Structures of siderophores isolated from *T. turnerae* T7901.

The structures of the siderophores isolated from *T. turnerae* T7901 are shown in Figure 3. The siderophores from *T. turnerae* were purified from iron-deficient liquid SBM cultures by adsorbing siderophores from culture supernatants on an HP20 column, followed by purification by RP-HPLC. The CAS assay was used to track the siderophores throughout the purification process. RP-HPLC revealed five peaks displaying CAS activity (Figure S2). High resolution electrospray ionization mass spectrometry (HRESIMS) determined the mass of the molecular ion [M+H]^+^: DHB-Orn-Ser (**1**), m/z 356.1454, corresponding to a molecular formula of C_15_H_22_N_3_O_7_ (calculated 356.1452); (DHB-Orn-Ser)_2_ (**2**), m/z 693.2736, corresponding to a molecular formula of C_30_H_41_N_6_O_13_ (calculated 693.2726); turnerbactin (**3**), m/z 1030.4003, corresponding to a molecular formula of C_45_H_60_N_9_O_19_ (calculated 1030.4000); dehydro-(DHB-Orn-Ser)_2_ (**4**), m/z 675.2626, corresponding to a molecular formula of C_30_H_39_N_6_O_12_ (calculated 675.2620); dehydro-(DHB-Orn-Ser)_3_ (**5**), m/z 1012.3905, corresponding to a molecular formula of C_45_H_58_N_9_O_18_ (calculated 1012.3894).

ESIMS/MS analysis of each of these compounds is summarized in Table 4. All compounds exhibited similar fragmentation patterns, with overlap of fragments when applicable. The masses of the fragments could be correlated with the loss of various constituents of the siderophore (Table 4).

**Table 4:** Molecular ions and common mass fragments of siderophore from *T. turnerae* T7901. Fragment losses refer to the compound listed immediately above in the table.

Dehydrated (DHB-Orn-Ser)_3_ (5)	Dehydrated (DHB-Orn-Ser)_2_ (4)	Turnerbactin (3)	(DHB-Orn-Ser)_2_ (2)	(DHB-Orn-Ser) (1)	Fragment
1012.4	675.2	1030.4	693.3	356.1	Parent ion
762.3		780.3			Loss of DHB-Orn
675.2		693.3	675.2		Loss of Ser
425.2	425.2	443.2	443.2		Loss of DHB-Orn
338.1	338.1	356.1	356.1		Loss of Ser
251.1	251.1	251.1	251.1	251.1	DHB-Orn
115.1	115.1	115.1	115.1	115.1	Orn

The 1–D ^1^H and ^13^C NMR assignments of **1** were confirmed by 2–D ^1^H-^1^H TOCSY, HSQC, and HMBC experiments (Table 5, Figures S4-S14). The ^13^C NMR spectrum shows 15 distinct C resonances corresponding to three carbonyl carbons (δ 169.98 to 172.12), four methylene carbons (δ 23.39 to 38.93 for ornithine, δ 61.35 for serine), two methine carbons (δ 52.26 for ornithine, δ54.82 for serine), and six aromatic carbons (δ 116.01 to 147.81). The ^1^H NMR spectrum shows 12 distinct resonances corresponding to three aromatic protons (δ 6.77 to 7.36), seven methylene protons (δ 1.79 to 3.00 ornithine, δ 3.88, 3.98 for serine), and two methine protons (δ 4.82 for ornithine, δ 4.55 for serine). The aromatic splitting pattern in the ^1^H NMR spectrum is indicative of a 2,3-DHB moiety. A HMBC correlation between the α-proton of ornithine and the carbonyl carbon of DHB indicates that DHB is attached to the α-amine group of ornithine. A HMBC correlation between the α-proton of serine and the carbonyl carbon of ornithine confirms the ornithine-serine peptide bond.

**Table 5:** NMR data for 1 and 3 (800 MHz) in CD_3_OD.

	(DHB-Orn-Ser) (1)	Turnerbactin (3)					
Position	δ_C_	δ_H_ (*J* in Hz)	TOCSY	HMBC	δ_H_ (*J* in Hz)	HSQC	HMBC
DHB							
1, 1′, 1′′	169.98						169.1
2, 2′, 2′′	116.01						115.9
3, 3′, 3′′	147.81						147.7
4, 4′, 4′′	145.82						145.7
5, 5′, 5′′	118.38	6.98, dd (1.6, 8.0), [1H]	6, 7	2, 3, 4, 6, 7	6.97, m, [3H]	118.5	2, 3, 4, 6, 7
6, 6′, 6′′	118.52	6.77, t (8.0), [1H]	5, 7	1, 2, 3, 4, 5, 7	6.76, m, [3H]	118.7	1, 2, 3, 4, 5, 7
7, 7′, 7′′	118.44	7.36, dd (0.8, 8.0), [1H]	5, 6	1, 2, 3, 4, 5, 6	7.36, m, [3H]	118.6	1, 2, 3, 4, 5, 6
Ornithine							
8, 8′, 8′′	172.12						172.2
9	52.26	4.82, dd (5.6, 8.0), [1H]	10, 11, 12	1, 8, 10, 11	4.71, m, [1H]	52.6	1, 8, 10, 11
9′					4.77, m, [1H]	52.5	1′, 8′, 10′, 11′
9′′					4.79, m, [1H]	52.2	1′′, 8′′, 10′′, 11′′
10, 10′, 10′′	29.06	2.09, m [1H]; 1.89, m [1H]	9, 10, 11, 12; 9, 10, 11, 12	8, 9, 11, 12	2.05, m, [3H]; 1.89, m, [3H]	28.7	8, 9, 11, 12; 8, 9, 11, 12
11, 11′, 11′′	23.39	1.83, m, [1H]; 1.79, m [1H]	9, 10, 11, 12; 9, 10, 11, 12	9, 10, 12	1.81, m, [6H]	28.14, 28.13	9, 10, 12
12, 12′, 12′′	38.93	3.00, m, [2H]	9, 10, 11, 12	10, 11	2.99, m, [6H]	38.9	10, 11
Serine							
13	171.91						169.8
13′							168.6
13′′							168.8
14	54.82	4.55, t (8.0,) [1H]	15	13, 15	4.56, m, [1H]	55.0	8, 13, 15
14′					4.80, m, [1H]	52.0	8′, 13′, 15′
14′′					4.87, m, [1H]	51.7	8′′, 13′′, 15′′
15	61.35	3.98, dd (4.8, 11.2), [1H]; 3.88, dd (4.0, 11.2), [1H]	14, 15	13, 14	3.95, m, [1H]; 3.82, m, [1H]	62.2	13, 14; 13, 14
15′					4.72, m, [1H]; 4.43, m, [1H]	64.5	13, 13′, 14′; 13, 13′, 14′
15′′					4.57, m, [1H]; 4.49, m, [1H]	63.5	13′′, 14′′; 13′′, 14′′

The structures of **2** and **3** were inferred using MS, ^1^H NMR, and 2–D NMR experiments HSQC and HMBC (**2**: Table S1, Figures S15-S20; **3**: Table 5, Figures S21-S26). The ^1^H NMR spectra of **2** and **3** are similar to that of **1**, with the addition of a downfield shift of serine methylene protons of **2** and **3** (δ 4.43 to 4.75) compared to the serine methylene protons of **1** (δ 3.88, 3.98), indicating the presence of serine ester linkages in **2** and **3**.

The MS parent ion masses of **5** suggested a cyclic trimer as the result of an additional serine ester bond, leading to a triserine lactone backbone. However, ^1^H and ^13^C NMR in addition to 2-D experiments ^1^H-^1^H TOCSY, HSQC, and HMBC revealed a dehydrated analogue of the linear trimer (Table S1, Figures S37-S46). The dehydration of the serine hydroxyl group resulted in an alkene, corresponding to a carbon resonance of δ 112.81 and proton resonances of δ 6.12 and 5.80. Similar spectra were obtained for **4** (Table S1, Figures S27-S36).

Chiral amino acid analysis of turnerbactin was carried out using Marfey's method [29]. The hydrolysate of turnerbactin was derivatized with FDAA and compared to amino acid standards derivatized in the same manner (Figure S3), revealing the presence of L-ornithine and L-serine.

A UV-visible spectrum of purified turnerbactin showed an absorption peak at 330 nm, characteristic of catecholate siderophores.

### Disruption of *tnbF* diminishes Fe binding activity

In order to correlate the *tnb* gene cluster with the biosynthesis of turnerbactin, a *tnbF* mutant was constructed by integrating a chloramphenicol (Cm) resistance cassette into the M1 C domain of *tnbF* by a single crossover recombination (Figure 1A). The resulting strain was named TtAH03.

**Figure 4:**
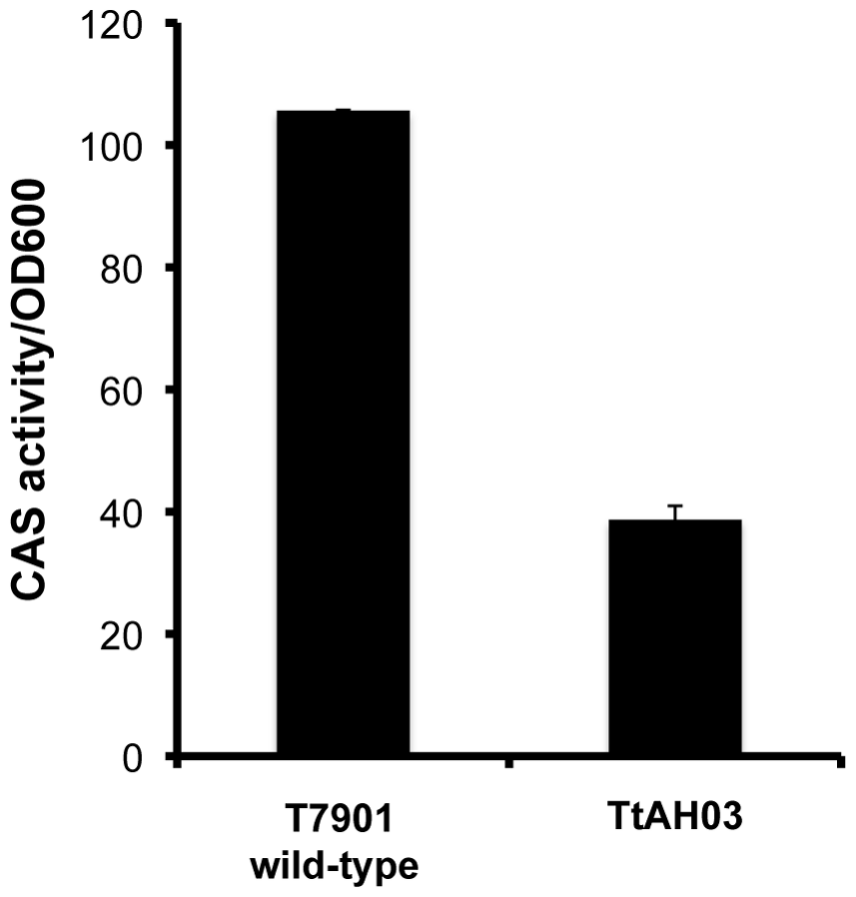
Fe(III)-binding activity of wild-type *T. turnerae* T7901 compared to *tnbF* mutant TtAH03. Disruption of *tnbF* leads to a significant decrease in siderophore activity, as measured by the CAS assay. CAS activity was normalized to OD600 measurements of each culture.

**Figure 5:**
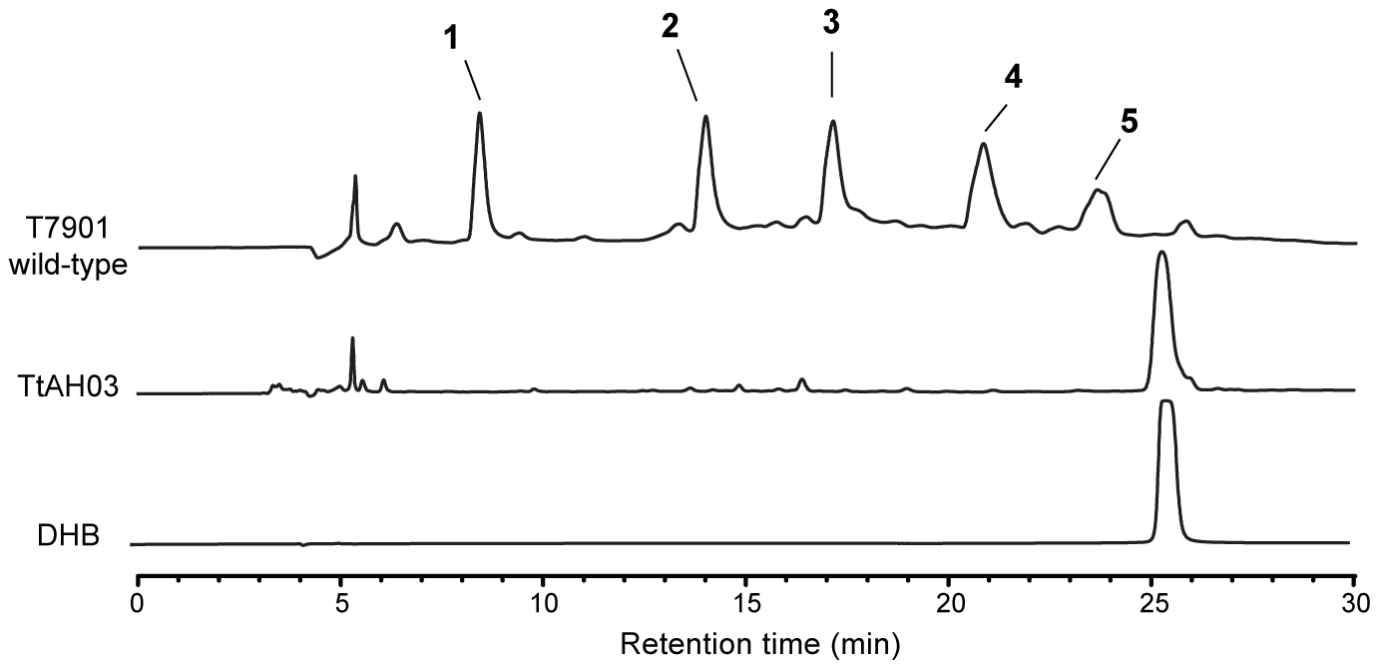
Comparison of wild-type T7901 and TtAH03 extracts by HPLC, recorded at 215 nm.

Disruption of *tnbF* resulted in a significant reduction in Fe(III)-binding activity as measured by the CAS assay compared to wild-type strain T7901 (Figure 4). The residual CAS activity shown for TtAH03 is presumably due to the DHB units produced by the upstream genes *tnbCEBA*. For further support, crude culture extracts from both wild-type T7901 and mutant TtAH03 were compared by HPLC, alongside pure DHB (Figure 5). The extract of wild-type T7901 shows the presence of all five siderophores, as expected. TtAH03 lacks these five peaks while showing a prominent peak at about 25.5 min, corresponding to the retention time of pure DHB. These results support the proposed role of the *tnb* gene cluster in the role of turnerbactin biosynthesis.

### Biosynthesis of turnerbactin

**Figure 6:**
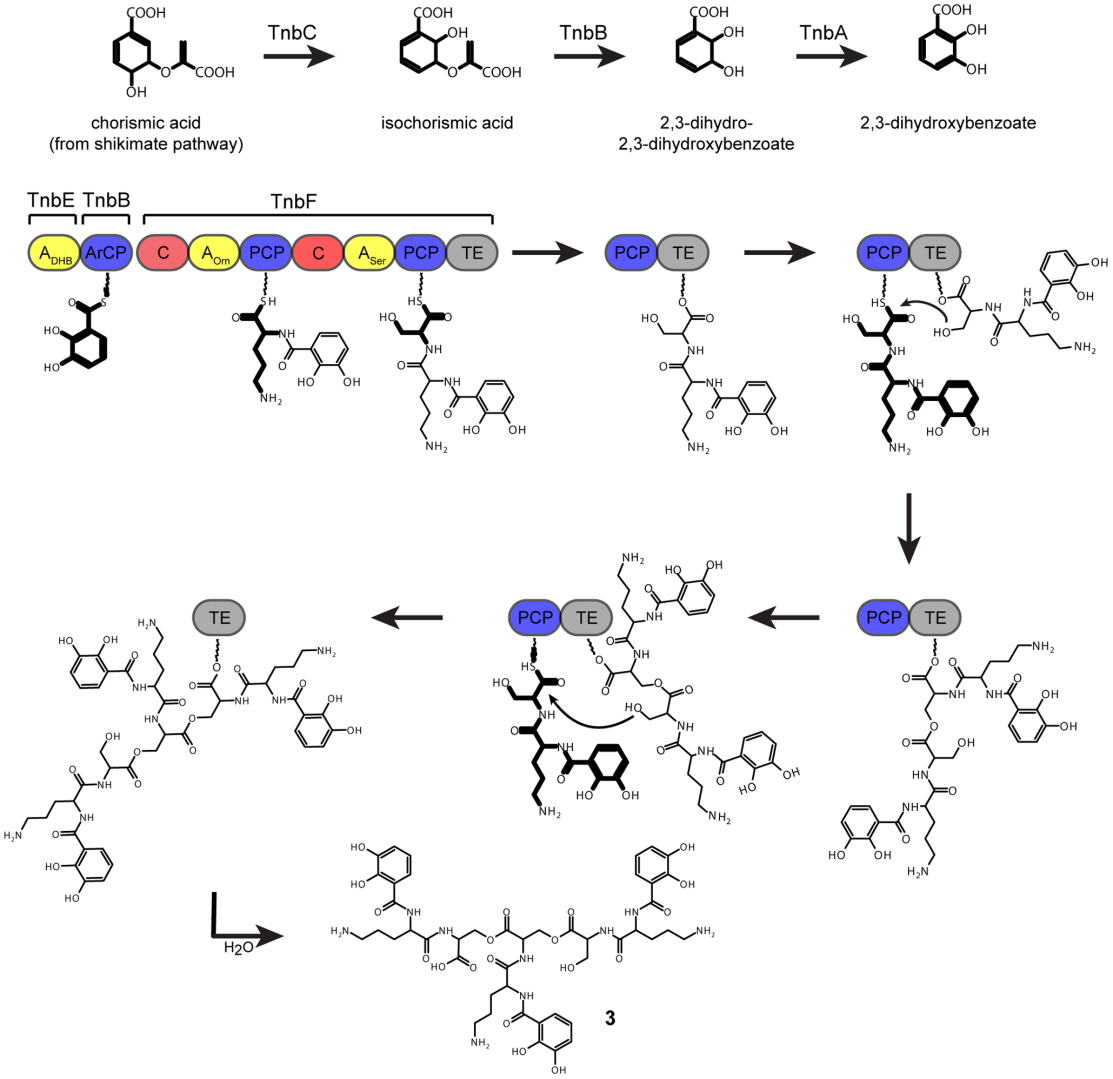
Proposed biosynthesis of turnerbactin. Bold structures indicate the most recent addition to the compound.

The proposed biosynthesis is shown in Figure 6. DHB is activated by TnbE and then transferred to the aryl carrier C-terminal portion of TnbB. L-Orn is activated by the M1 A domain of TnbF and the M1 C domain condenses DHB to Orn, forming a DHB-Orn intermediate on the M1 PCP domain. L-Ser is activated by the M2 A domain and the M2 C domain condenses the DHB-Orn intermediate to the Ser to form the thioester intermediate DHB-Orn-Ser-S-PCP on the M2 PCP domain. A conserved Ser residue in the TE domain mounts a nucleophilic attack on the PCP domain-bound thioester intermediate, leading to a DHB-Orn-Ser-O-TE ester intermediate. After another round of DHB-Orn-Ser biosynthesis, the Ser side chain hydroxyl group of the DHB-Orn-Ser-O-TE intermediate attacks the DHB-Orn-Ser-S-PCP to yield a (DHB-Orn-Ser)_2_-S-PCP, which is then transferred to the TE domain. A third consecutive round yields a (DHB-Orn-Ser)_3_-O-TE intermediate, which is then hydrolyzed by water to yield the linear trimer (DHB-Orn-Ser)_3_, turnerbactin (**3**). It is uncertain whether the other compounds (**1**, **2**, **4**, and **5**) discovered in this study are byproducts from regulated biosynthesis by the NRPS, incomplete biosynthesis by the NRPS, enzymatic degradation by an esterase, or hydrolysis products from either the purification process or culture conditions.

### Detection of turnerbactin in shipworm samples

**Figure 7:**
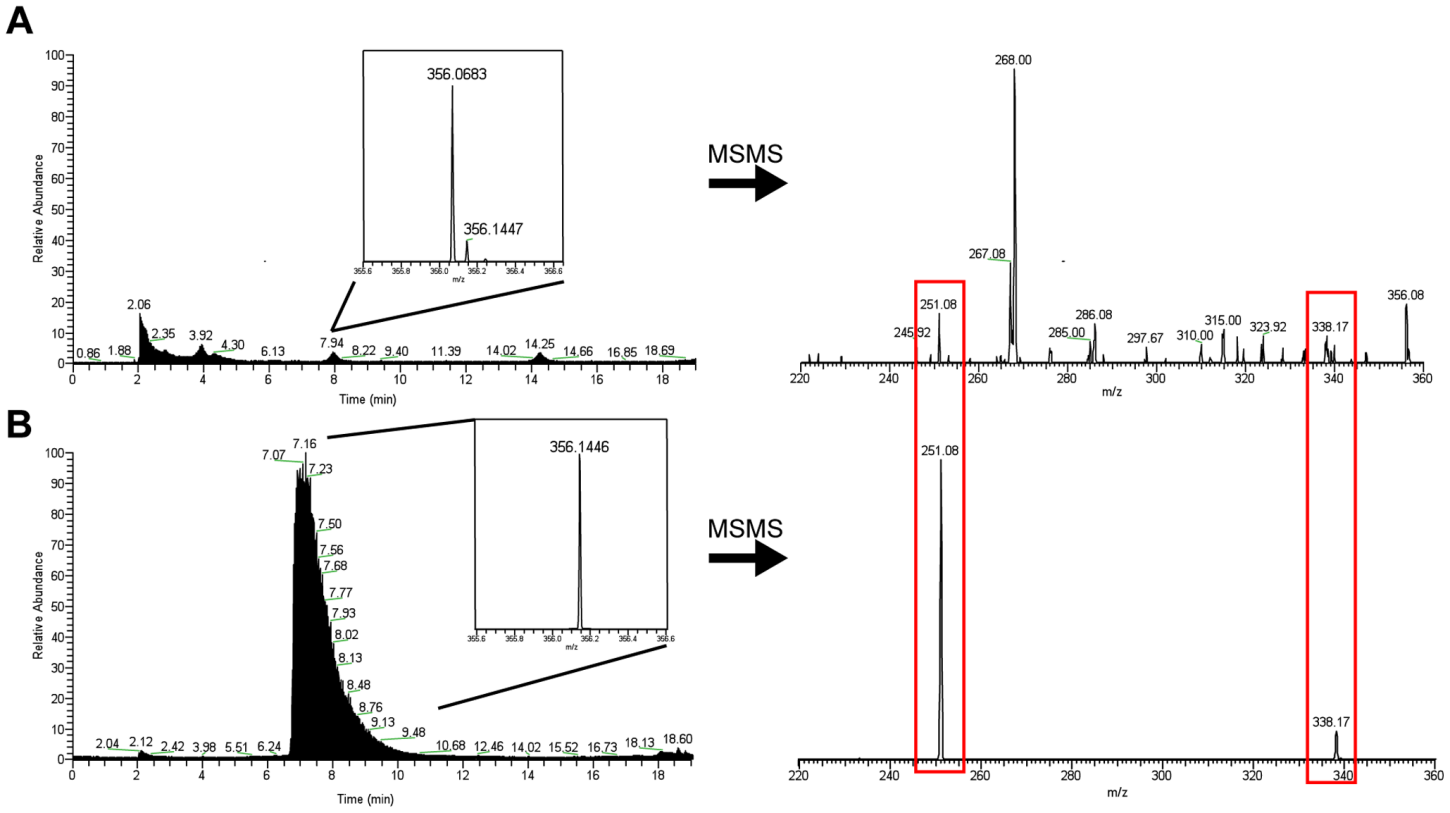
HPLC, HRMS, and MS/MS analysis of *L. pedicellatus* extracts. A. Extract of *L. pedicellatus*. B. Standard of DHB-Orn-Ser, isolated from *T. teredinibacter* T7901. Inset in both figures shows the HRMS of the peak indicated. The shipworm extract shows other compounds with similar mass, but also contains a compound with nearly identical high resolution mass as the DHB-Orn-Ser standard of 356.1446. Red boxes indicate shared MS/MS fragmentation peaks of the 356 molecular ion between the shipworm extract and the siderophore standard.

The potential for the involvement of turnerbactin in the symbiosis between *T. turnerae* and its shipworm host was examined. Detection of the compound in the shipworm host would provide evidence that the compound is produced and utilized in the symbiosis. The presence of turnerbactin and the related DHB-Orn-Ser products in the shipworm host was investigated using MS. The shipworm, *Lyrodus pedicellatus*, is known to contain the symbiont *T. turnerae* [51], [52]. A crude, methanolic extract of whole *L. pedicellatus* tissue samples was analyzed by LC-HRESIMS/MS and compared to pure siderophores isolated from *T. turnerae* T7901 as standards. The method used in this analysis shows pure DHB-Orn-Ser eluting from the column over the span of approximately 7.5–8.5 min (Figure 7). The HRESIMS m/z peak for this elution product is 356.1446. MS/MS analysis shows the presence of two daughter ions with m/z values of 338.17 and 251.08. Analysis of the *L. pedicellatus* extract shows a peak eluting at 7.94 min with an m/z value of 356.1447. MS/MS analysis of this peak shows the presence of the two daughter ions at 338.17 and 251.08. In order to discount contamination of the column, *L. pedicellatus* extracts were run before siderophore standards with a blank injection before each run. Turnerbactin and the other siderophore fragments were not detected in *L. pedicellatus* extracts. The lack of detection of these products may be due to degradation of these products or they may have been present in concentrations below the detection limit of the assay.

## Discussion

This work describes the purification, structural characterization, and biosynthesis genes of a novel siderophore, turnerbactin, from the shipworm symbiont *T. turnerae* T7901. Turnerbactin was isolated along with a monomer, dimer, dehydrated dimer, and dehydrated trimer of DHB-Orn-Ser. We propose that turnerbactin is produced beginning with the production and activation of DHB by the protein products of *tnbCEBA*. The NRPS encoded by *tnbF* then activates and condenses first L-Orn to DHB and then L-Ser to DHB-L-Orn. Turnerbactin is likely assembled by *tnbF* in such a way that the modules are used three consecutive times to produce a trimer of 2,3-DHB-L-Orn-L-Ser before being hydrolytically released by the C-terminal TE domain to yield the linear peptide product.

The cyclic form of turnerbactin was not found in this study. Because previously reported trimeric triscatecholate siderophores have been found in their cyclic form (enterobactin [9], [10], bacillibactin [12], cyclic trichrysobactin [15], streptobactin [16]), with the exception of the recently reported linear trivanchrobactin [18], we wanted to ensure that the lack of the cyclic form of turnerbactin was not an artifact of the purification method. Similarly, we wanted to ensure that the dehydroalanine (Dha) residue resulting from a dehydrated Ser residue, which has not been previously reported in siderophores, was also not an artifact of the purification method. To determine if the lack of a cyclic trimer and the finding of dehydrated products were artifacts of the purification method a control experiment with cyclic trichrysobactin from *D. chrysanthemi* [15] was carried out. The monomeric forms of chrysobactin and turnerbactin both contain a Ser backbone with a DHB functional group and differ only in the spacer amino acid: D-lysine in chrysobactin, L-ornithine in turnerbactin. A side-by-side purification with the same method yielded cyclic trichrysobactin, trichrysobactin, dichrysobactin, chrysobactin, and no dehydrated products from *D. chrysanthemi* while yielding the same suite of siderophores as described in this study from *T. turnerae*. Due to their high degree of structural similarity, this control experiment suggests that the lack of a cyclic trimer is indeed not an artifact of the purification method and the dehydration may be due to an enzymatic modification on the part of *T. turnerae*.

A Dha residue is not unprecedented in other biosynthesized compounds. The formation of Dha residues has been previously reported in ribosomally-produced (RP) compounds such as lantibiotics [53] and thiopeptide antibiotics [54], and in non-RP compounds such as the microcystins [55], [56], [57]. The LanB family of enzymes catalyzes the dehydration of amino acids in the biosynthesis of RP compounds. Using BLAST and pHMM analysis, a potential homolog of this enzyme was not found in the genome of *T. turnerae* T7901. The exact mechanism of non-RP formation of Dha is unclear. However, an enzyme in the biosynthetic pathway of microcystin in *Microcystis aeruginosa*, McyI, with similarity to D-3-phosphoglycerate dehydrogenases, is thought to be responsible for the dehydration of Ser in microcystin [57]. BLAST analysis of McyI against the genome of *T. turnerae* T7901 did identify a D-3-phosphoglycerate dehydrogenase, encoded by the gene TERTU_0393 (S/I, 50%/32%), distantly located from the turnerbactin biosynthetic gene cluster. However, since this enzyme is essential for serine biosynthesis, it is difficult to implicate this enzyme in the dehydration of Ser in turnerbactin. More broadly, a dehydrated amino acid in a siderophore has been previously reported in the loihichelins [58]. The loihichelins are a suite of amphiphilic siderophores from the marine bacterium *Halomonas* sp. LOB-5 and contain dehydroamino-2-butyric acid. However, a mechanism for this dehydration has not been reported. Alternatively, the dehydration of Ser in turnerbactin may occur through a non-enzymatic means, though as noted above, the control experiment with cyclic trichrysobactin suggests that this does not occur during the purification process.

The bioinformatic analyses of *tnbF*'s catalytic domains suggest these domains elude accurate prediction. Predictive analysis of the M1 A domain failed to suggest a specificity with reasonable cut-off scores. C domain analysis suggests a ^D^C_L_ acting domain, while chemical data suggest a ^L^C_L_ acting domain. Taken together, the addition of *tnbF* to the databases of NRPS domains may help to improve the functional prediction of as-yet-undiscovered NRPS domains.

The NRPS's responsible for the production of glycopeptide antibiotics, found in actinomycetes, also exhibit aberrant C domain prediction. Glycopeptide NRPS's contain seven modules. Rausch et al. [34] showed that the M4 and M7 C domains act as ^L^C_L_ domains while clustering in the ^D^C_L_ group of C domains in phylogenetic analysis, similar to the M2 C domain of TnbF (Figure S1). While the M2 C domain of TnbF does not cluster directly with these glycopeptide C domains, a similar change of function is assumed to have occurred, most likely a result of convergent evolution. Occasionally, an external racemase can be found in a biosynthetic gene cluster, providing a D-amino acid for the NRPS. This is the case for cyclosporine, where an external racemase provides D-Ala for the first module of the cyclosporine synthetase [59]. However, an external racemase was not detected in the turnerbactin biosynthetic gene cluster and D-Orn was not detected in amino acid analysis.

Nevertheless, turnerbactin represents a novel siderophore, structurally similar to the catecholate siderophores trivanchrobactin and trichrysobactin. A catecholate siderophore was partially characterized from the soil diazotroph, *Azospirillum brasilense*, which contained equimolar amounts of 2,3-DHB, Orn, and Ser [60]. However, a structure was neither elucidated nor presented. Turnerbactin, vanchrobactin, and chrysobactin share a 2,3-DHB functional moiety, a Ser backbone, and a hydrophilic long, positively charged spacer amino acid. The latter trait distinguishes these siderophores from the widely studied triscatecholate enterobactin. However, the biosynthesis of turnerbactin differs from vanchrobactin and chrysobactin in that the spacer amino acid is not epimerized to a D-configuration, due to the absence of an E domain in TnbF. The similarity of TnbF to the vanchrobactin and chrysobactin NRPS's and the finding of a ^D^C_L_-like C domain for the M2 of TnbF suggest that an E domain may have been lost at some point.

The recent reports of trivanchrobactin [18], cyclic trichrysobactin [15], and streptobactin [16], along with the current reporting of turnerbactin, add to the growing family of triscatecholate siderophores. As mentioned previously, the triscatecholate siderophores contain the highest stability constants for Fe(III) measured for siderophores to date [9], [10]. When comparing a cyclic compound to its linear counterpart, a cyclic compound will likely have a higher stability constant, as the flexibility of the cyclic ligand and its corresponding iron complex will be less than that of the linear ligand, thereby decreasing the entropy difference. This is seen in the stability constants for cyclic enterobactin (10^49^) compared to its linearized form, the linear trimer of DHB-serine (10^43^) [61]. However, the linear trimer still provides a siderophore with relatively strong affinity for iron and the initial rates of uptake for both the linear trimer and linear dimer ferric enterobactin complexes are essentially the same as the cyclic ferric enterobactin complex [61]. The role of iron in regulating the production of turnerbactin is supported by the identification of two putative Fur box sequences in the biosynthetic gene cluster and the increased Fe(III)-binding activity of *T. turnerae* T7901 cultures in response to iron-limited conditions.

A fragment of turnerbactin, DHB-Orn-Ser (**1**), was detected in extracts of the shipworm *L. pedicellatus*. The compound detected in the shipworm shared the same LC retention time, high-resolution molecular ion mass, and daughter ions in tandem MS fragmentation as a pure DHB-Orn-Ser standard. The detection of this monomer unit in a shipworm sample suggests that turnerbactin is produced and utilized in some capacity in the symbiosis. Since whole animal tissue was used in these experiments, it is not known whether these siderophores are confined to the immediate vicinity of the symbionts in the gills, or if they appear in other locations of the shipworm. The exact role of endosymbiont-derived siderophores in the host is currently unclear. The caecum of *L. pedicellatus* is largely devoid of microbes [62]. The mechanism by which this occurs is currently unknown. It has been proposed that secondary metabolites produced by gill endosymbionts such as *T. turnerae* may be translocated to the caecum and contribute to suppression of microbial growth [24]. Siderophores may also play a role in suppression of microbial growth in the caecum. Studies in plant systems have found that siderophores can play a role in nutrient deprivation [63]. By sequestering the iron, siderophores can make iron unavailable to competing microbes, thereby restricting their growth. This theory could be tested by MS-based screening of dissected organs and tissues of shipworms samples. In addition, chemical localization studies using methods such as MS-imaging would allow the localization of the siderophore within host tissue. These types of studies would be valuable in determining the extent to which siderophores are utilized in the host and lend insight into their possible roles, such as intersymbiont competition or suppressing competing microbes in the caecum.

## Supporting Information

Figure S1 **Maximum likelihood tree of C domains, showing the grouping of different C domain subtypes.** The C domains of TnbF are shown in red. TnbF's M2 C domain groups with the ^D^C_L_ functional group, while the M1 C domain groups with the Starter functional group. Each C domain is labeled with the organism name, followed by accession number, followed by the module number from which the C domain is referring. C domains from glycopeptide antibiotic NRPS's are shown in blue. Bootstrap values are based on 100 replicates and are only shown for the basal branches of groups.
(TIF)

Figure S2 **HPLC trace of HP20 extract from** ***T. turnerae*** **T7901 culture supernatant, recorded at 215 nm.**
(TIF)

Figure S3 **HPLC trace of derivatized hydrolysate of turnerbactin and chiral amino acid standards, recorded at 340 nm.** (A) hydrolysate of turnerbactin, (B) L-ornithine, (C) DL-ornithine, (D) L-serine, (1) D-ornithine, (2) L-ornithine, (3) L-serine, (4) Marfey's reagent (FDAA).
(TIF)

Figure S4 **1 ^1^H NMR spectrum (800 MHz) in CD_3_OD.**
(TIF)

Figure S5 **1 ^13^C NMR spectrum (800 MHz) in CD_3_OD.**
(TIF)

Figure S6 **1 ^1^H-^13^C HSQC spectrum (800 MHz) in CD_3_OD.**
(TIF)

Figure S7 **1 ^1^H-^13^C HMBC spectrum (800 MHz) in CD_3_OD.**
(TIF)

Figure S8 **1 ^1^H-^13^C HMBC spectrum (800 MHz) in CD_3_OD, expanded region.**
(TIF)

Figure S9 **1 ^1^H-^13^C HMBC spectrum (800 MHz) in CD_3_OD, expanded region.**
(TIF)

Figure S10 **1 ^1^H-^13^C HMBC spectrum (800 MHz) in CD_3_OD, expanded region.**
(TIF)

Figure S11 **1 ^1^H-^1^H TOCSY spectrum (800 MHz) in CD_3_OD.**
(TIF)

Figure S12 **1 ^1^H-^1^H TOCSY spectrum (800 MHz) in CD_3_OD, expanded region.**
(TIF)

Figure S13 **1 ^1^H-^1^H TOCSY spectrum (800 MHz) in CD_3_OD, expanded region.**
(TIF)

Figure S14 **1 ^1^H-^1^H TOCSY spectrum (800 MHz) in CD_3_OD, expanded region.**
(TIF)

Figure S15 **2 ^1^H NMR spectrum (800 MHz) in CD_3_OD.**
(TIF)

Figure S16 **2 ^1^H-^13^C HSQC spectrum (800 MHz) in CD_3_OD.**
(TIF)

Figure S17 **2 ^1^H-^13^C HMBC spectrum (800 MHz) in CD_3_OD.**
(TIF)

Figure S18 **2 ^1^H-^13^C HMBC spectrum (800 MHz) in CD_3_OD, expanded region.**
(TIF)

Figure S19 **2 ^1^H-^13^C HMBC spectrum (800 MHz) in CD_3_OD, expanded region.**
(TIF)

Figure S20 **2 ^1^H-^13^C HMBC spectrum (800 MHz) in CD_3_OD, expanded region.**
(TIF)

Figure S21 **3 ^1^H NMR spectrum (800 MHz) in CD_3_OD.**
(TIF)

Figure S22 **3 ^1^H-^13^C HSQC spectrum (800 MHz) in CD_3_OD.**
(TIF)

Figure S23 **3 ^1^H-^13^C HMBC spectrum (800 MHz) in CD_3_OD.**
(TIF)

Figure S24 **3 ^1^H-^13^C HMBC spectrum (800 MHz) in CD_3_OD, expanded region.**
(TIF)

Figure S25 **3 ^1^H-^13^C HMBC spectrum (800 MHz) in CD_3_OD, expanded region.**
(TIF)

Figure S26 **3 ^1^H-^13^C HMBC spectrum (800 MHz) in CD_3_OD, expanded region.**
(TIF)

Figure S27 **4 ^1^H NMR spectrum (800 MHz) in CD_3_OD.**
(TIF)

Figure S28 **4 ^13^C NMR spectrum (800 MHz) in CD_3_OD.**
(TIF)

Figure S29 **4 ^1^H-^13^C HMBC spectrum (800 MHz) in CD_3_OD.**
(TIF)

Figure S30 **4 ^1^H-^13^C HMBC spectrum (800 MHz) in CD_3_OD, expanded region.**
(TIF)

Figure S31 **4 ^1^H-^13^C HMBC spectrum (800 MHz) in CD_3_OD, expanded region.**
(TIF)

Figure S32 **4 ^1^H-^13^C HMBC spectrum (800 MHz) in CD_3_OD, expanded region.**
(TIF)

Figure S33 **4 ^1^H-^1^H TOCSY spectrum (800 MHz) in CD_3_OD.**
(TIF)

Figure S34 **4 ^1^H-^1^H TOCSY spectrum (800 MHz) in CD_3_OD, expanded region.**
(TIF)

Figure S35 **4 ^1^H-^1^H TOCSY spectrum (800 MHz) in CD_3_OD, expanded region.**
(TIF)

Figure S36 **4 ^1^H-^1^H TOCSY spectrum (800 MHz) in CD_3_OD, expanded region.**
(TIF)

Figure S37 **5 ^1^H NMR spectrum (800 MHz) in CD_3_OD.**
(TIF)

Figure S38 **5 ^13^C NMR spectrum (800 MHz) in CD_3_OD.**
(TIF)

Figure S39 **5 ^1^H-^13^C HMBC spectrum (800 MHz) in CD_3_OD.**
(TIF)

Figure S40 **5 ^1^H-^13^C HMBC spectrum (800 MHz) in CD_3_OD, expanded region.**
(TIF)

Figure S41 **5 ^1^H-^13^C HMBC spectrum (800 MHz) in CD_3_OD, expanded region.**
(TIF)

Figure S42 **5 ^1^H-^13^C HMBC spectrum (800 MHz) in CD_3_OD, expanded region.**
(TIF)

Figure S43 **5 ^1^H-^1^H TOCSY spectrum (800 MHz) in CD_3_OD.**
(TIF)

Figure S44 **5 ^1^H-^1^H TOCSY spectrum (800 MHz) in CD_3_OD, expanded region.**
(TIF)

Figure S45 **5 ^1^H-^1^H TOCSY spectrum (800 MHz) in CD_3_OD, expanded region.**
(TIF)

Figure S46 **5 ^1^H-^1^H TOCSY spectrum (800 MHz) in CD_3_OD, expanded region.**
(TIF)

Table S1 **NMR data for (DHB-Orn-Ser)_2_ (2), Dehydrated (DHB-Orn-Ser)_2_ (4), and Dehydrated (DHB-Orn-Ser)_3_ (5) (800 MHz) in CD_3_OD.**
(PDF)
