# Supplementary figures and images for: Turnerbactin, a Novel Triscatecholate Siderophore from the Shipworm Endosymbiont Teredinibacter turnerae T7901
Source: PLoS One. 2013 Oct 11;8(10):e76151. doi: 10.1371/journal.pone.0076151 (PMC3795760; doi:10.1371/journal.pone.0076151)

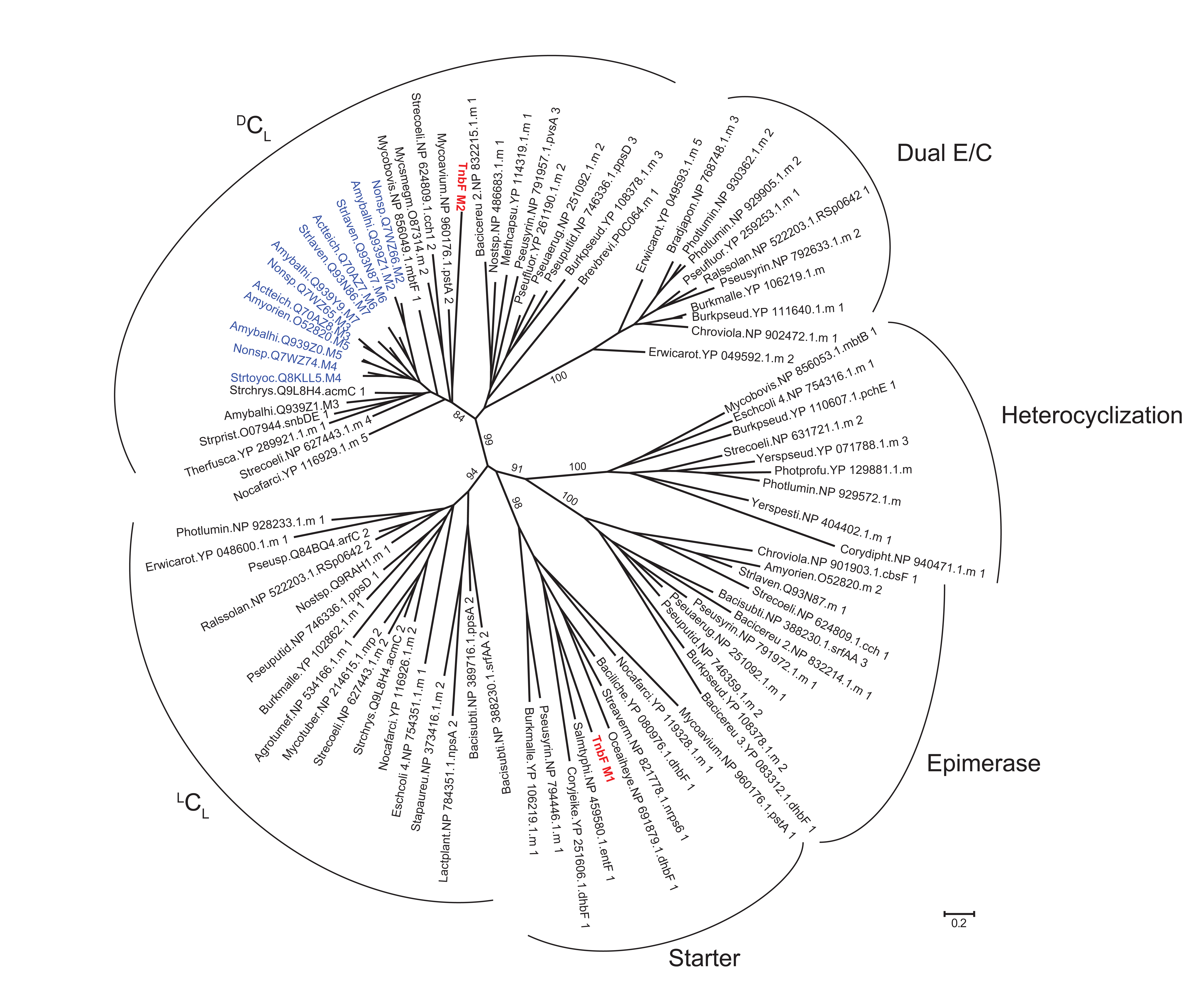

Supplement: Figure S1 — Maximum likelihood tree of C domains, showing the grouping of different C domain subtypes. The C domains of TnbF are shown in red. TnbF's M2 C domain groups with the DCL functional group, while the M1 C domain groups with the Starter functional group. Each C domain is labeled with the organism name, followed by accession number, followed by the module number from which the C domain is referring. C domains from glycopeptide antibiotic NRPS's are shown in blue. Bootstrap values are based on 100 replicates and are only shown for the basal branches of groups. (TIF) [file pone.0076151.s001.tif]

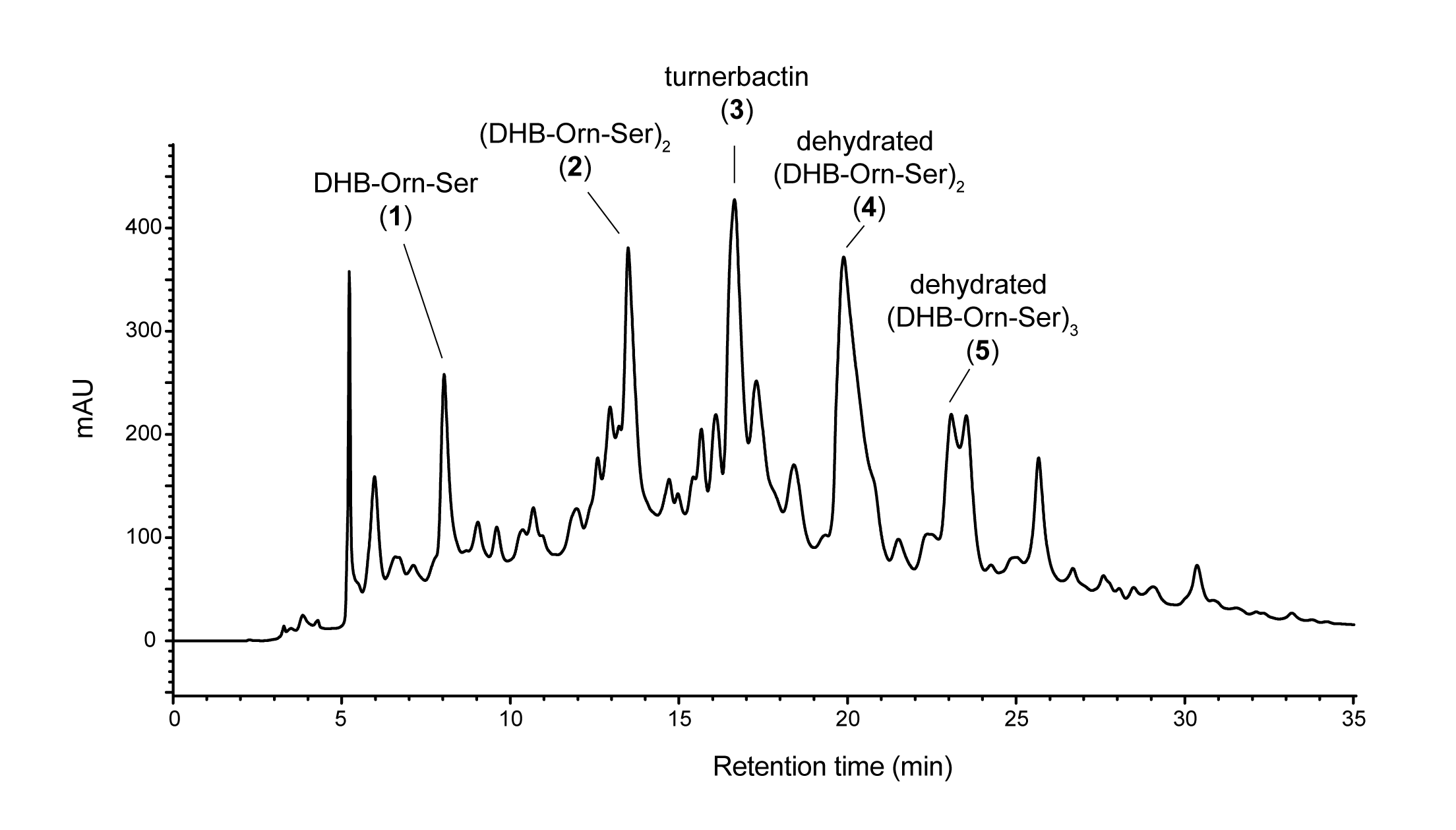

Supplement: Figure S2 — HPLC trace of HP20 extract from T. turnerae T7901 culture supernatant, recorded at 215 nm. (TIF) [file pone.0076151.s002.tif]

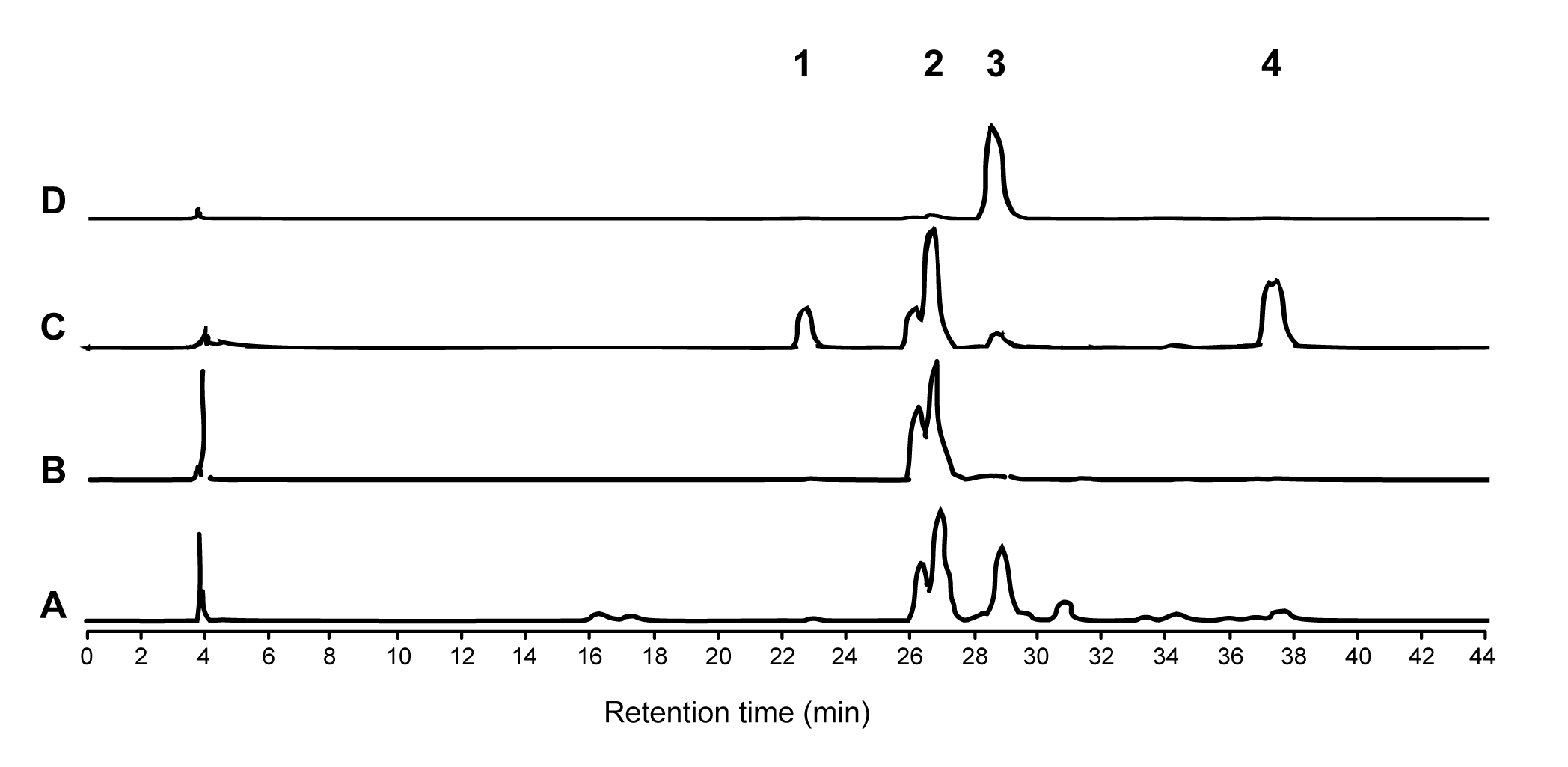

Supplement: Figure S3 — HPLC trace of derivatized hydrolysate of turnerbactin and chiral amino acid standards, recorded at 340 nm. (A) hydrolysate of turnerbactin, (B) L-ornithine, (C) DL-ornithine, (D) L-serine, (1) D-ornithine, (2) L-ornithine, (3) L-serine, (4) Marfey's reagent (FDAA). (TIF) [file pone.0076151.s003.tif]

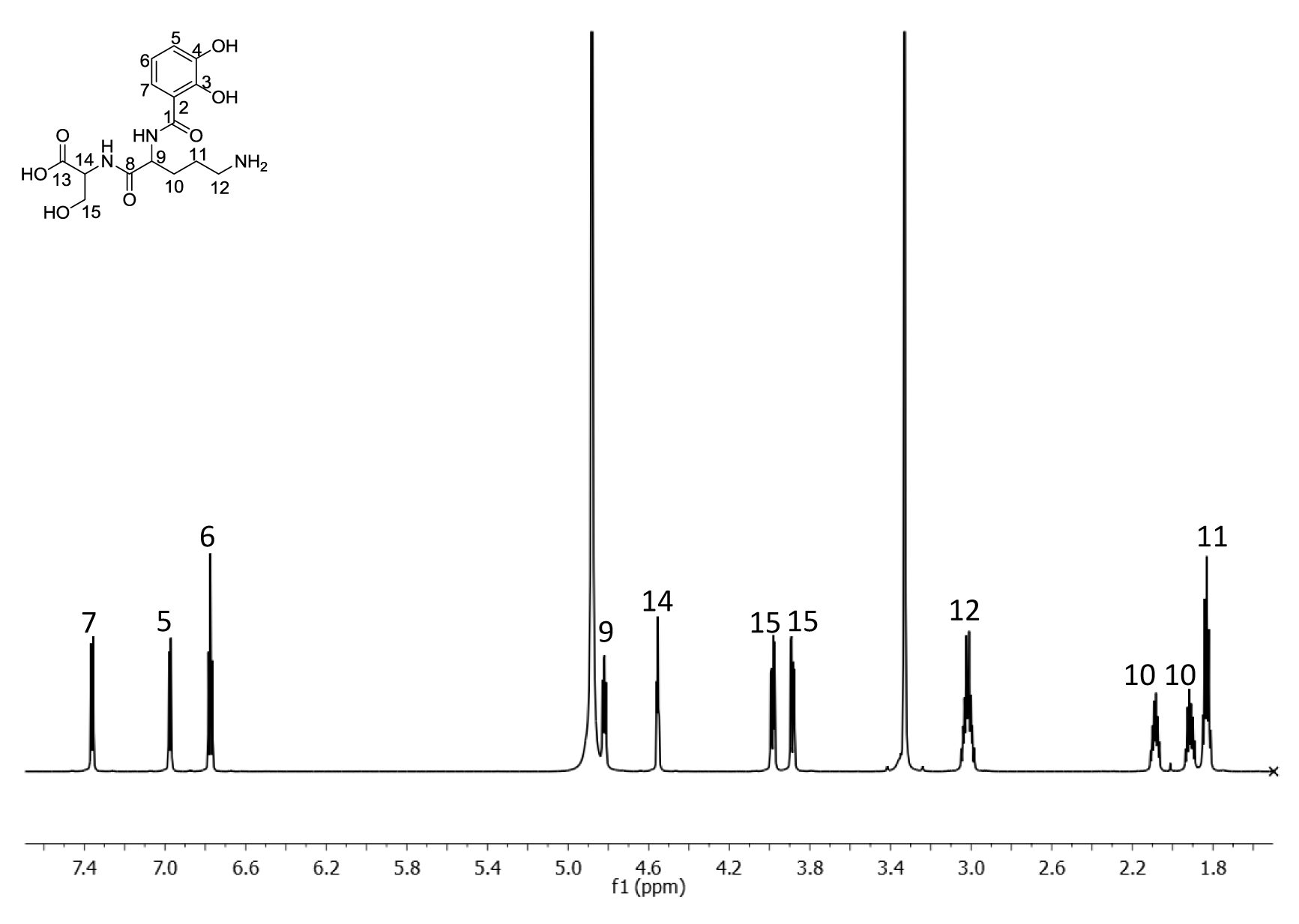

Supplement: Figure S4 — 1 1H NMR spectrum (800 MHz) in CD3OD. (TIF) [file pone.0076151.s004.tif]

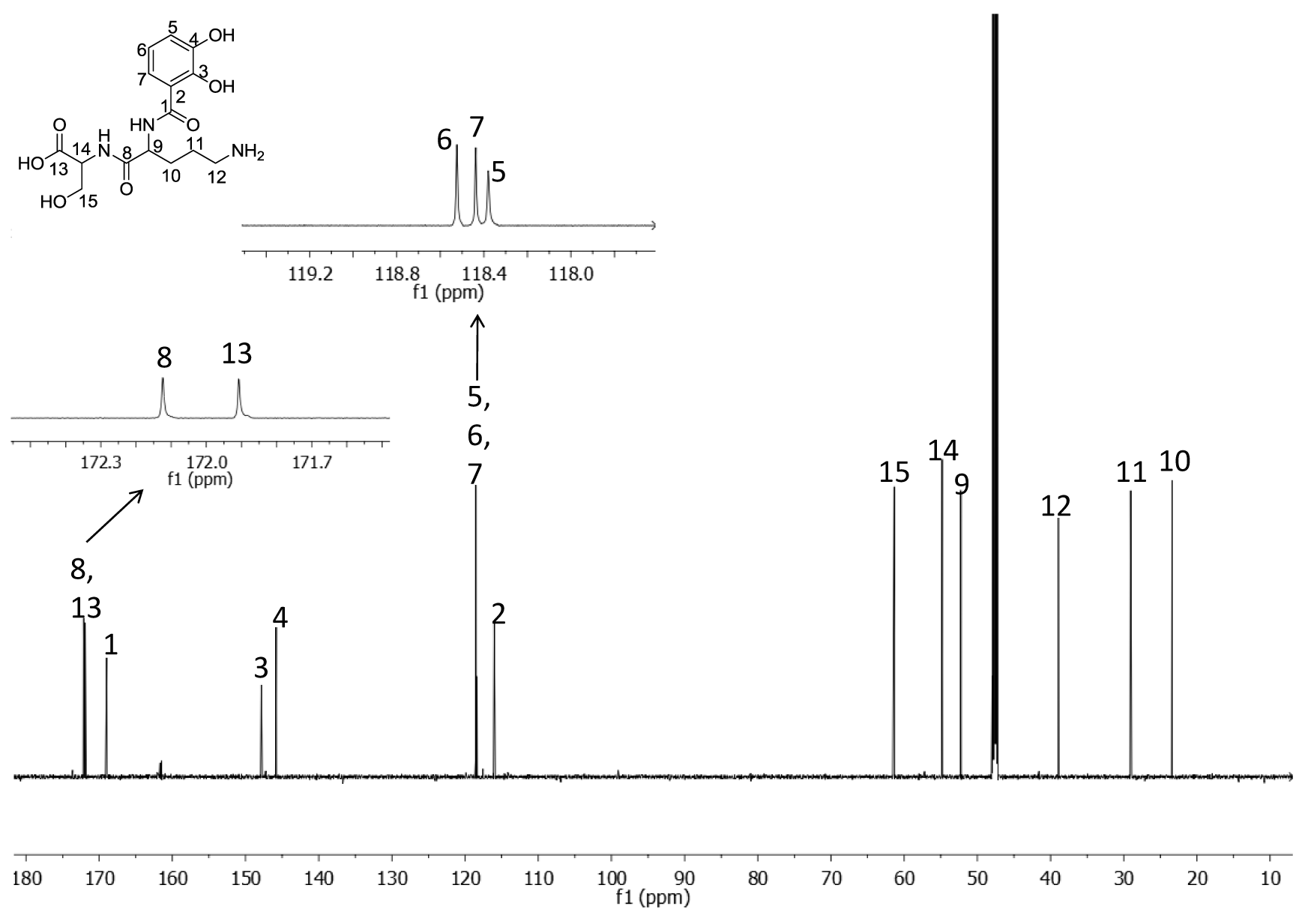

Supplement: Figure S5 — 1 13C NMR spectrum (800 MHz) in CD3OD. (TIF) [file pone.0076151.s005.tif]

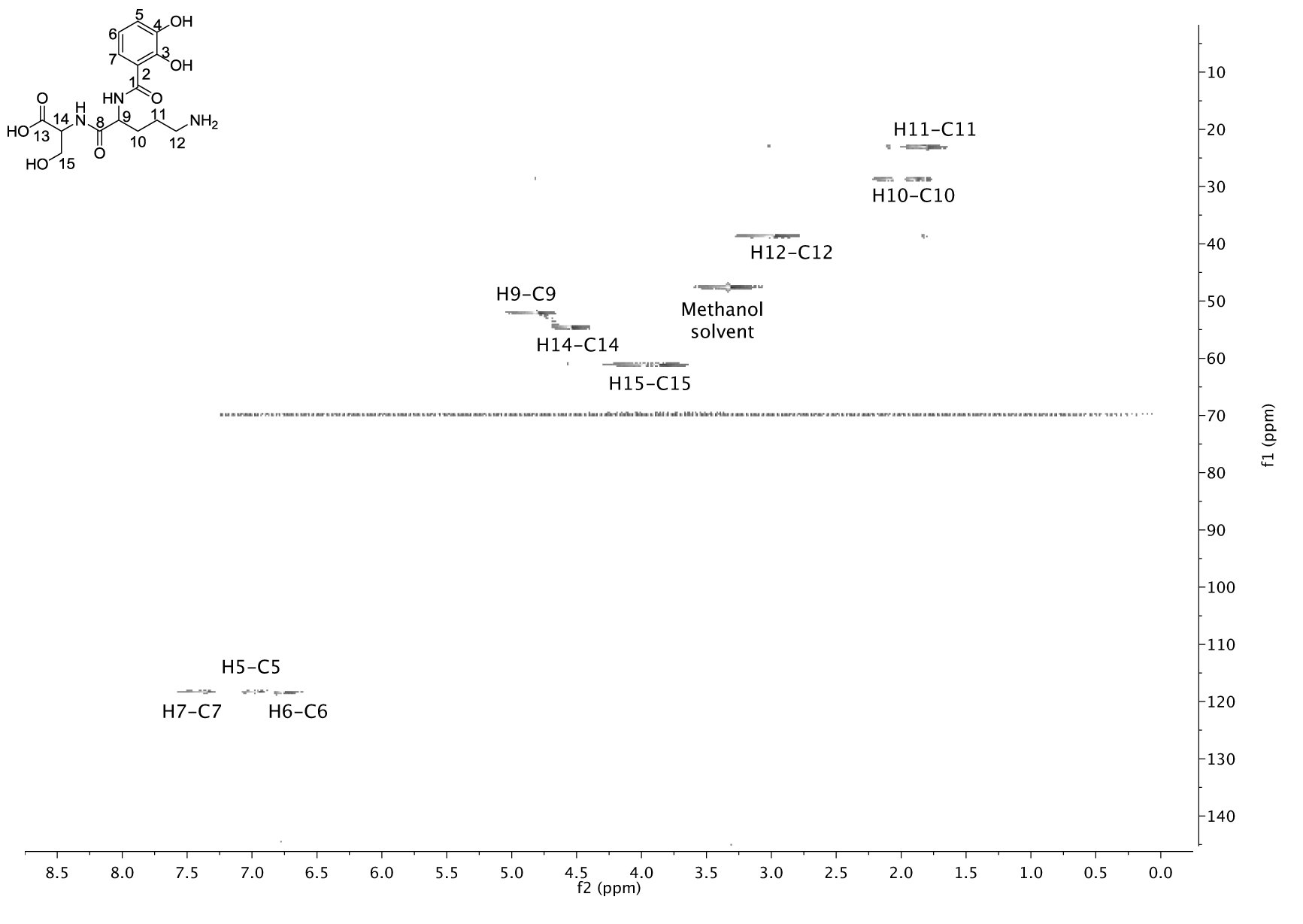

Supplement: Figure S6 — 1 1H-13C HSQC spectrum (800 MHz) in CD3OD. (TIF) [file pone.0076151.s006.tif]

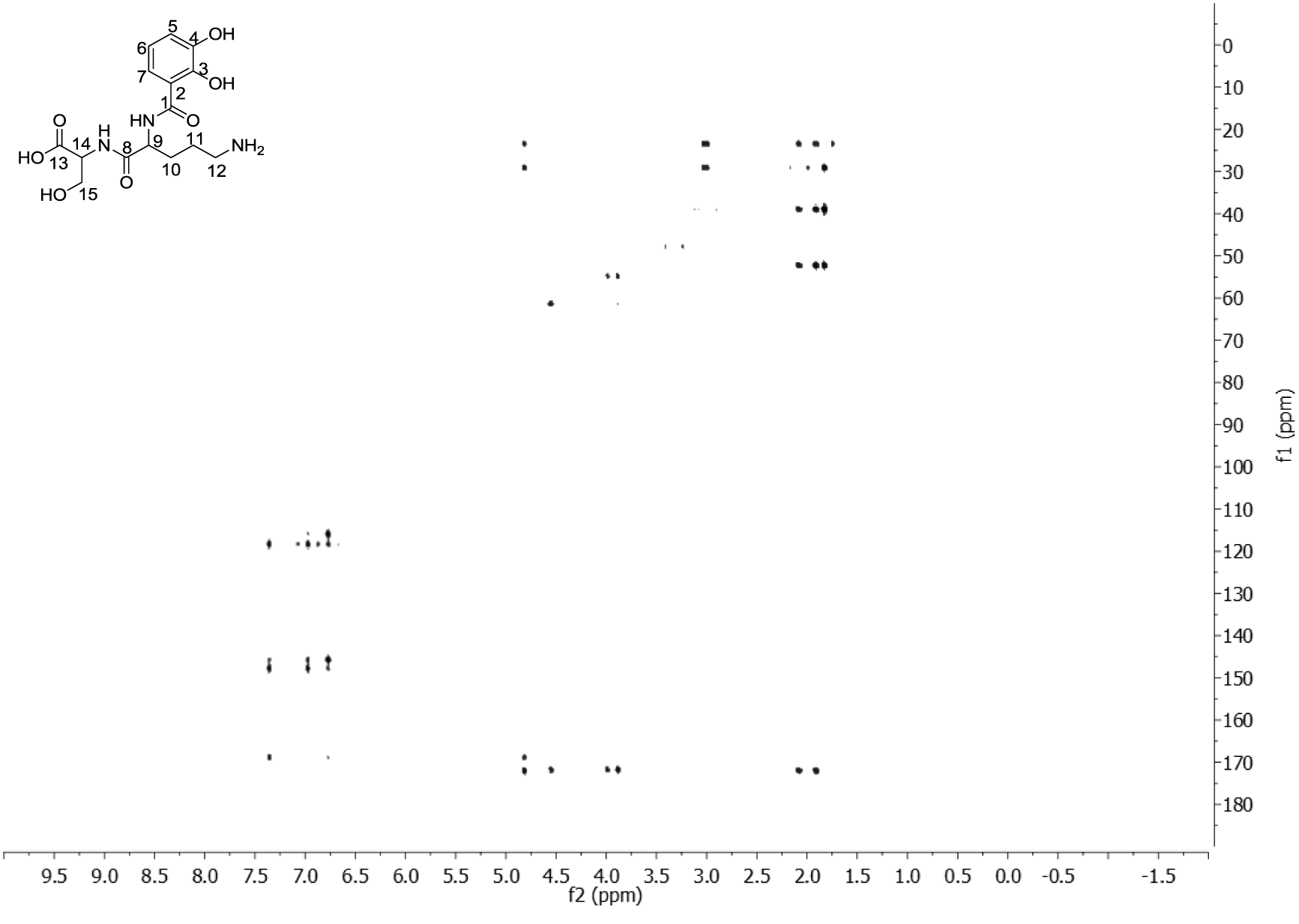

Supplement: Figure S7 — 1 1H-13C HMBC spectrum (800 MHz) in CD3OD. (TIF) [file pone.0076151.s007.tif]

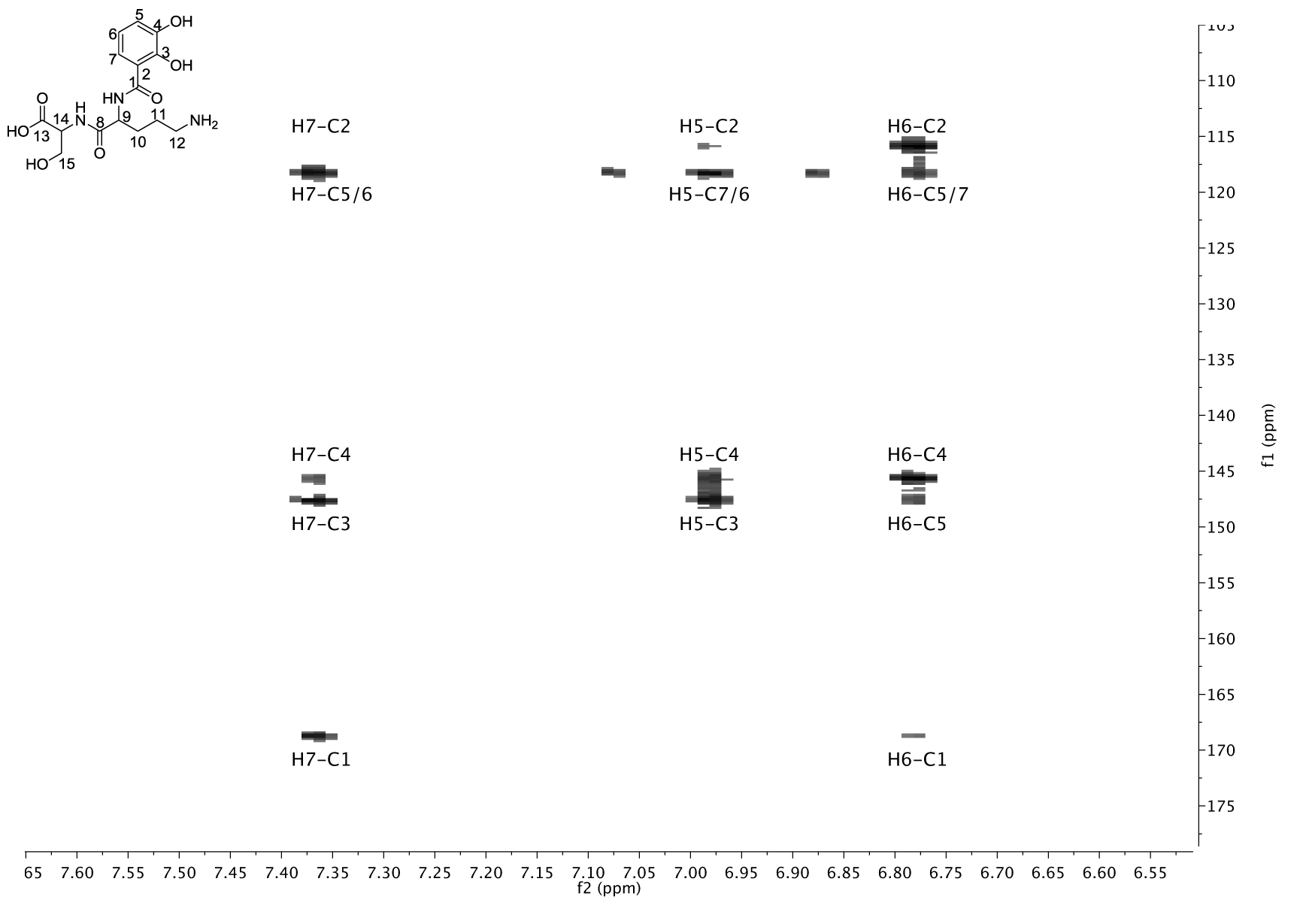

Supplement: Figure S8 — 1 1H-13C HMBC spectrum (800 MHz) in CD3OD, expanded region. (TIF) [file pone.0076151.s008.tif]

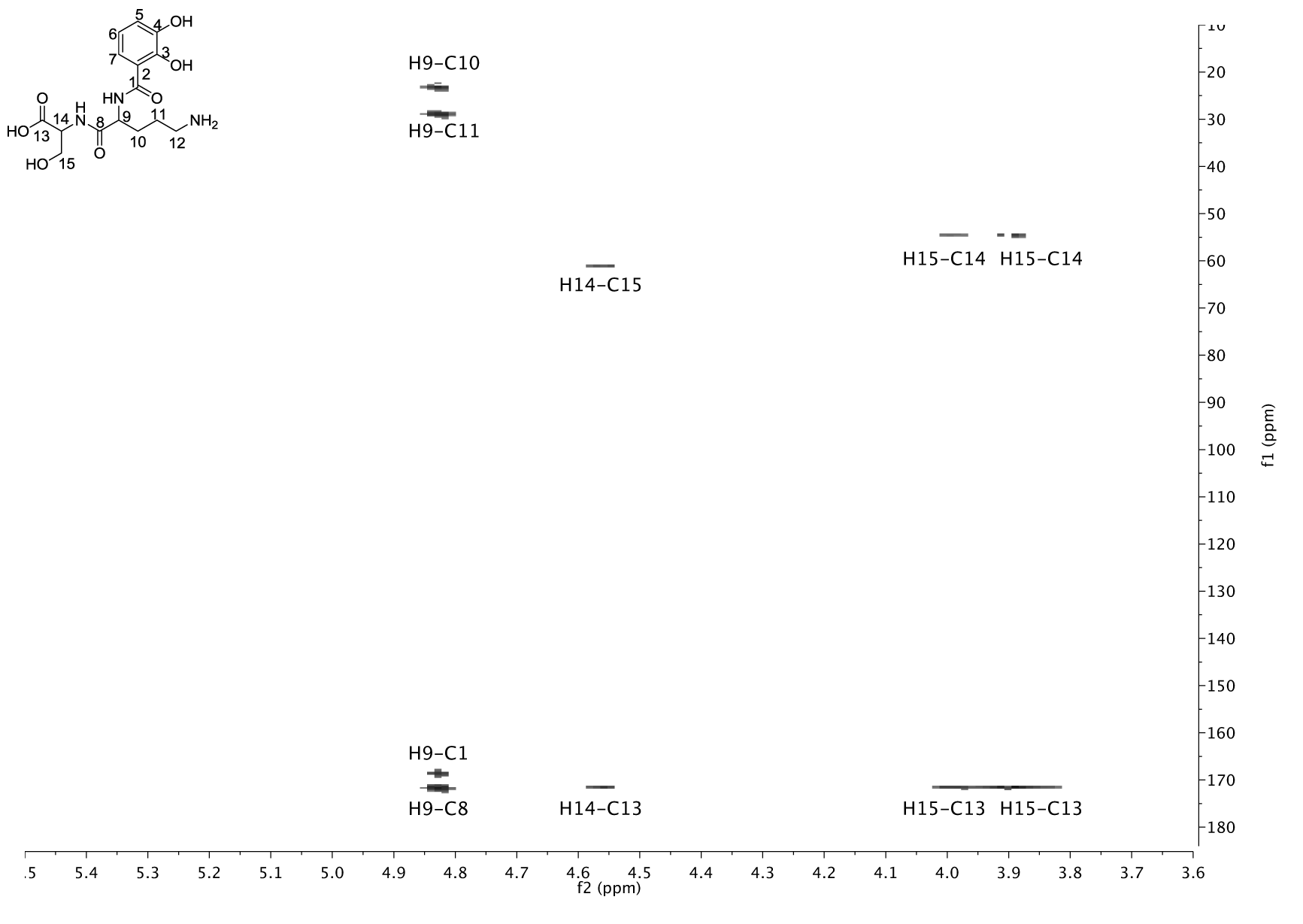

Supplement: Figure S9 — 1 1H-13C HMBC spectrum (800 MHz) in CD3OD, expanded region. (TIF) [file pone.0076151.s009.tif]

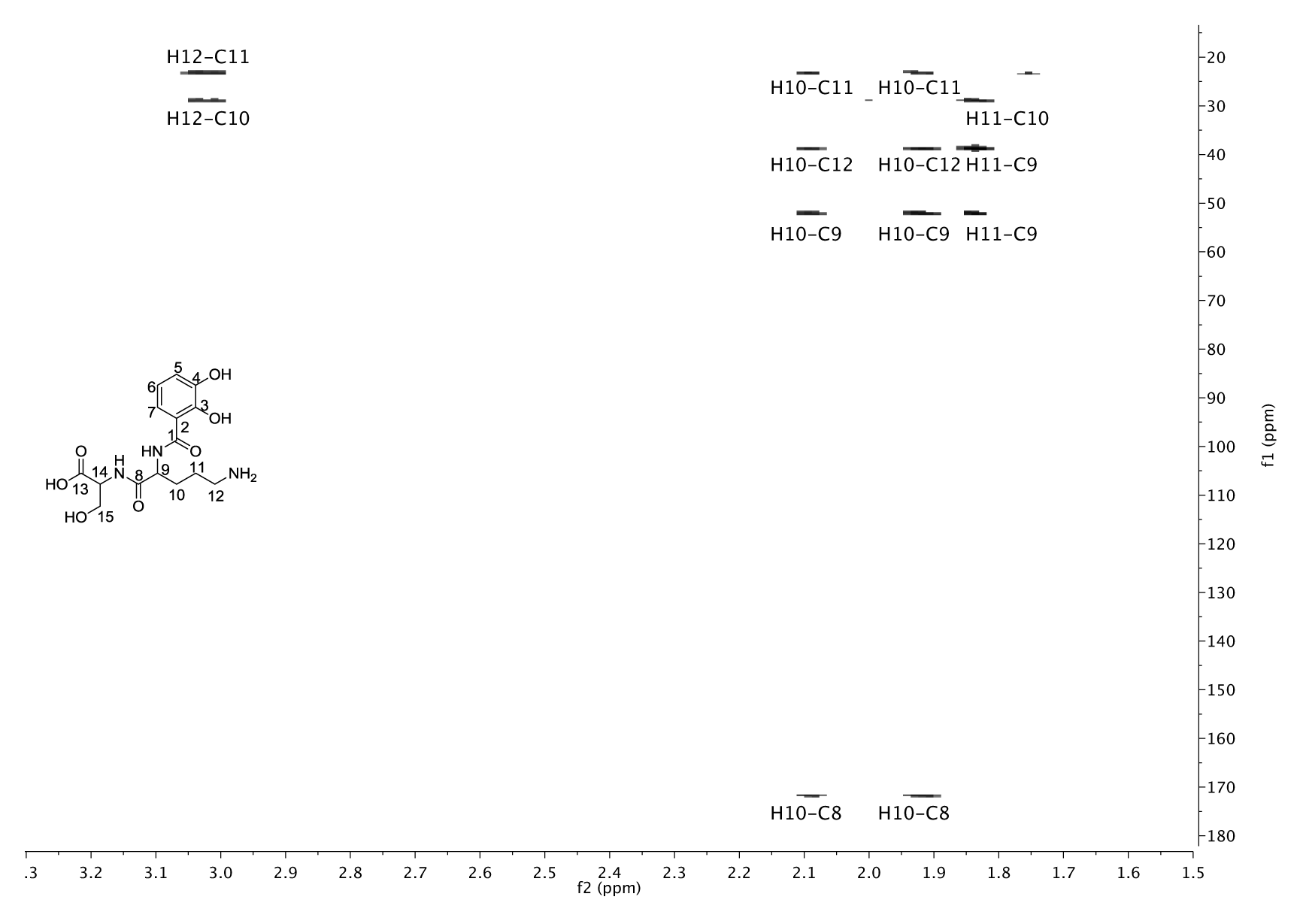

Supplement: Figure S10 — 1 1H-13C HMBC spectrum (800 MHz) in CD3OD, expanded region. (TIF) [file pone.0076151.s010.tif]

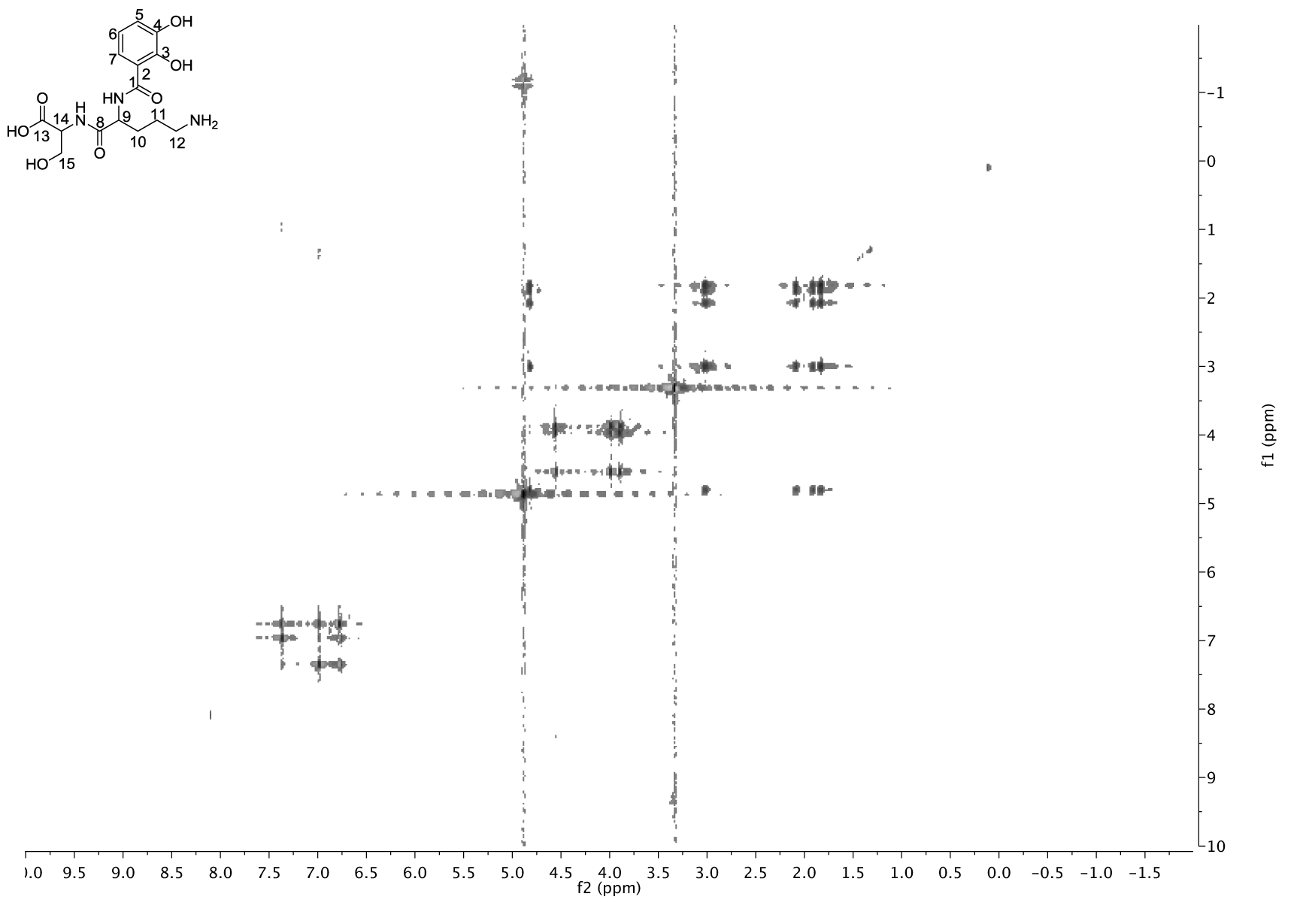

Supplement: Figure S11 — 1 1H-1H TOCSY spectrum (800 MHz) in CD3OD. (TIF) [file pone.0076151.s011.tif]

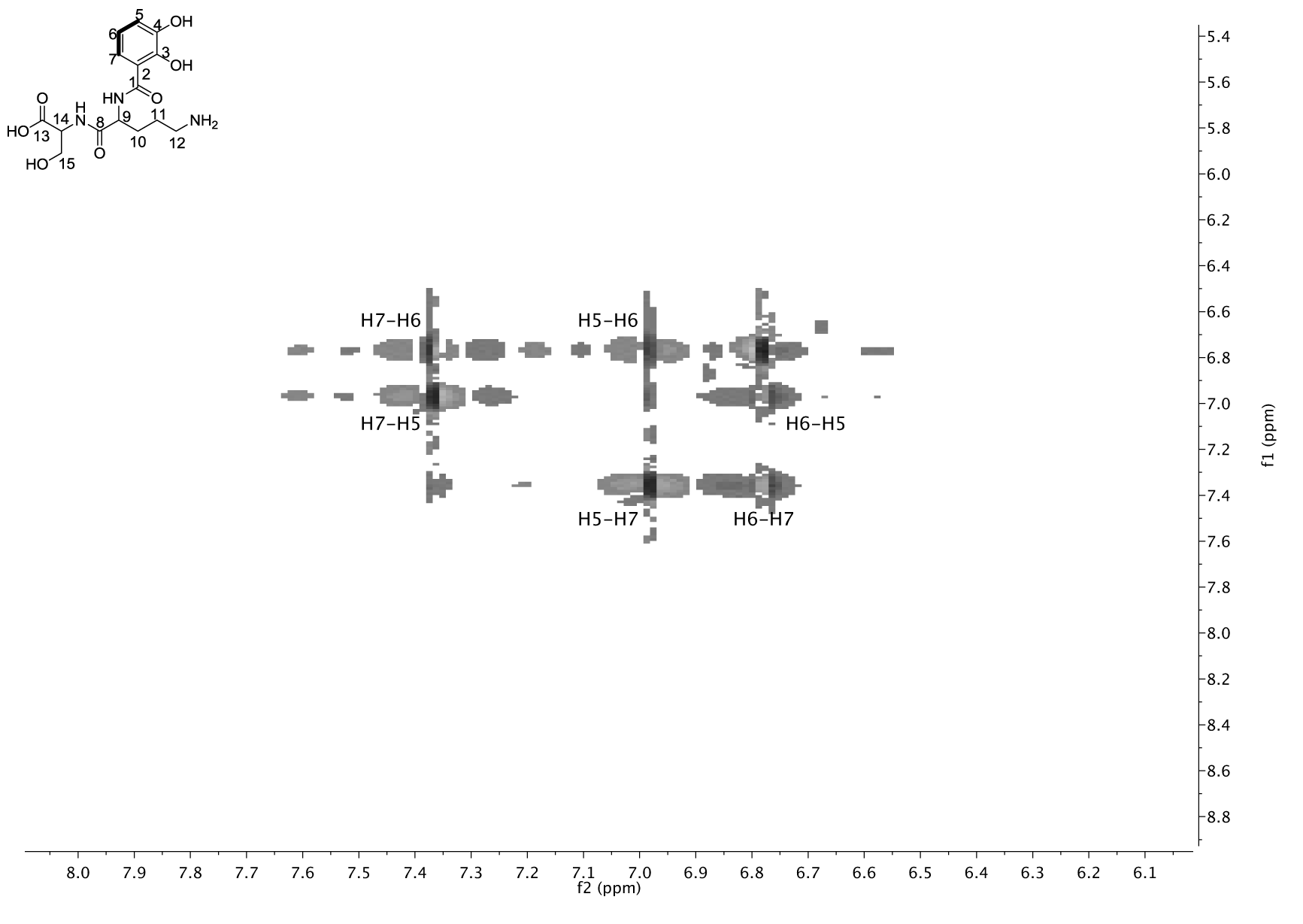

Supplement: Figure S12 — 1 1H-1H TOCSY spectrum (800 MHz) in CD3OD, expanded region. (TIF) [file pone.0076151.s012.tif]

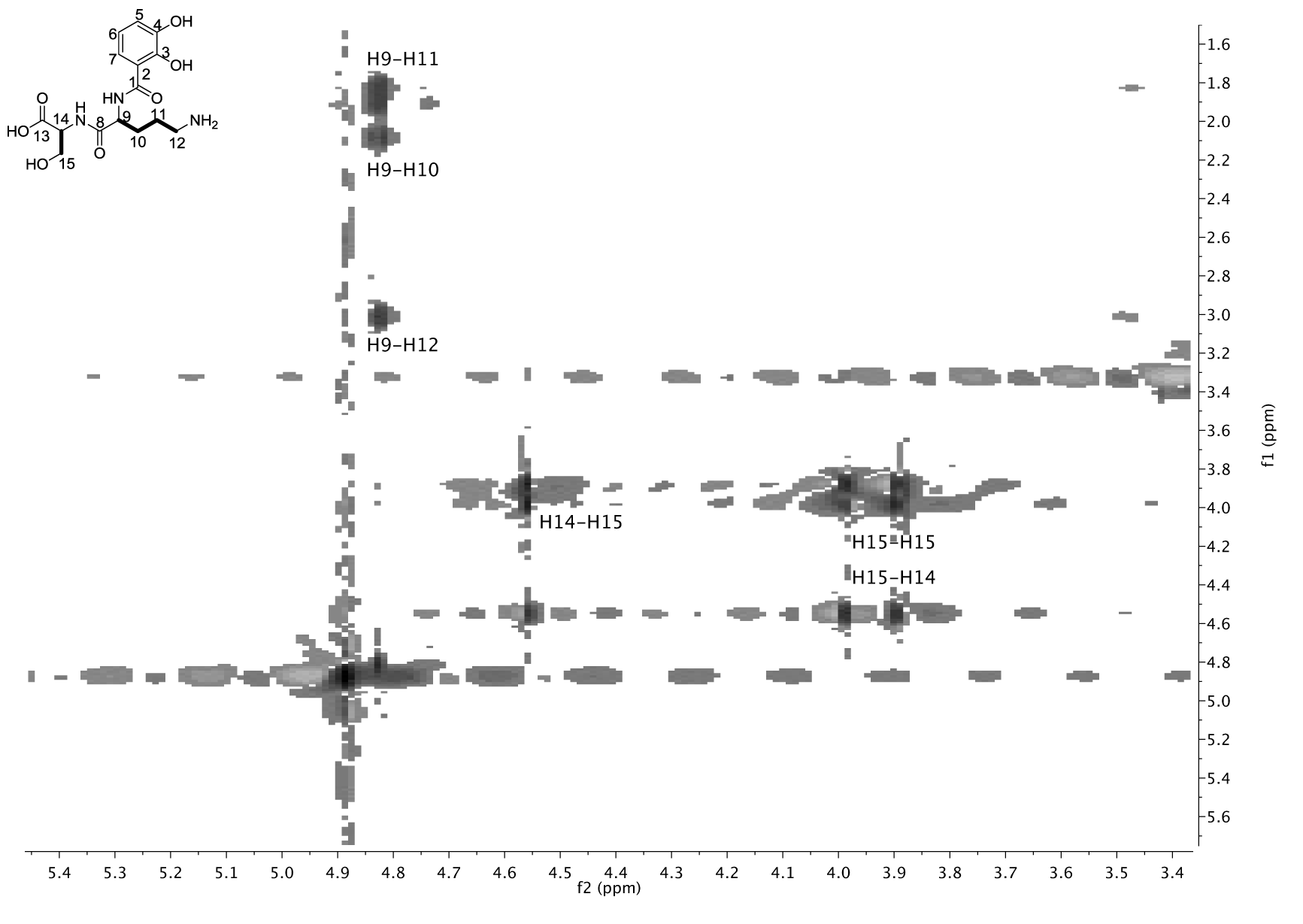

Supplement: Figure S13 — 1 1H-1H TOCSY spectrum (800 MHz) in CD3OD, expanded region. (TIF) [file pone.0076151.s013.tif]

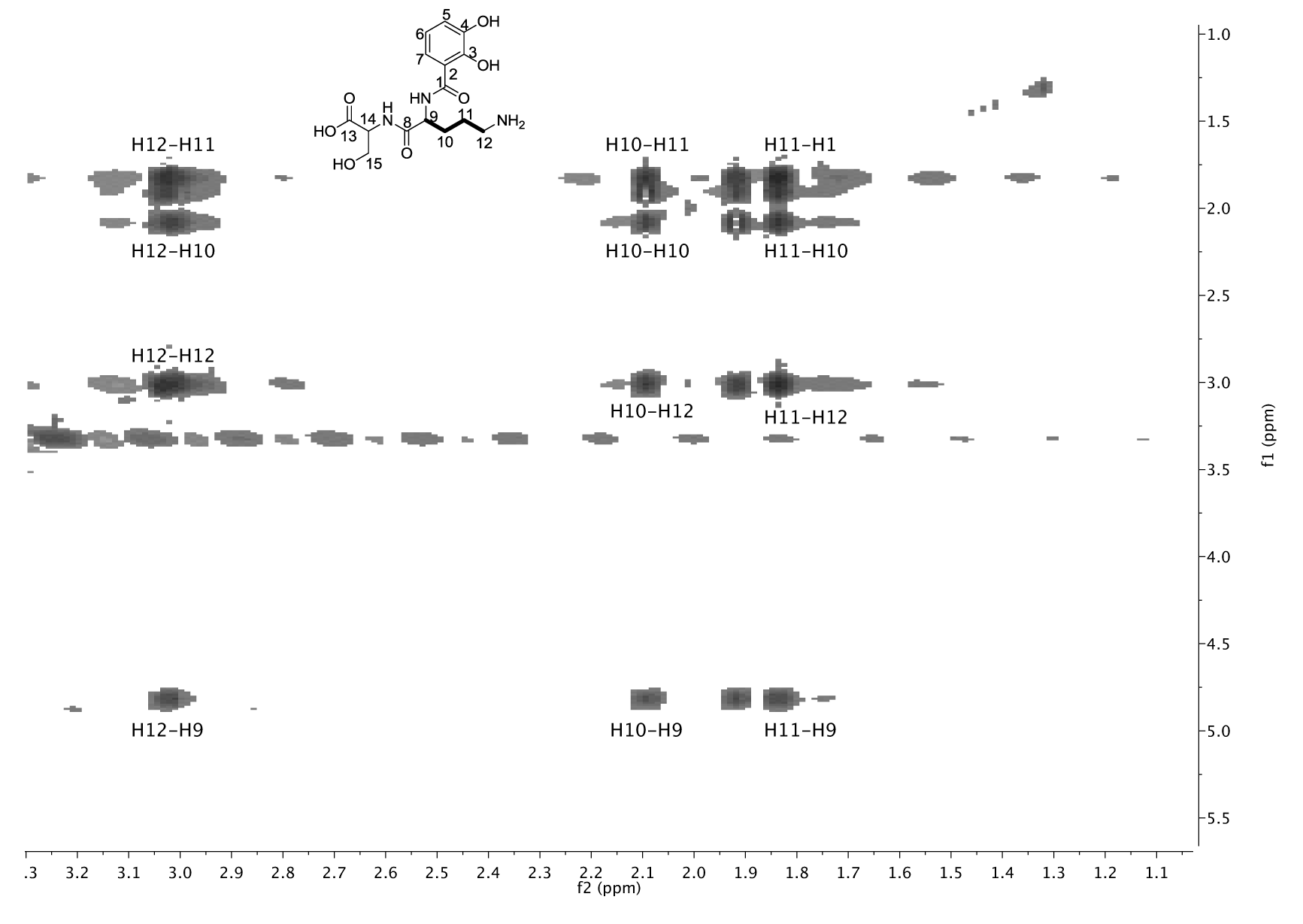

Supplement: Figure S14 — 1 1H-1H TOCSY spectrum (800 MHz) in CD3OD, expanded region. (TIF) [file pone.0076151.s014.tif]

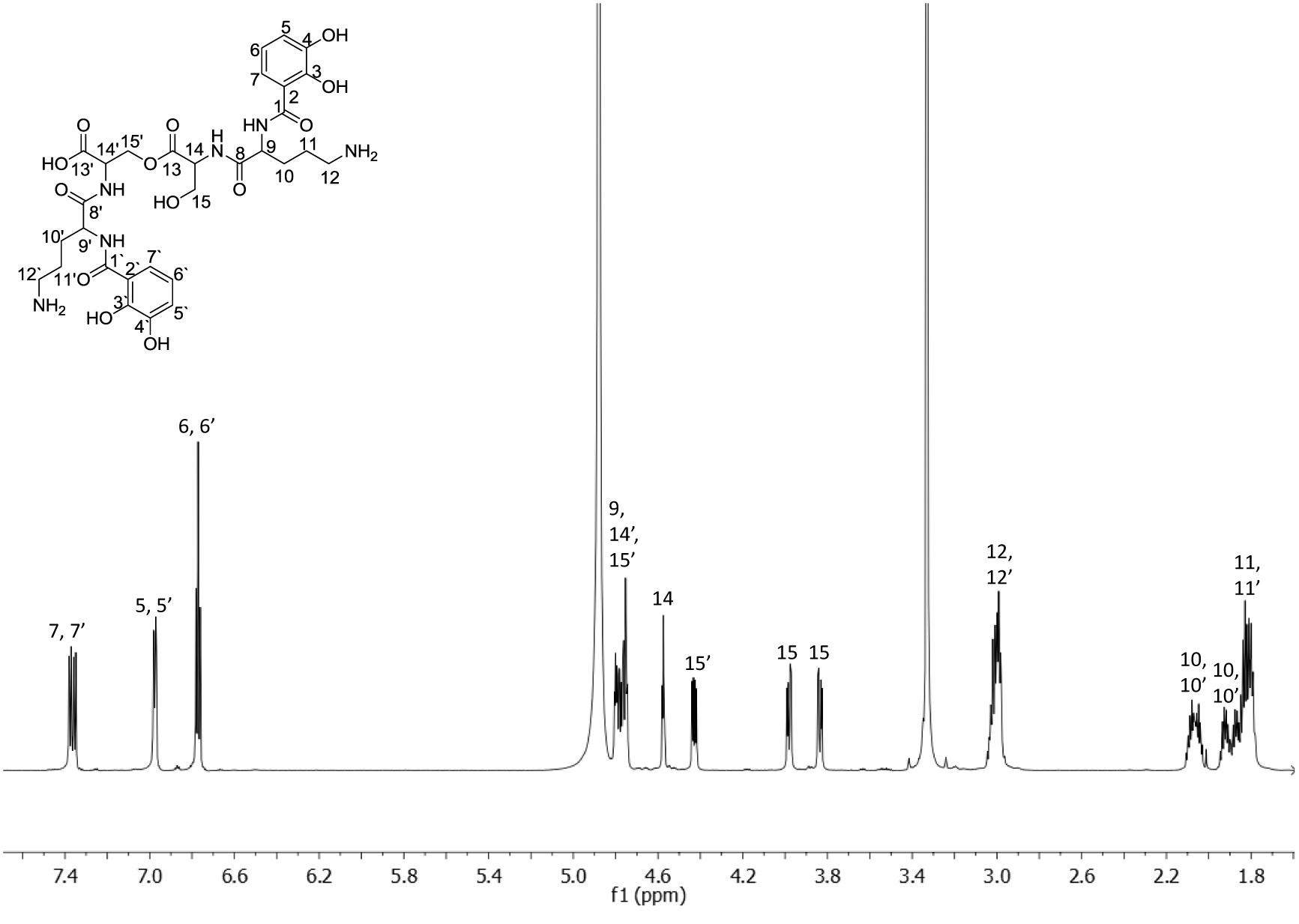

Supplement: Figure S15 — 2 1H NMR spectrum (800 MHz) in CD3OD. (TIF) [file pone.0076151.s015.tif]

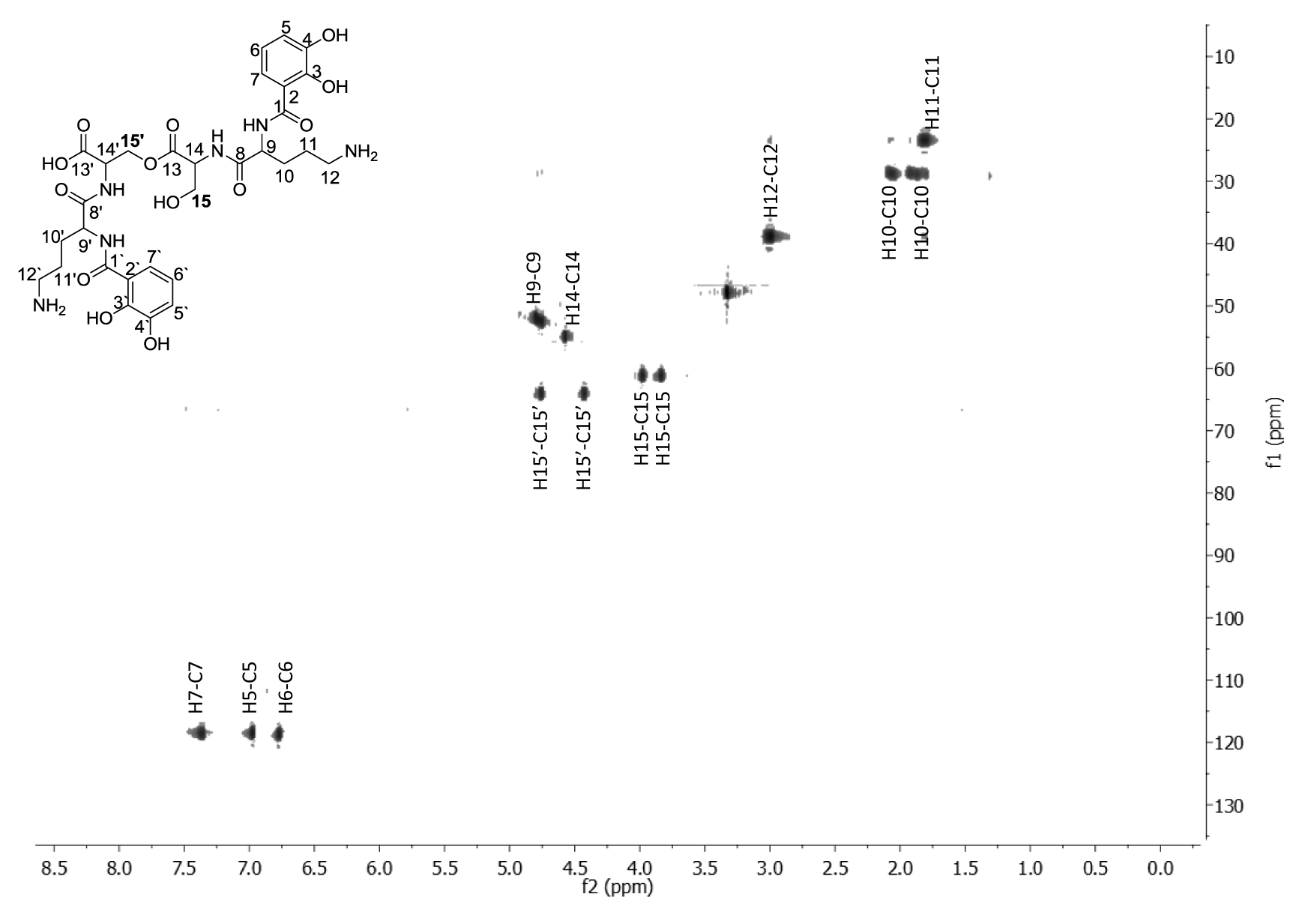

Supplement: Figure S16 — 2 1H-13C HSQC spectrum (800 MHz) in CD3OD. (TIF) [file pone.0076151.s016.tif]

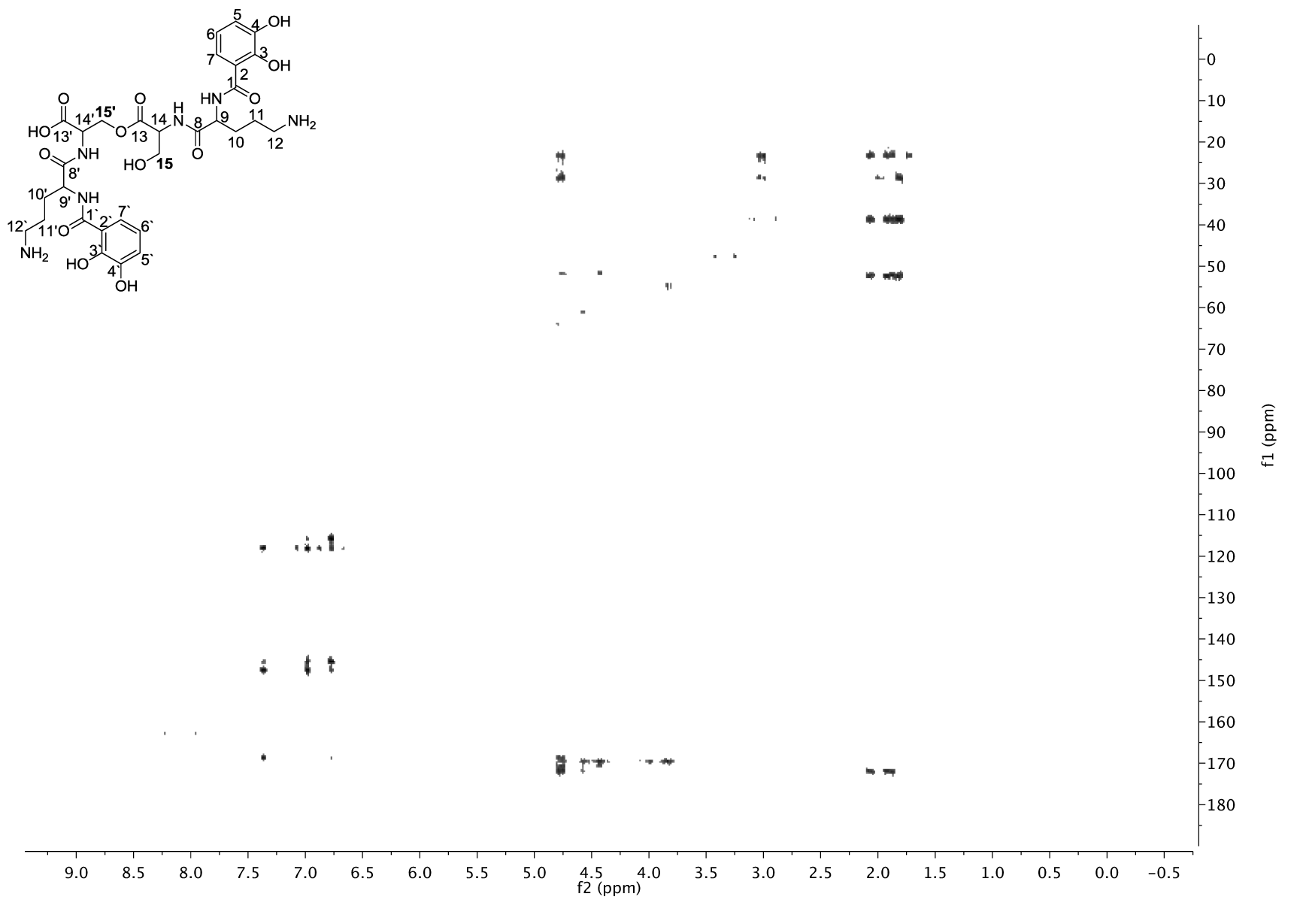

Supplement: Figure S17 — 2 1H-13C HMBC spectrum (800 MHz) in CD3OD. (TIF) [file pone.0076151.s017.tif]

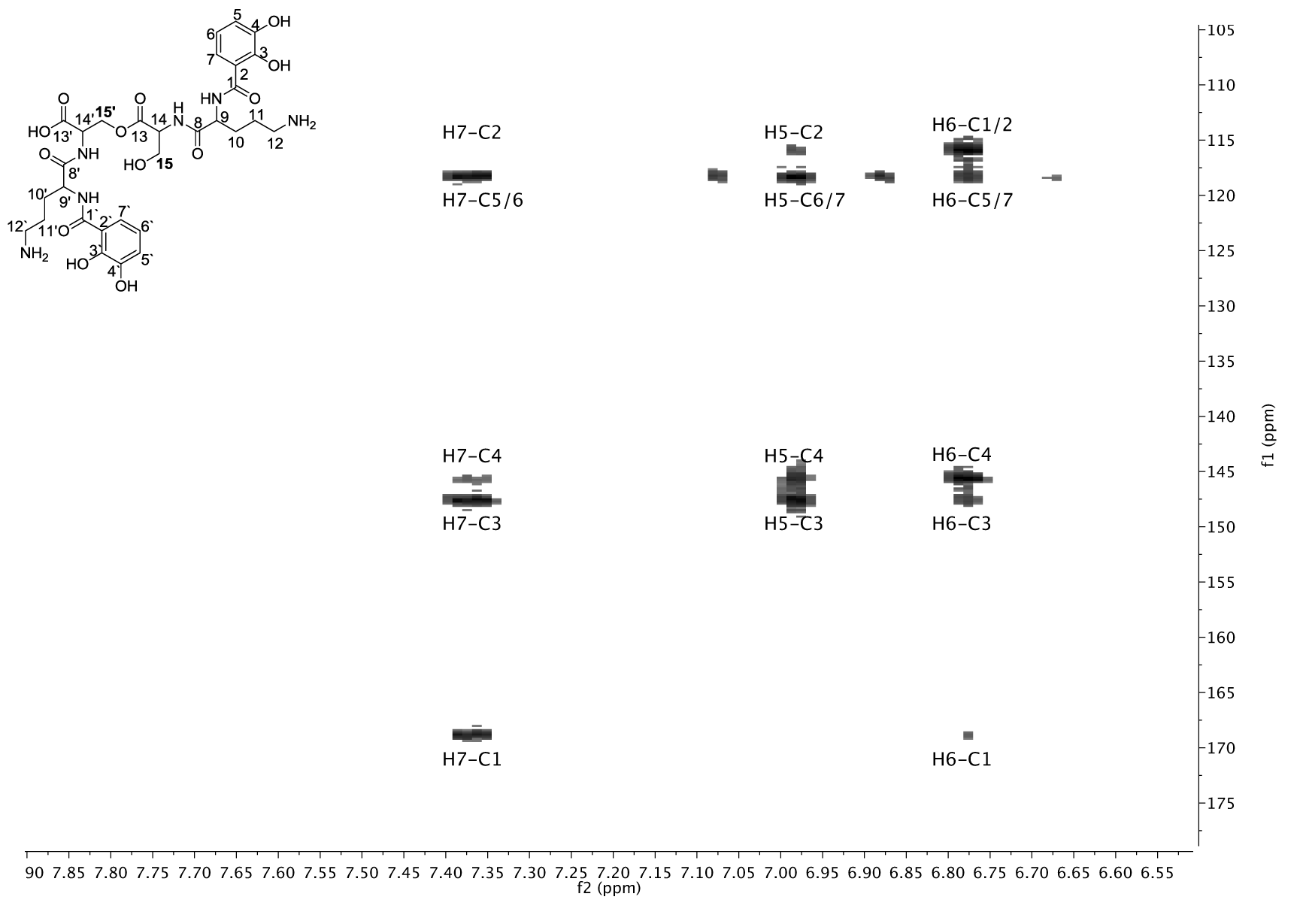

Supplement: Figure S18 — 2 1H-13C HMBC spectrum (800 MHz) in CD3OD, expanded region. (TIF) [file pone.0076151.s018.tif]

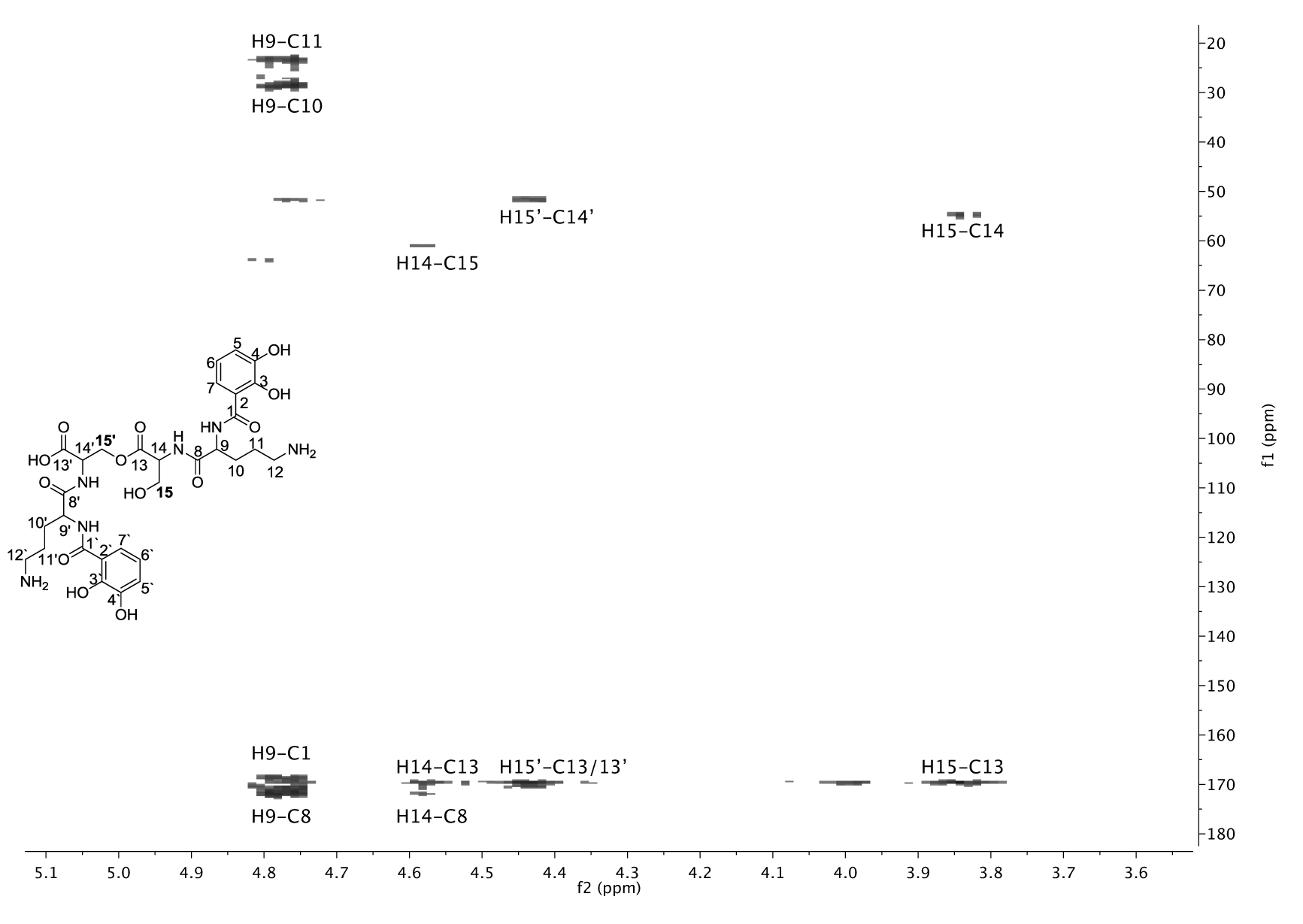

Supplement: Figure S19 — 2 1H-13C HMBC spectrum (800 MHz) in CD3OD, expanded region. (TIF) [file pone.0076151.s019.tif]

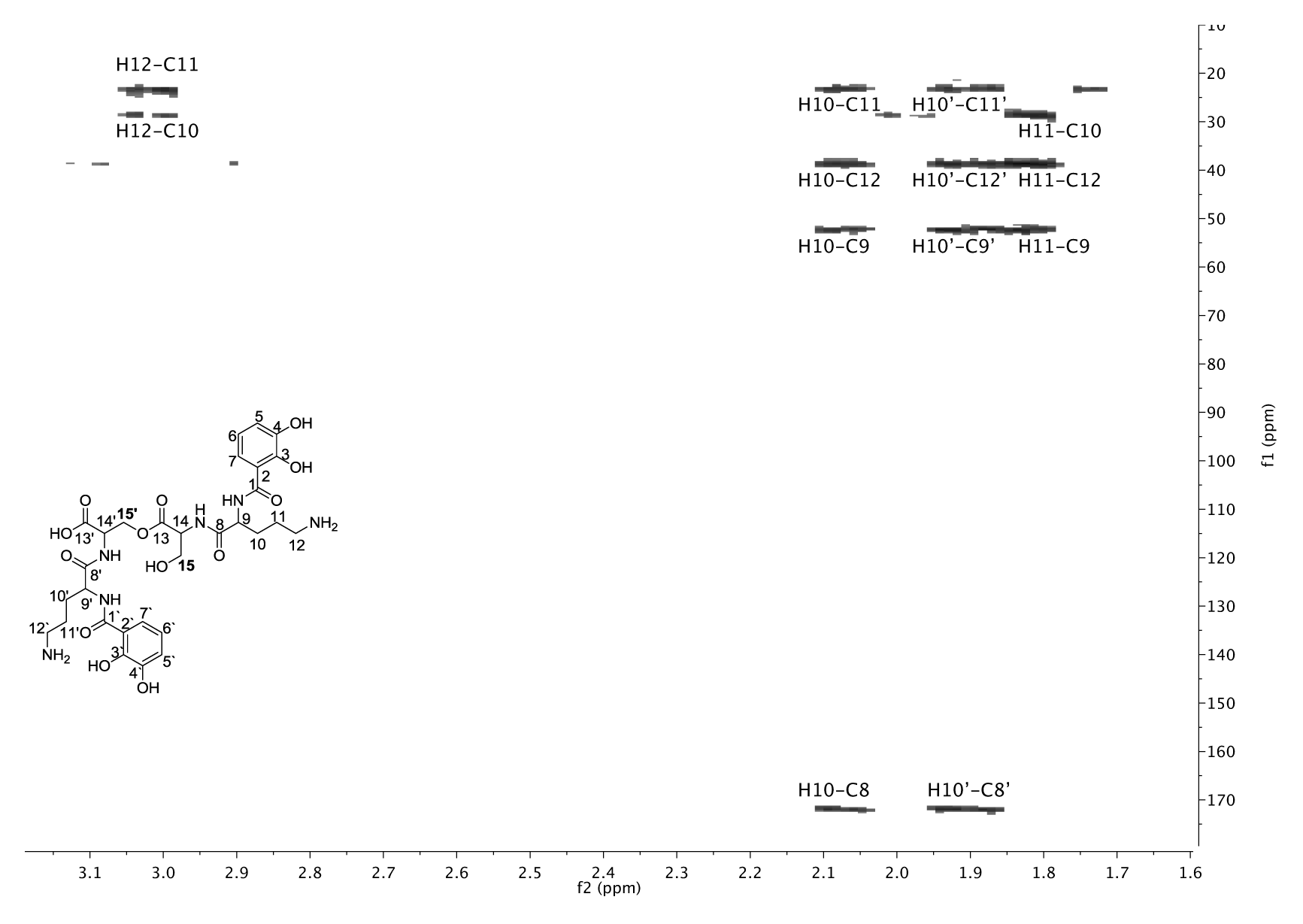

Supplement: Figure S20 — 2 1H-13C HMBC spectrum (800 MHz) in CD3OD, expanded region. (TIF) [file pone.0076151.s020.tif]

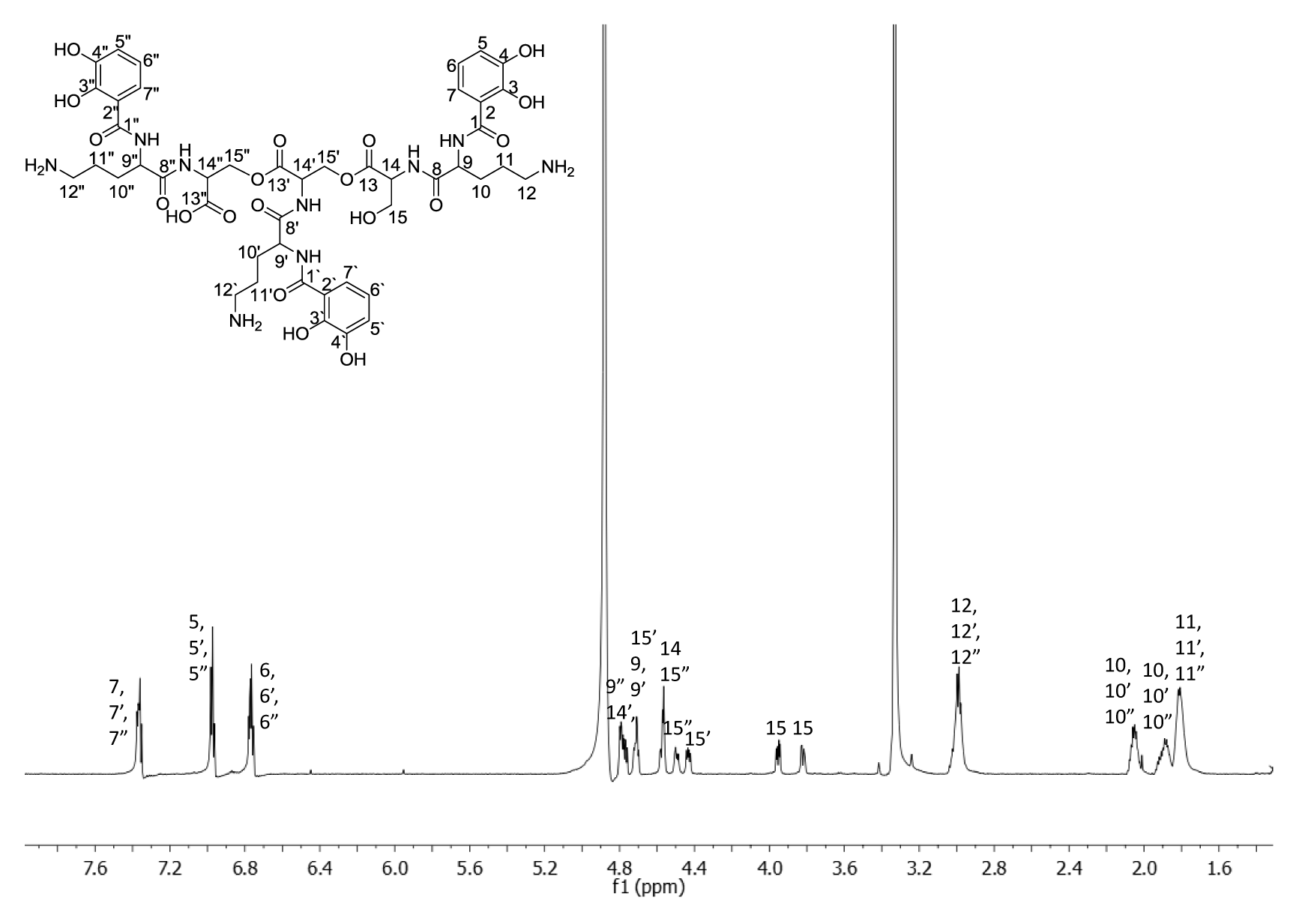

Supplement: Figure S21 — 3 1H NMR spectrum (800 MHz) in CD3OD. (TIF) [file pone.0076151.s021.tif]

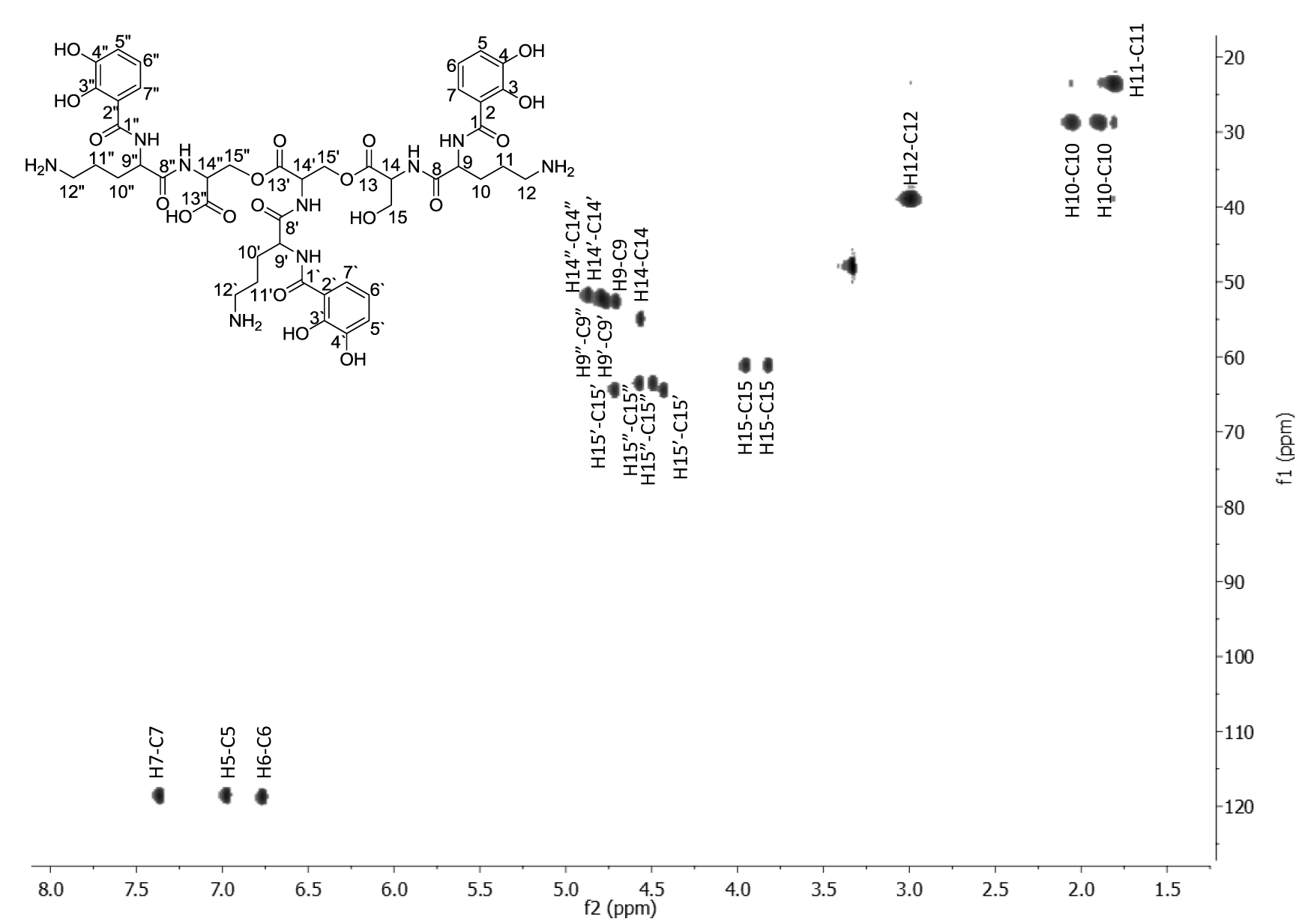

Supplement: Figure S22 — 3 1H-13C HSQC spectrum (800 MHz) in CD3OD. (TIF) [file pone.0076151.s022.tif]

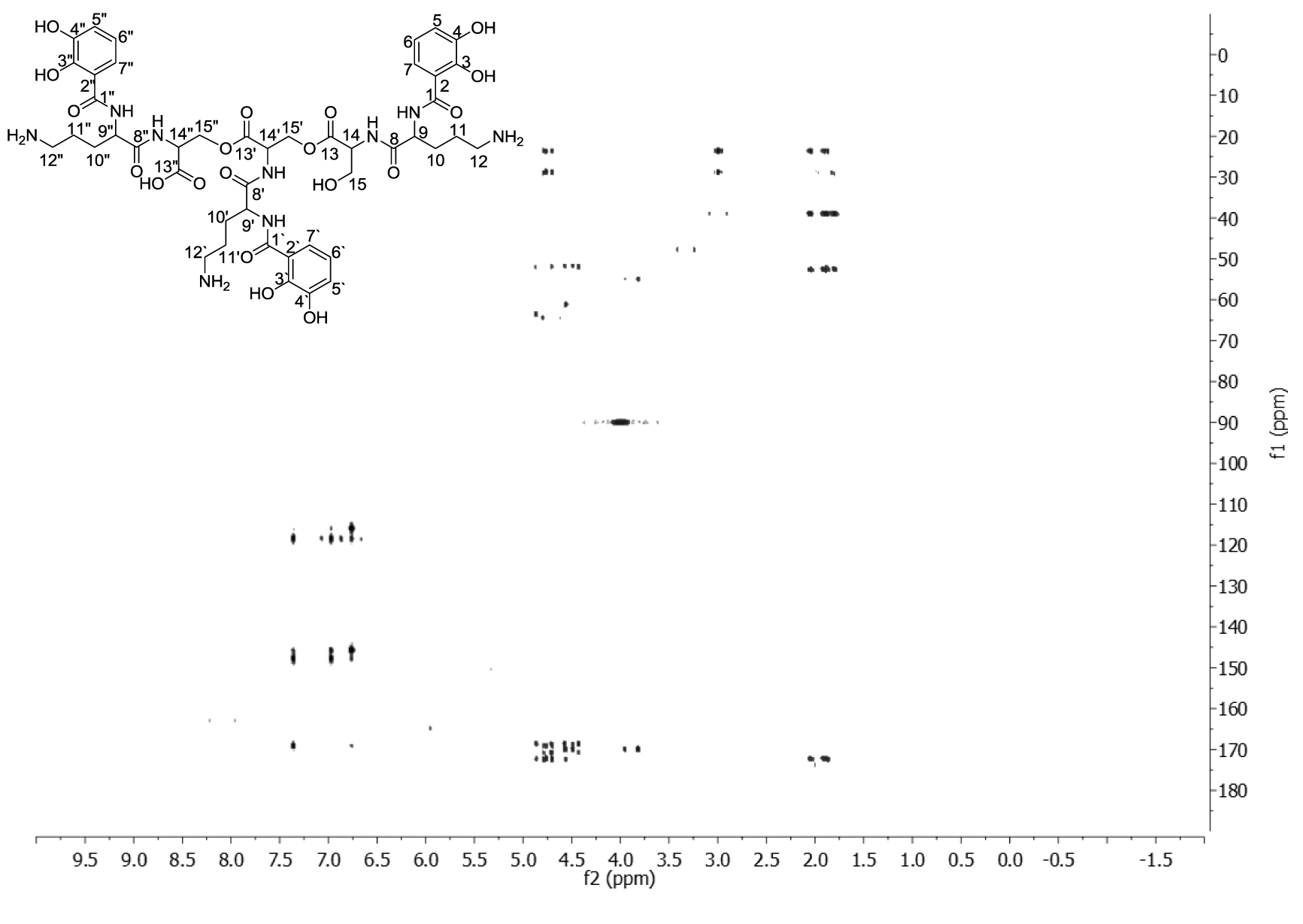

Supplement: Figure S23 — 3 1H-13C HMBC spectrum (800 MHz) in CD3OD. (TIF) [file pone.0076151.s023.tif]

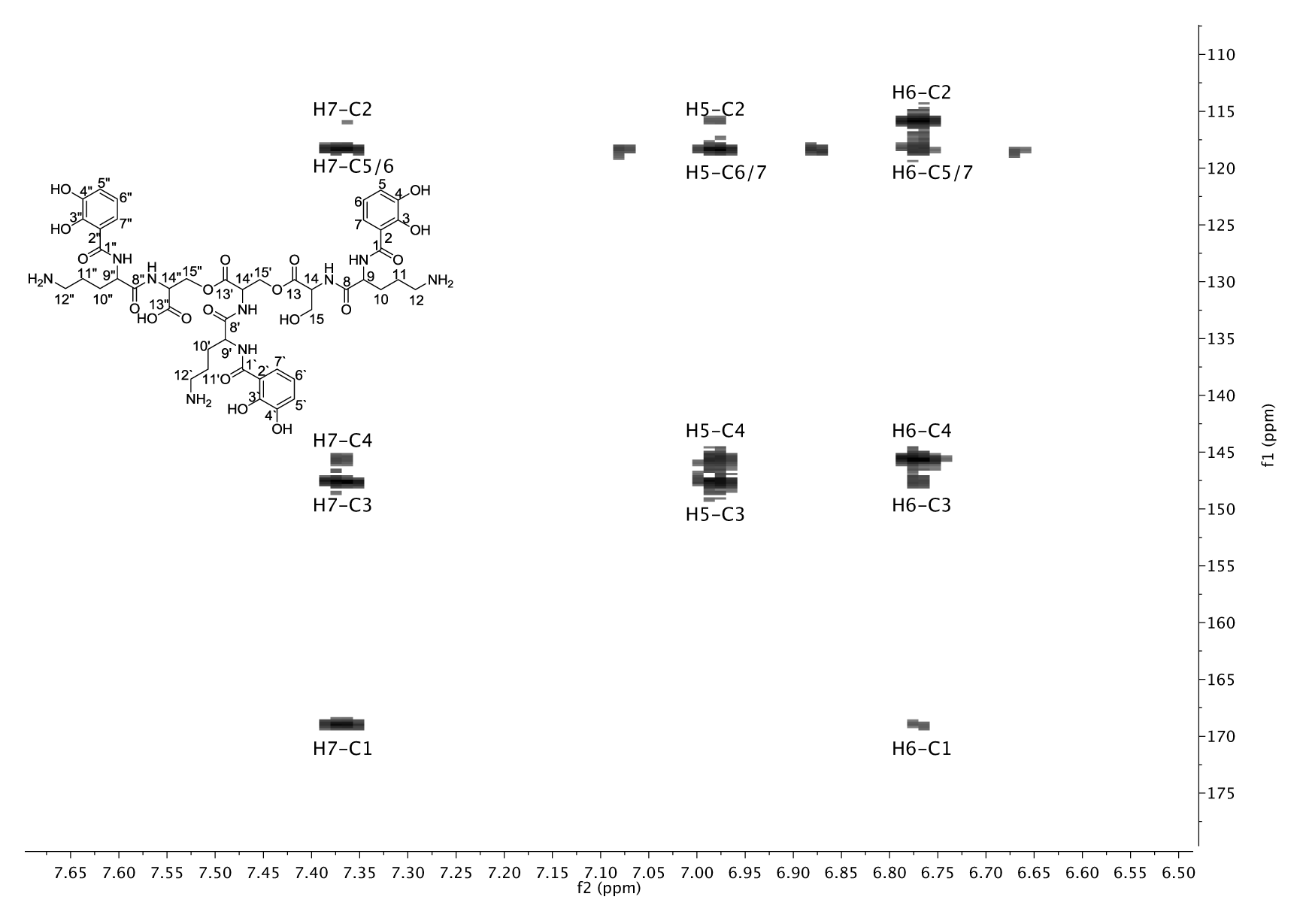

Supplement: Figure S24 — 3 1H-13C HMBC spectrum (800 MHz) in CD3OD, expanded region. (TIF) [file pone.0076151.s024.tif]

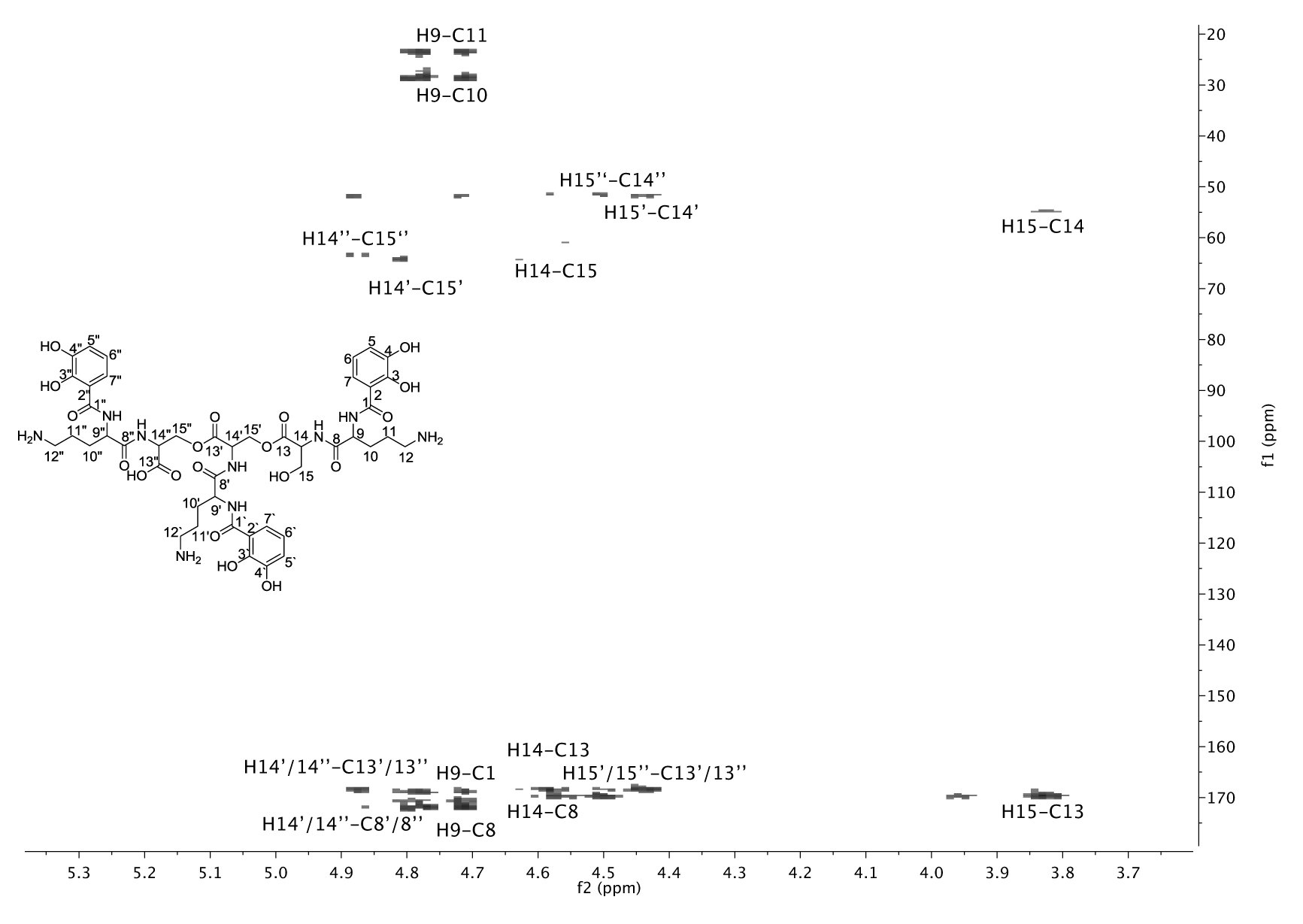

Supplement: Figure S25 — 3 1H-13C HMBC spectrum (800 MHz) in CD3OD, expanded region. (TIF) [file pone.0076151.s025.tif]

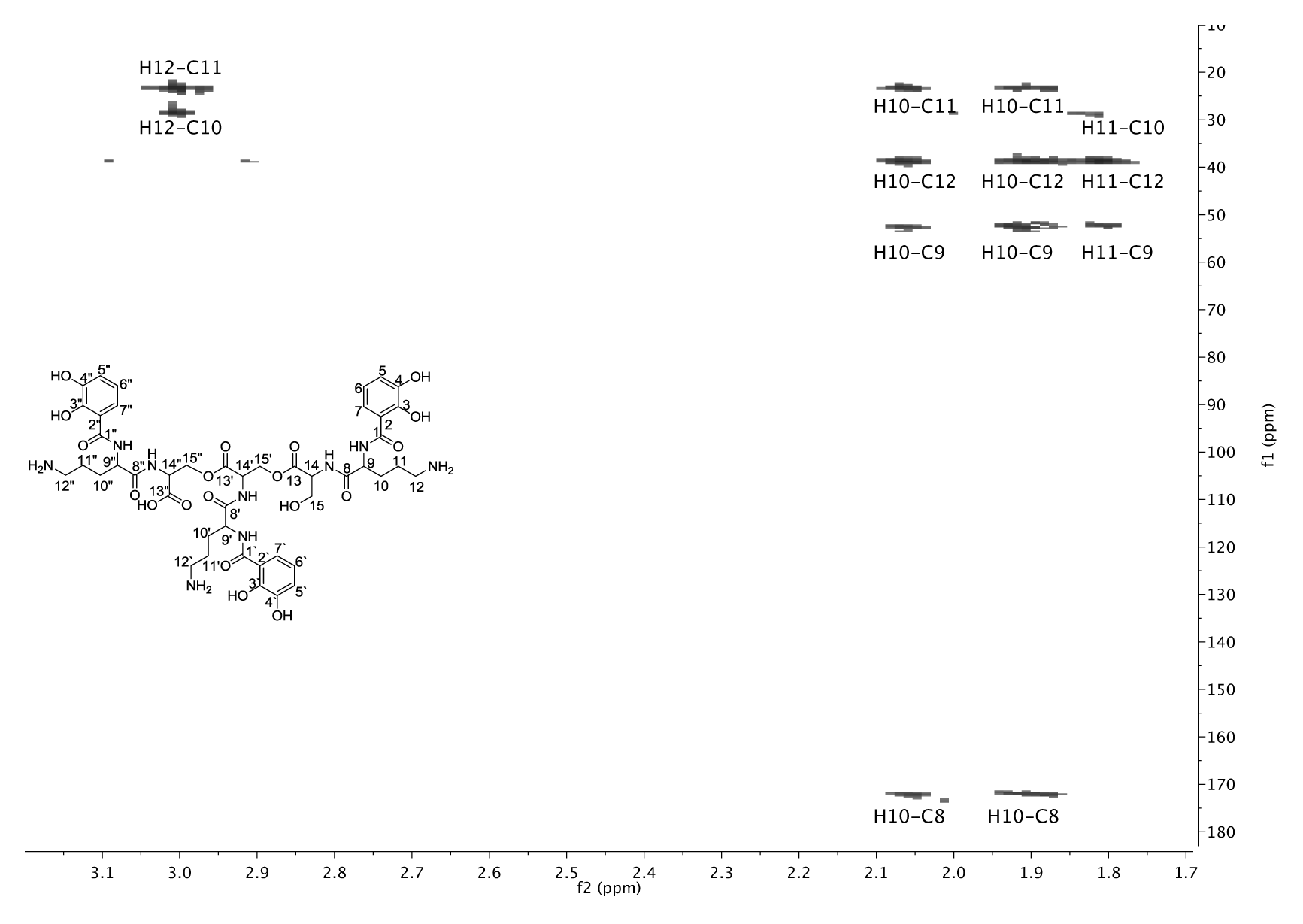

Supplement: Figure S26 — 3 1H-13C HMBC spectrum (800 MHz) in CD3OD, expanded region. (TIF) [file pone.0076151.s026.tif]

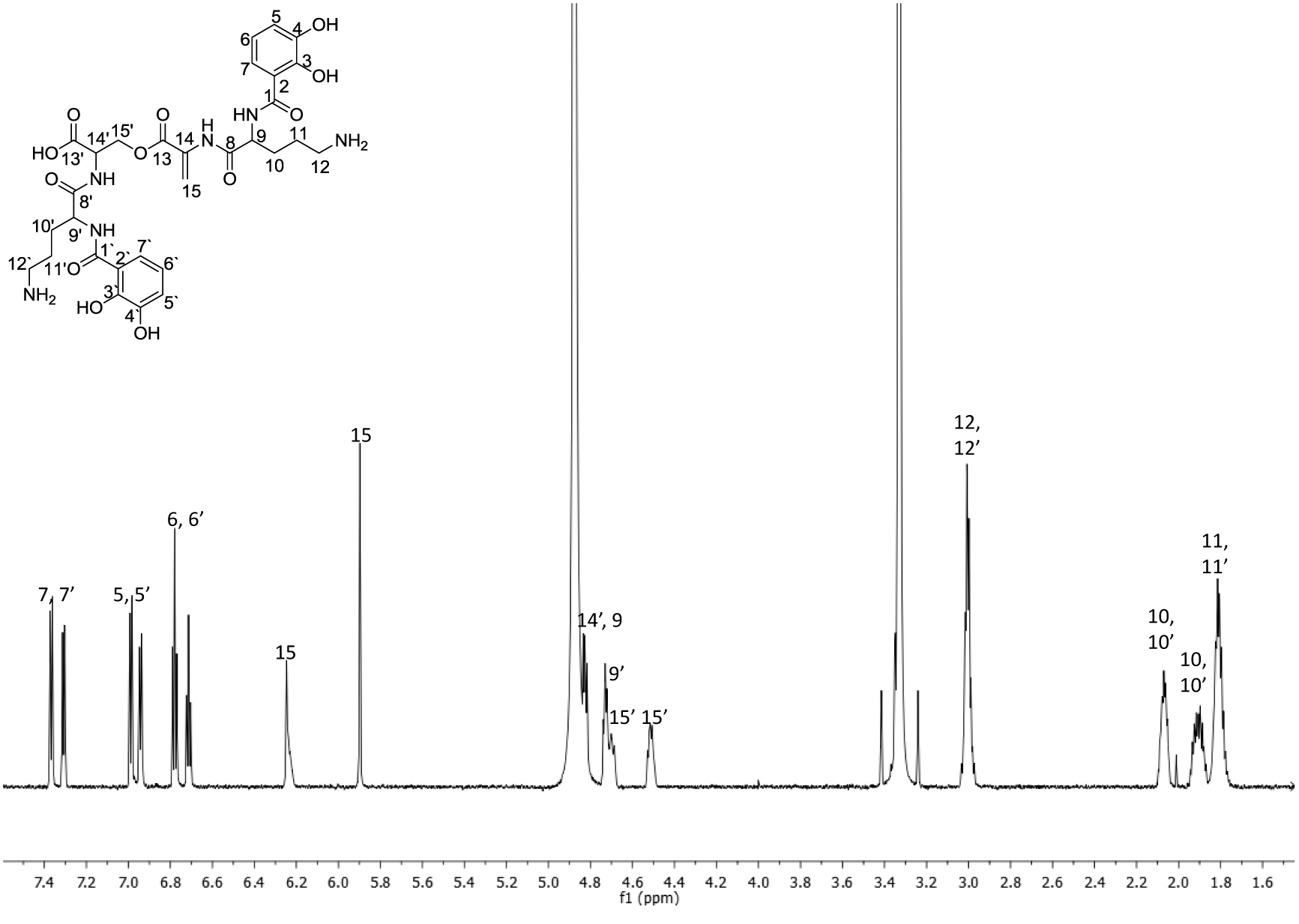

Supplement: Figure S27 — 4 1H NMR spectrum (800 MHz) in CD3OD. (TIF) [file pone.0076151.s027.tif]

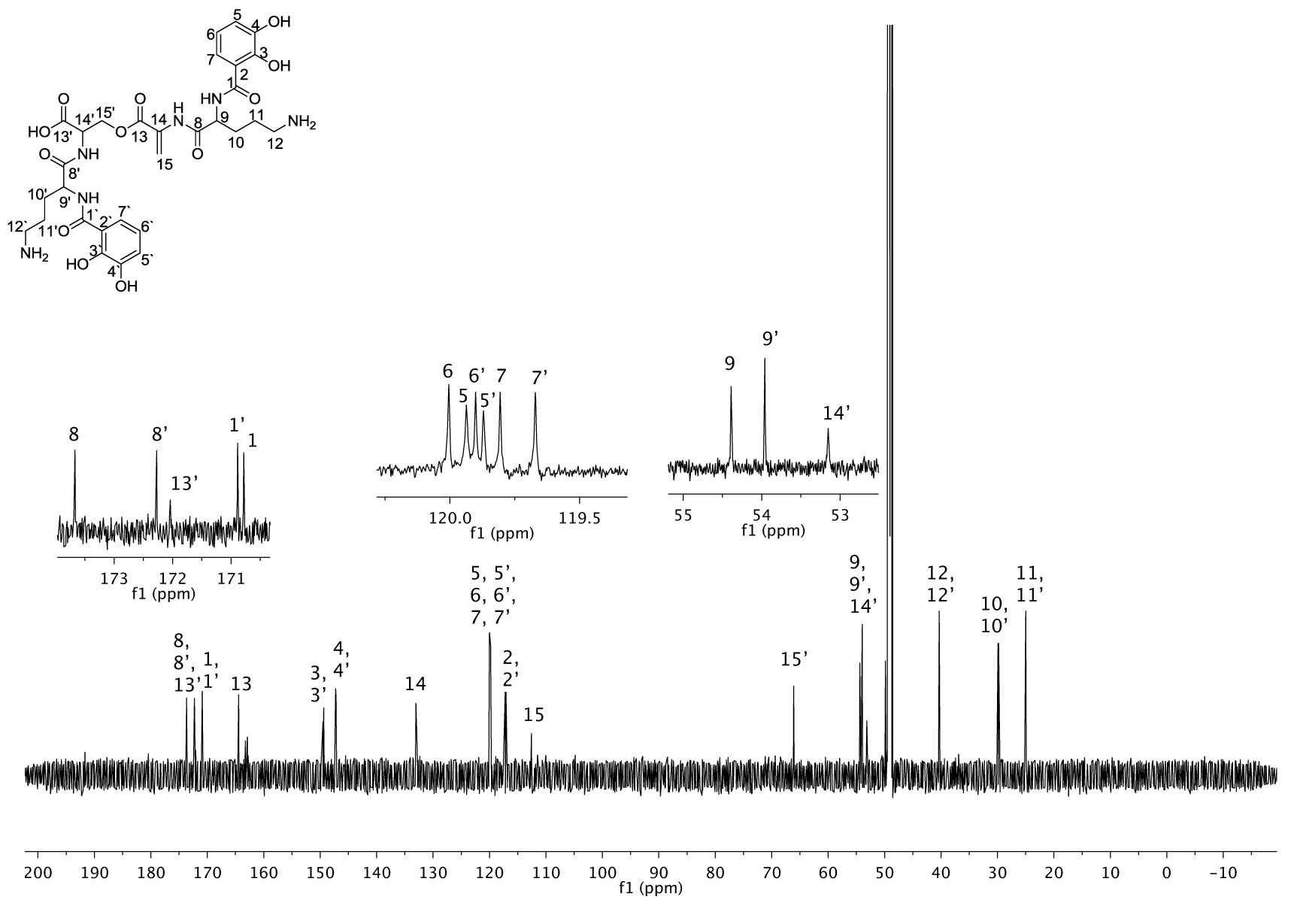

Supplement: Figure S28 — 4 13C NMR spectrum (800 MHz) in CD3OD. (TIF) [file pone.0076151.s028.tif]

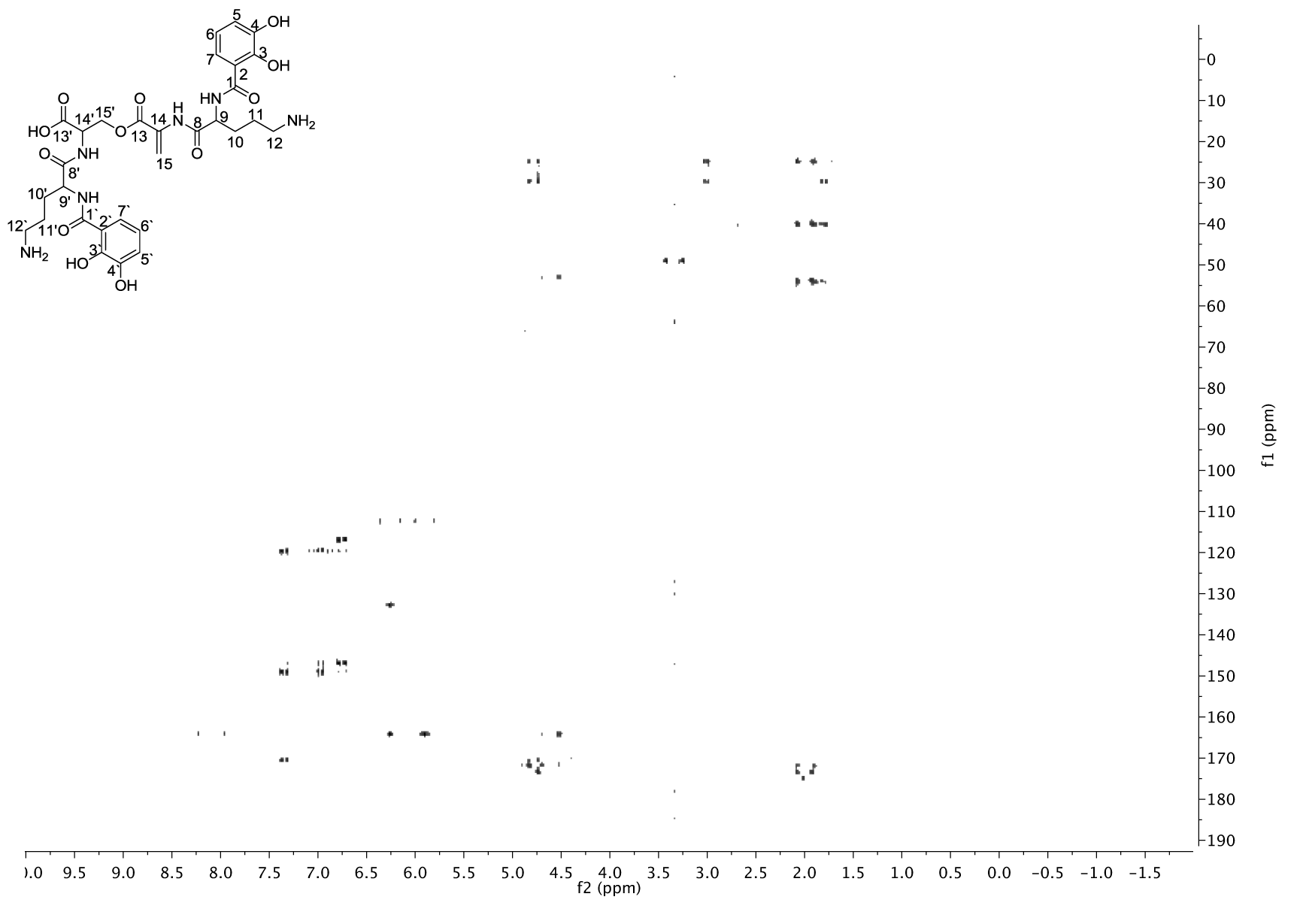

Supplement: Figure S29 — 4 1H-13C HMBC spectrum (800 MHz) in CD3OD. (TIF) [file pone.0076151.s029.tif]

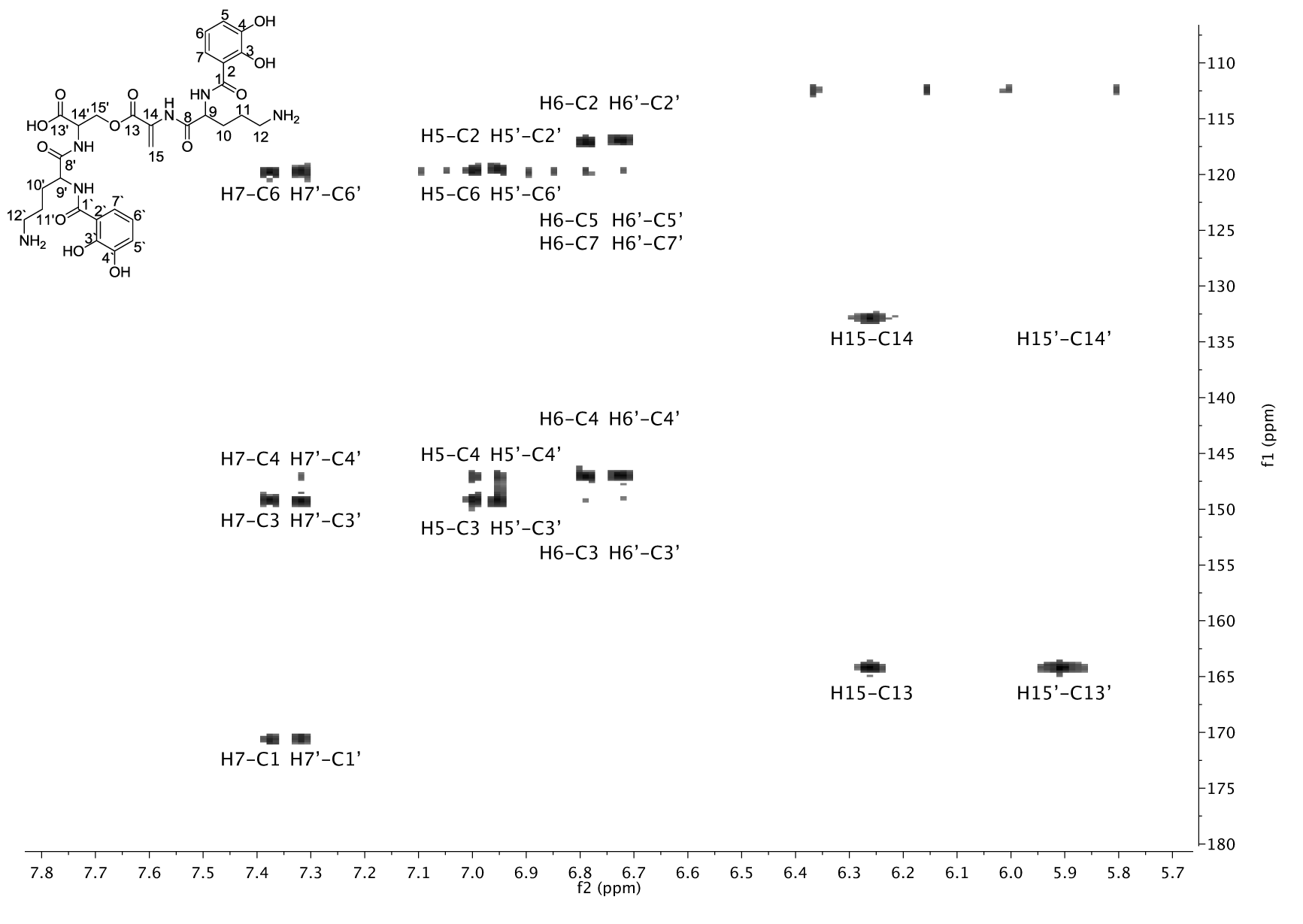

Supplement: Figure S30 — 4 1H-13C HMBC spectrum (800 MHz) in CD3OD, expanded region. (TIF) [file pone.0076151.s030.tif]

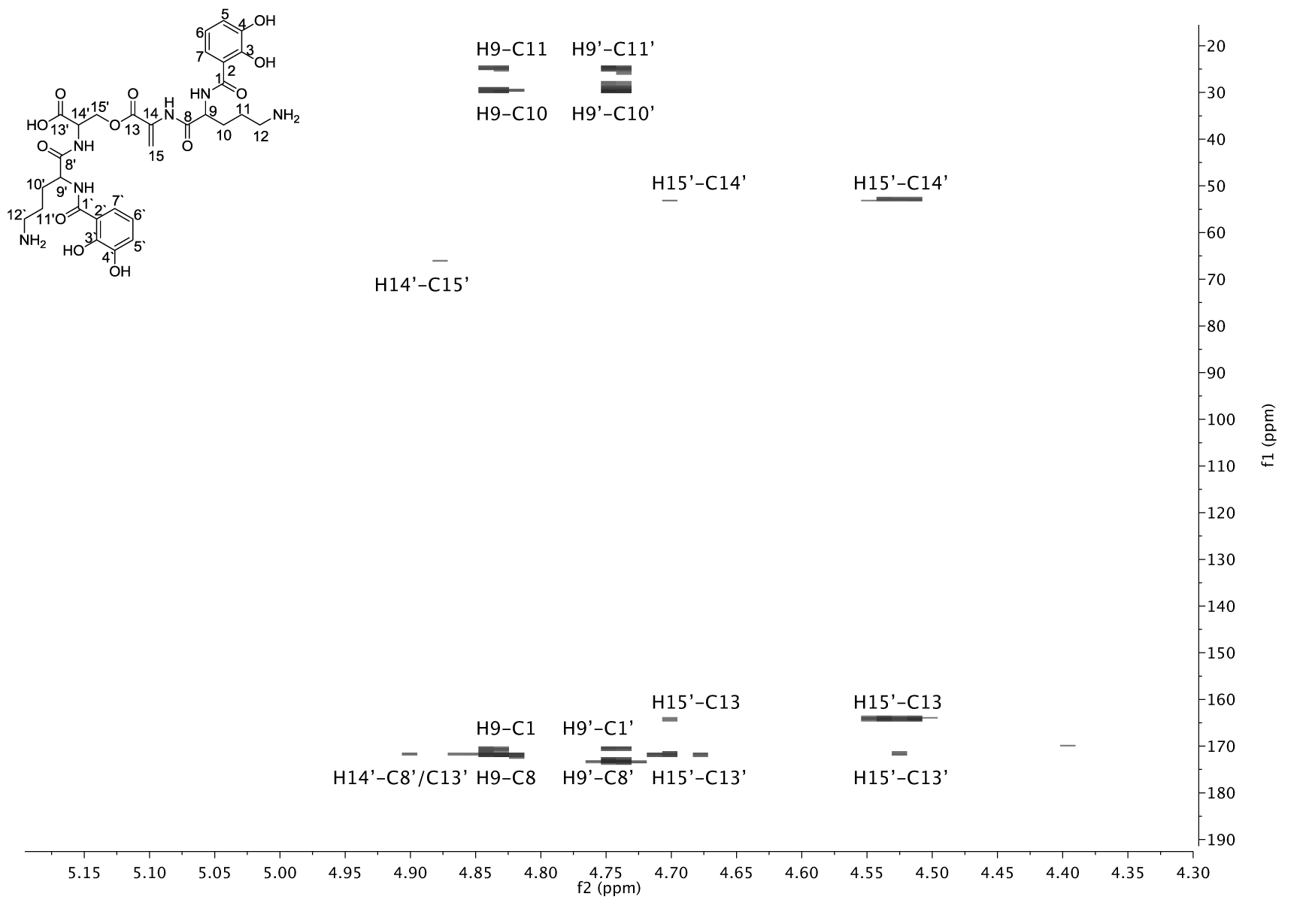

Supplement: Figure S31 — 4 1H-13C HMBC spectrum (800 MHz) in CD3OD, expanded region. (TIF) [file pone.0076151.s031.tif]

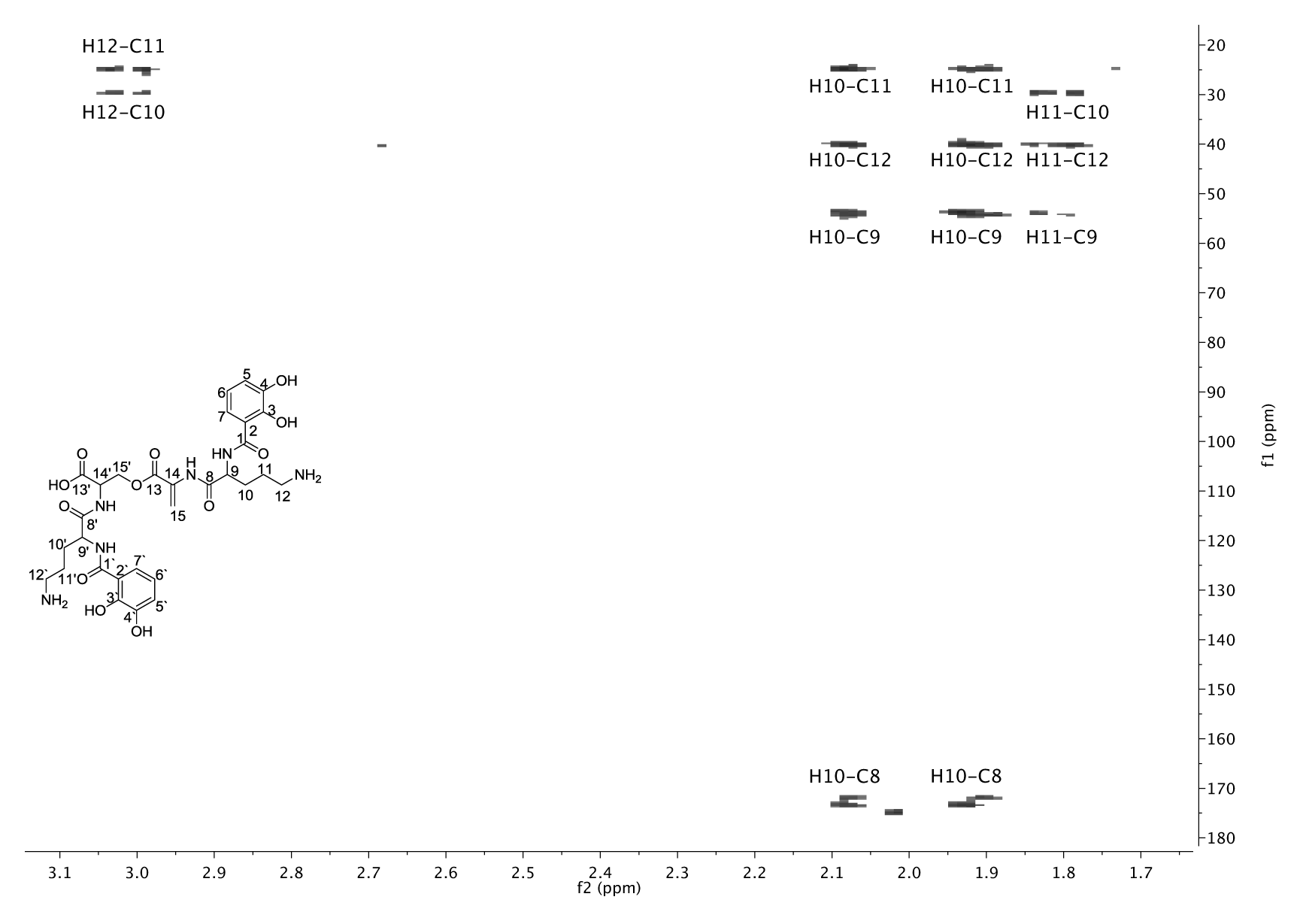

Supplement: Figure S32 — 4 1H-13C HMBC spectrum (800 MHz) in CD3OD, expanded region. (TIF) [file pone.0076151.s032.tif]

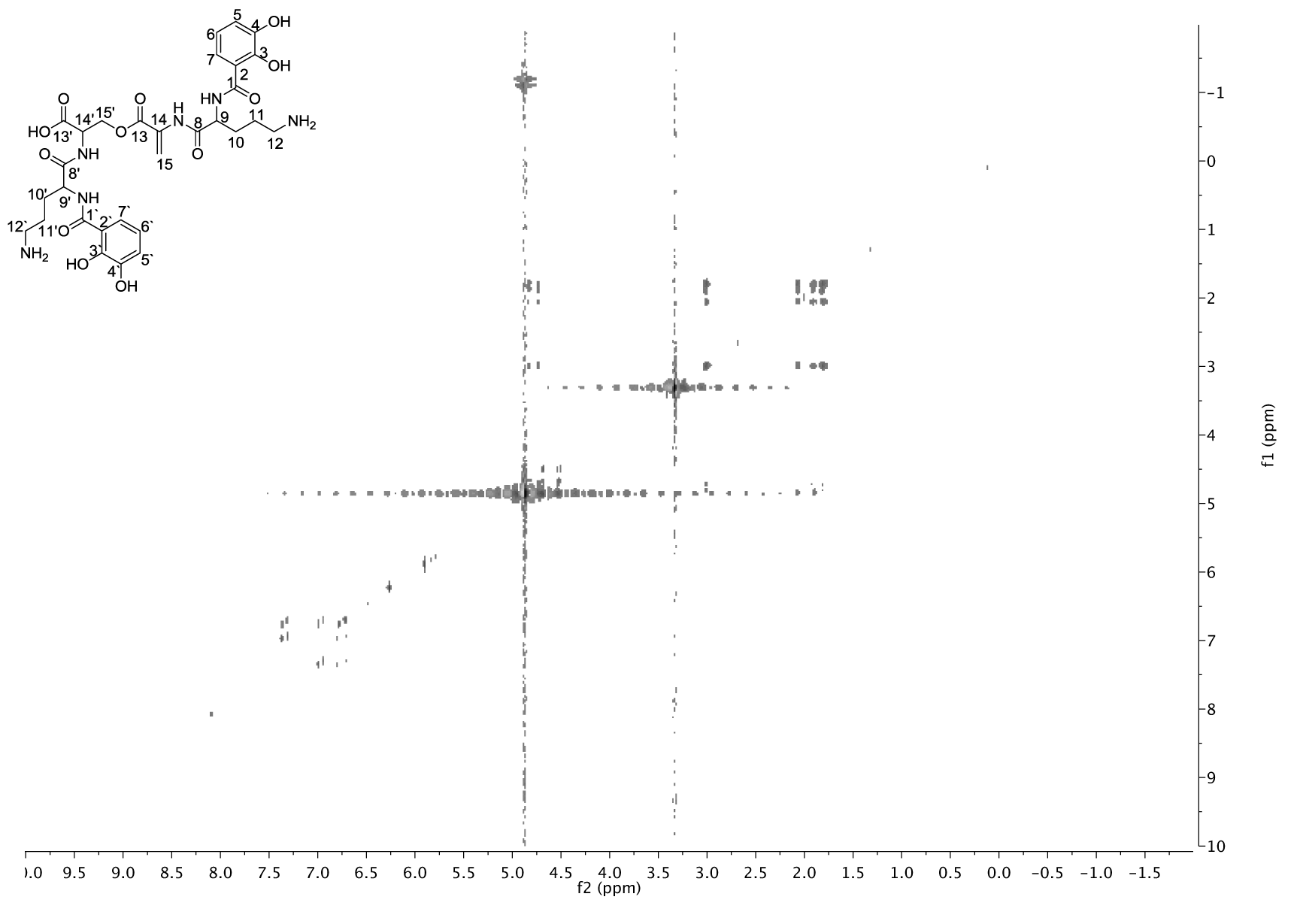

Supplement: Figure S33 — 4 1H-1H TOCSY spectrum (800 MHz) in CD3OD. (TIF) [file pone.0076151.s033.tif]

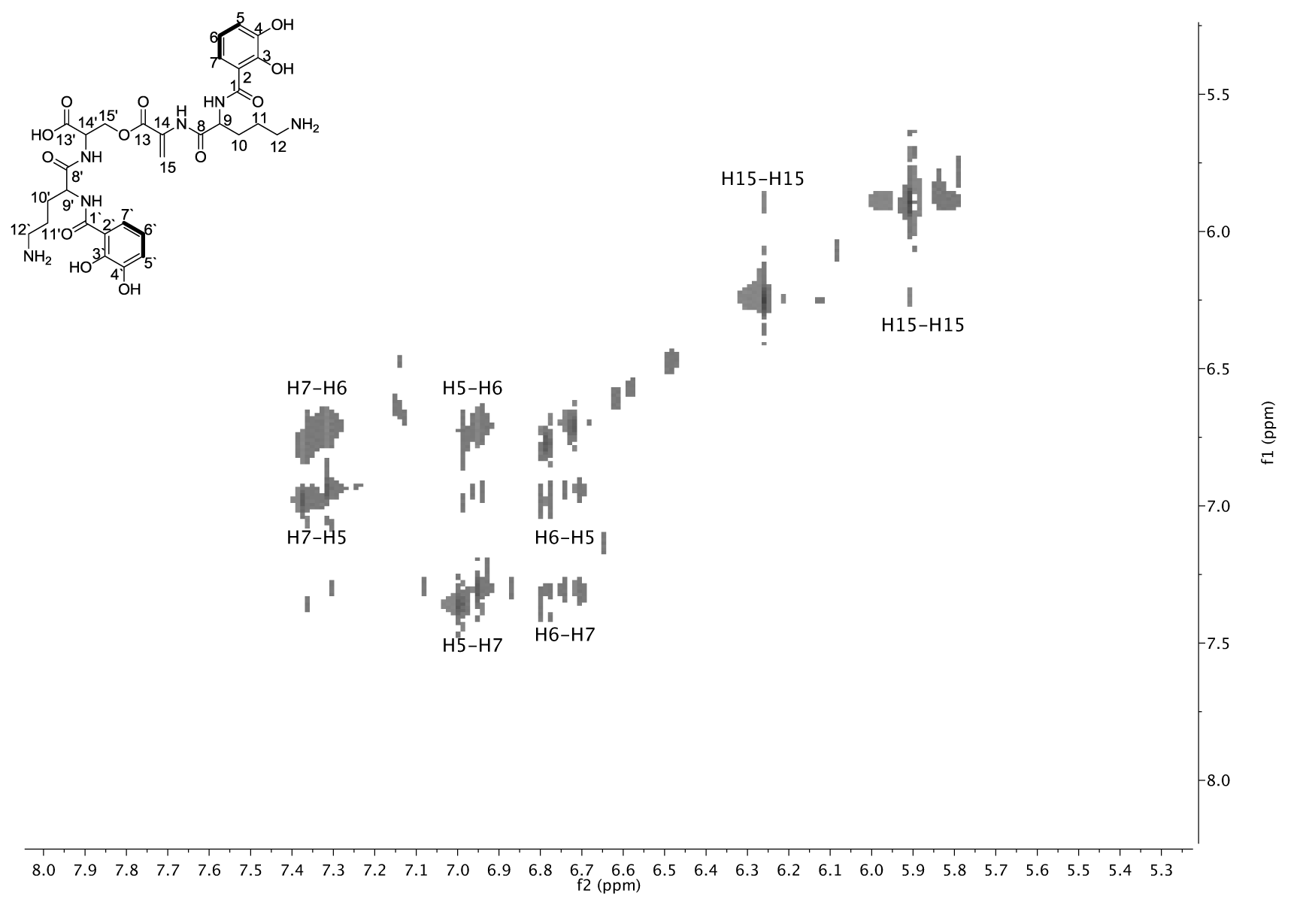

Supplement: Figure S34 — 4 1H-1H TOCSY spectrum (800 MHz) in CD3OD, expanded region. (TIF) [file pone.0076151.s034.tif]

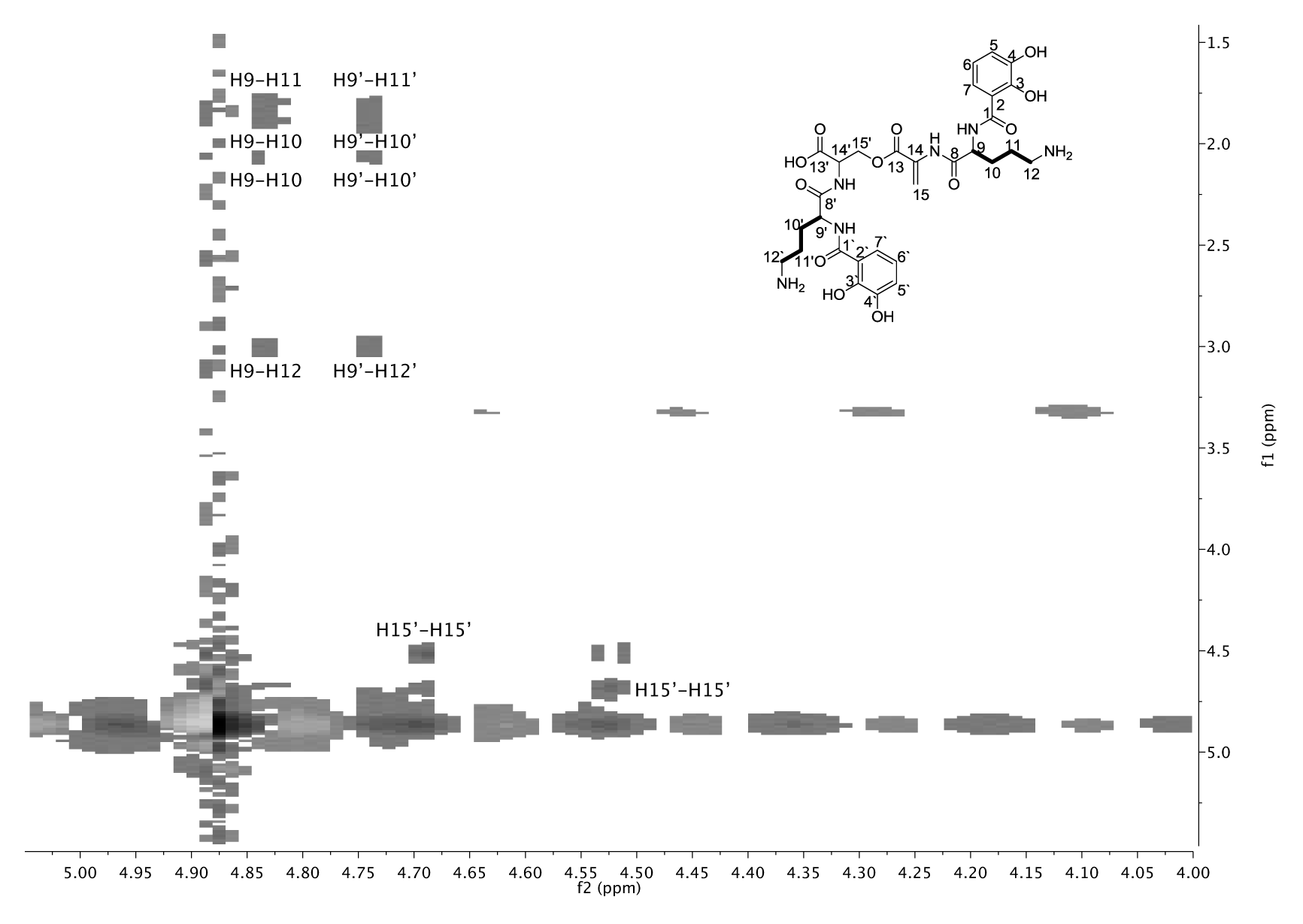

Supplement: Figure S35 — 4 1H-1H TOCSY spectrum (800 MHz) in CD3OD, expanded region. (TIF) [file pone.0076151.s035.tif]

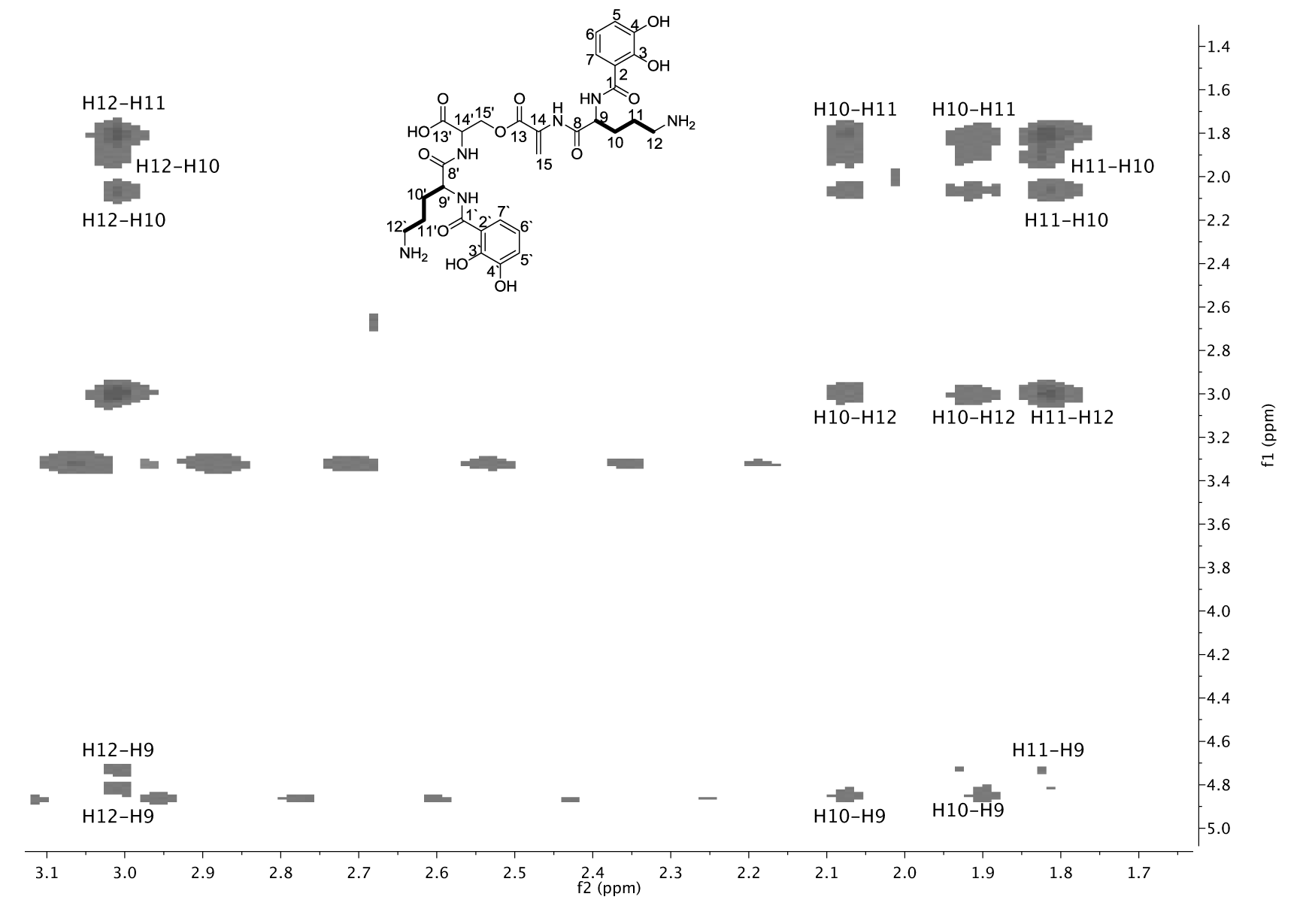

Supplement: Figure S36 — 4 1H-1H TOCSY spectrum (800 MHz) in CD3OD, expanded region. (TIF) [file pone.0076151.s036.tif]

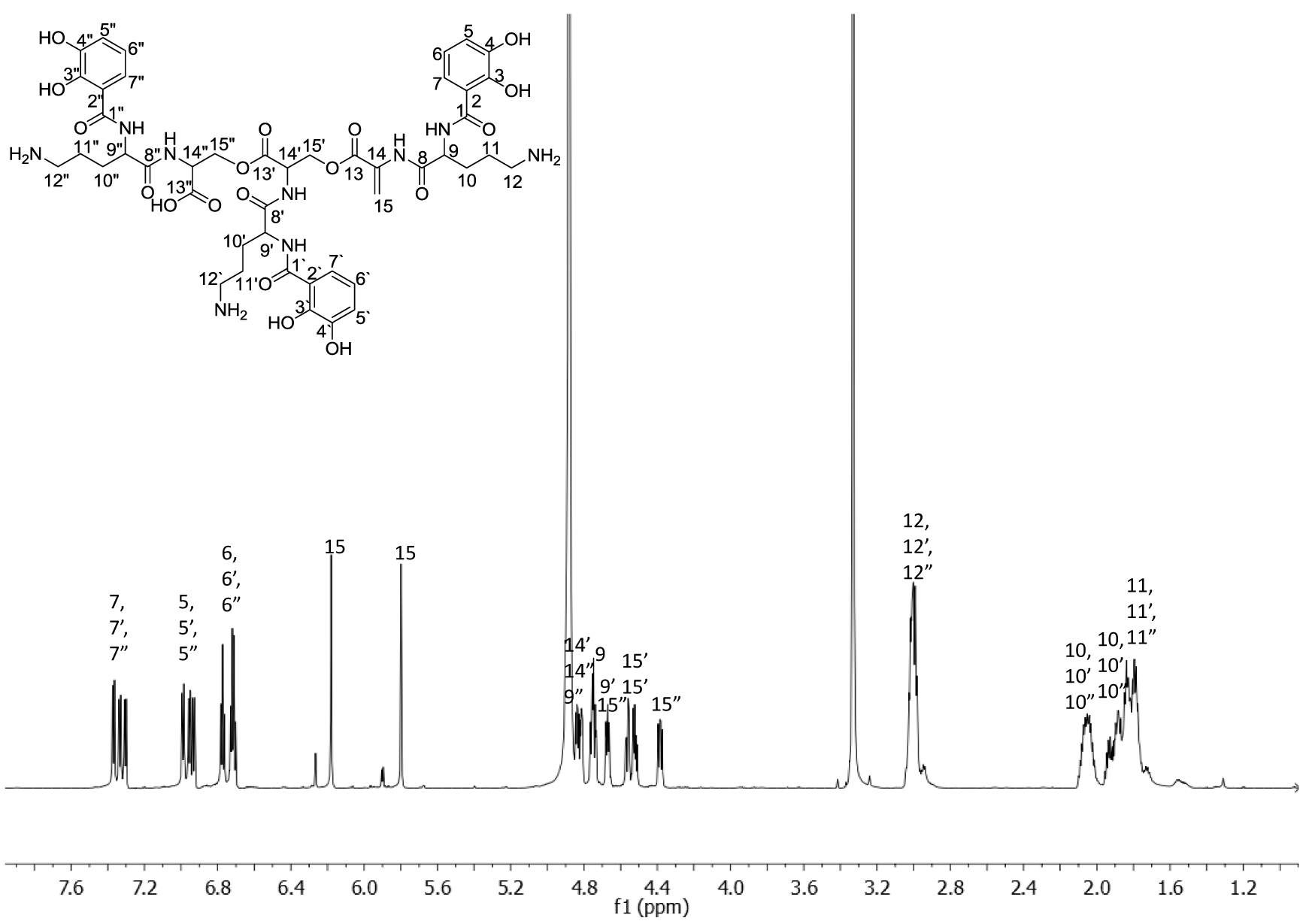

Supplement: Figure S37 — 5 1H NMR spectrum (800 MHz) in CD3OD. (TIF) [file pone.0076151.s037.tif]

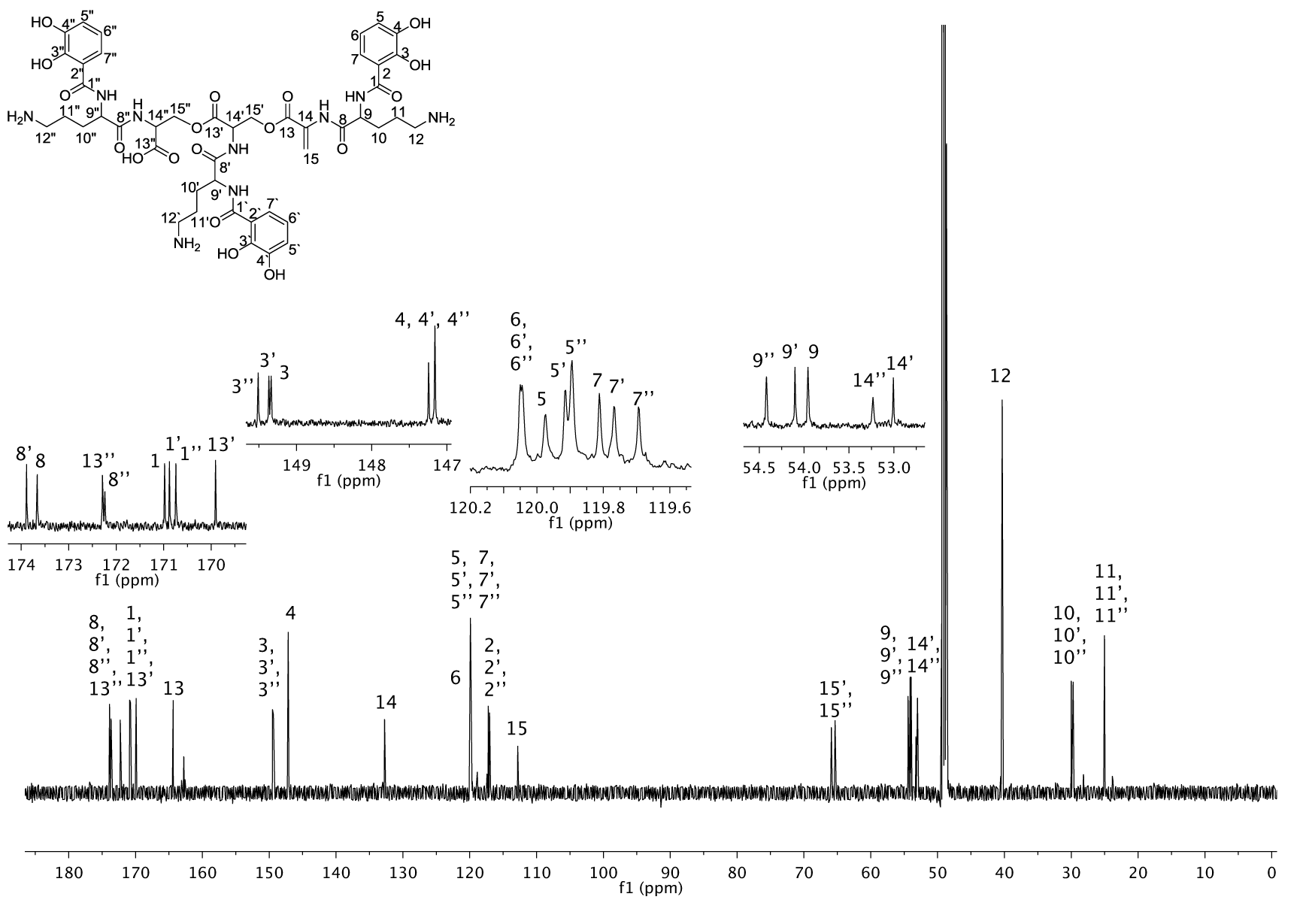

Supplement: Figure S38 — 5 13C NMR spectrum (800 MHz) in CD3OD. (TIF) [file pone.0076151.s038.tif]

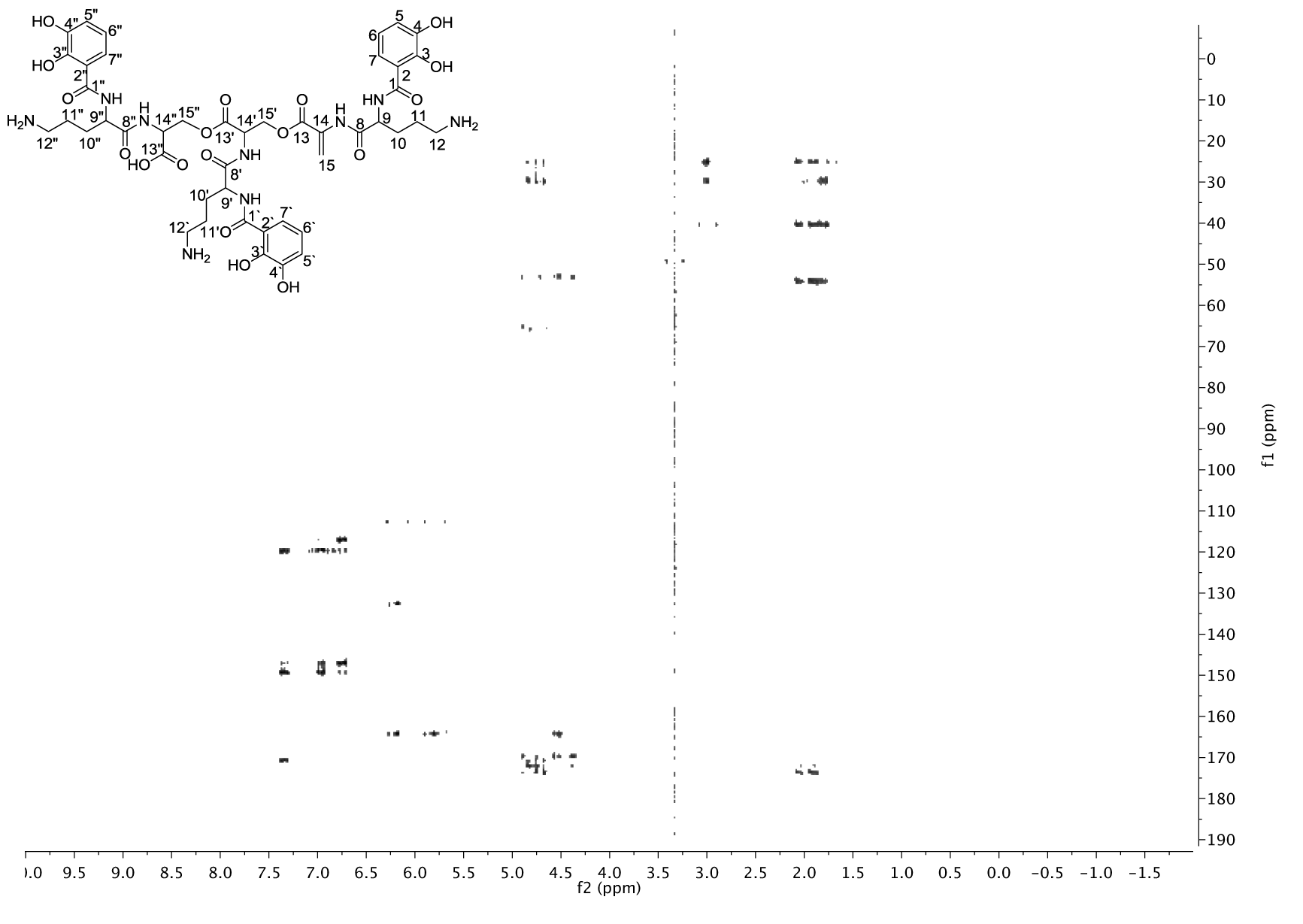

Supplement: Figure S39 — 5 1H-13C HMBC spectrum (800 MHz) in CD3OD. (TIF) [file pone.0076151.s039.tif]

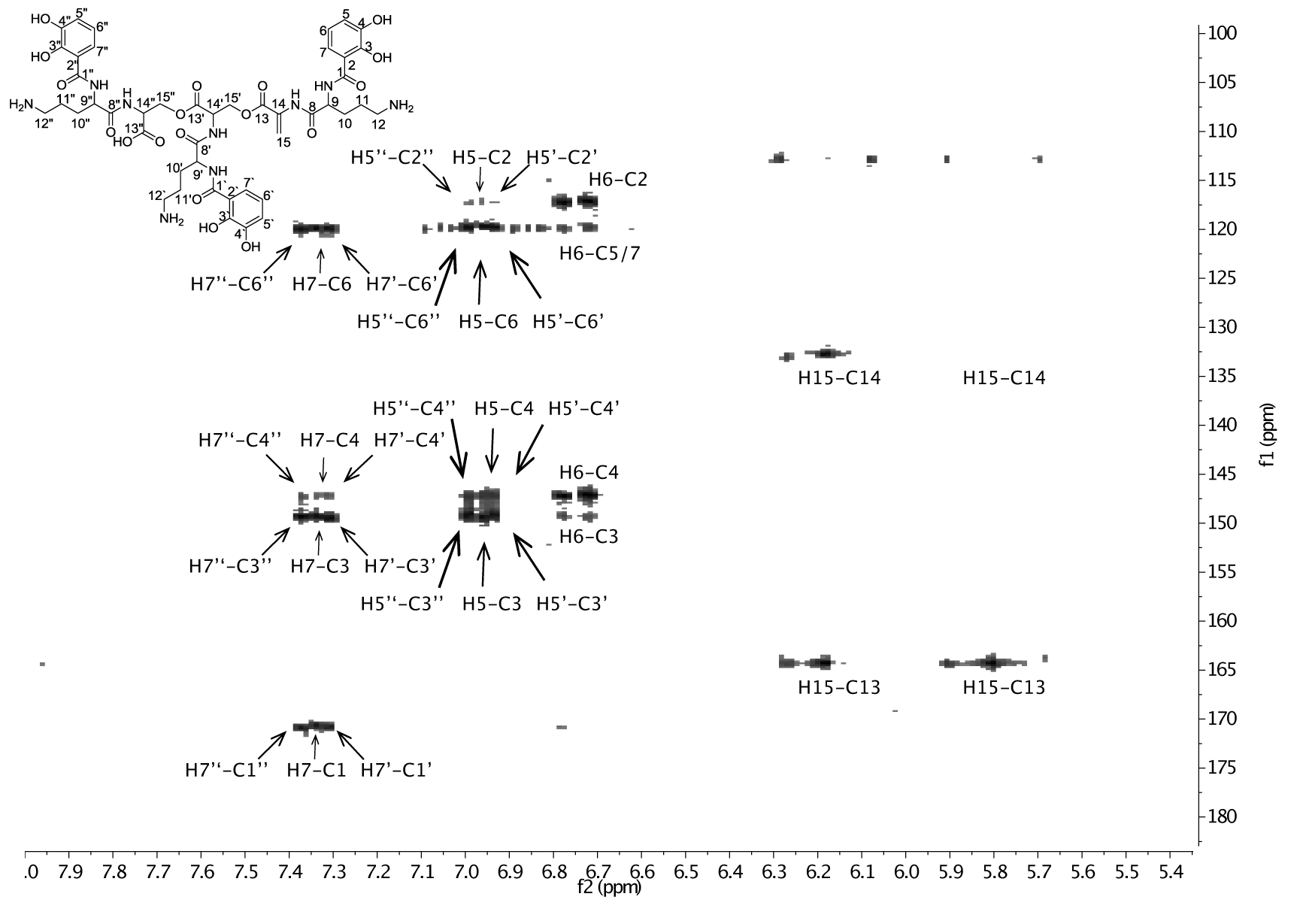

Supplement: Figure S40 — 5 1H-13C HMBC spectrum (800 MHz) in CD3OD, expanded region. (TIF) [file pone.0076151.s040.tif]

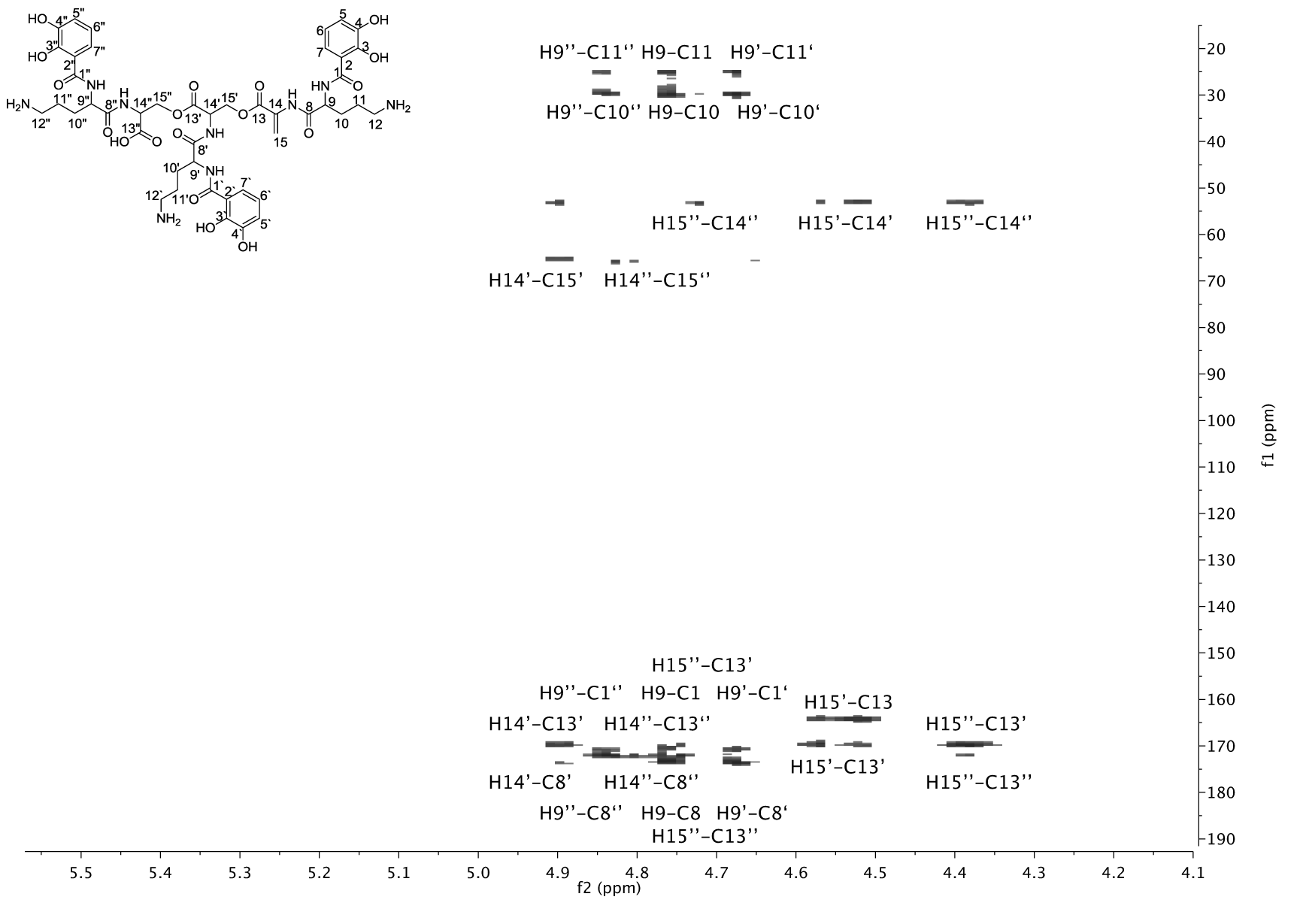

Supplement: Figure S41 — 5 1H-13C HMBC spectrum (800 MHz) in CD3OD, expanded region. (TIF) [file pone.0076151.s041.tif]

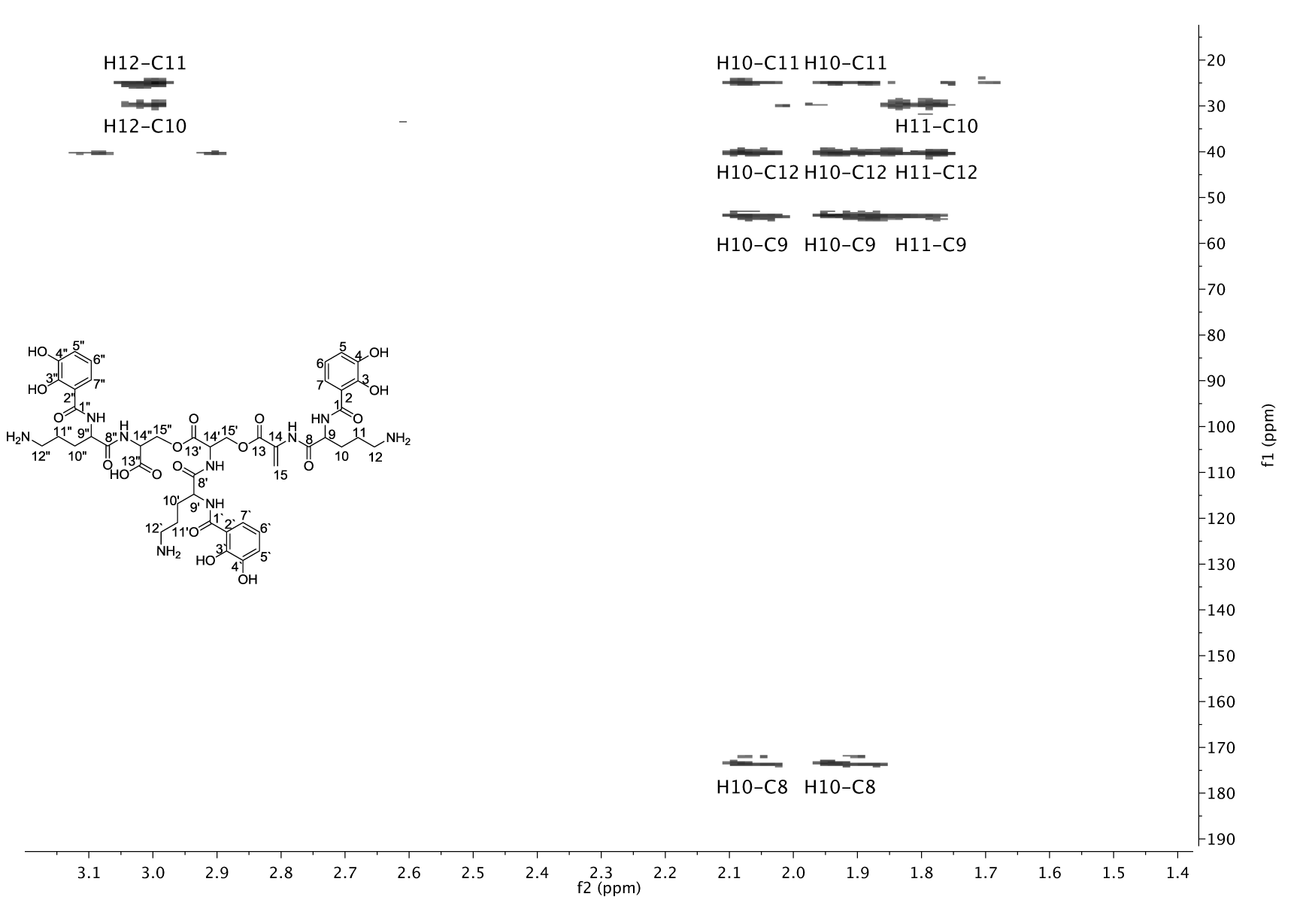

Supplement: Figure S42 — 5 1H-13C HMBC spectrum (800 MHz) in CD3OD, expanded region. (TIF) [file pone.0076151.s042.tif]

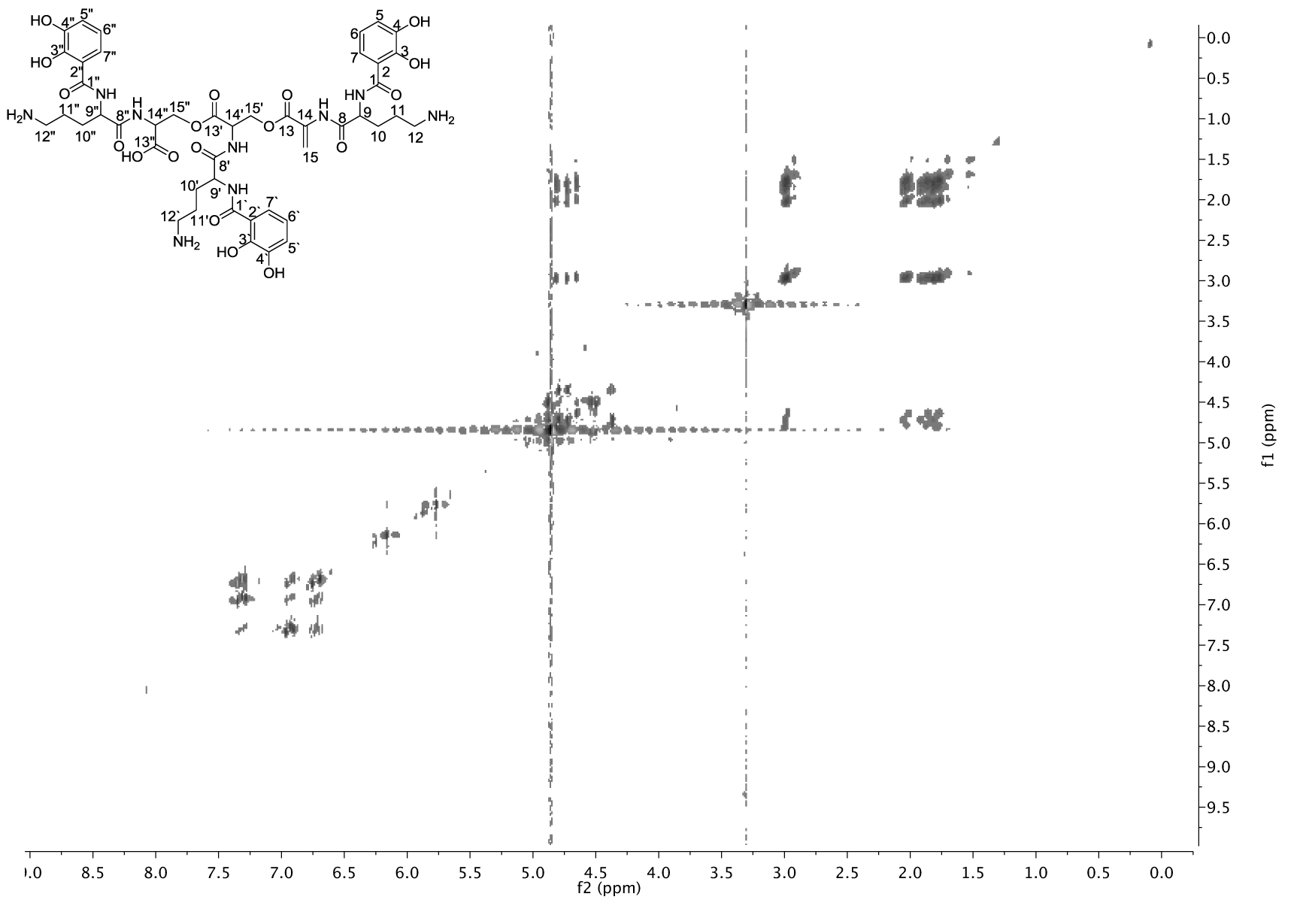

Supplement: Figure S43 — 5 1H-1H TOCSY spectrum (800 MHz) in CD3OD. (TIF) [file pone.0076151.s043.tif]

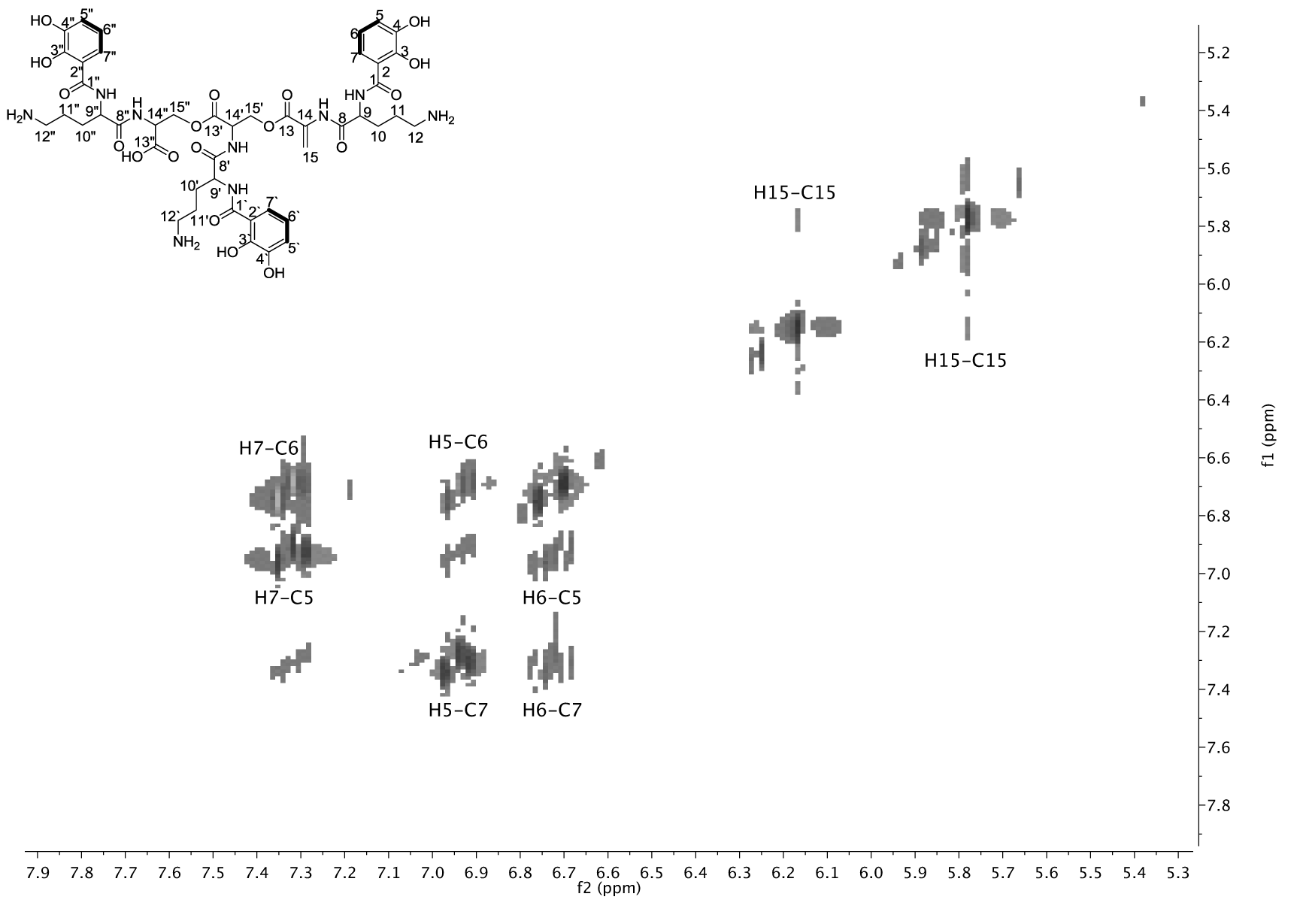

Supplement: Figure S44 — 5 1H-1H TOCSY spectrum (800 MHz) in CD3OD, expanded region. (TIF) [file pone.0076151.s044.tif]

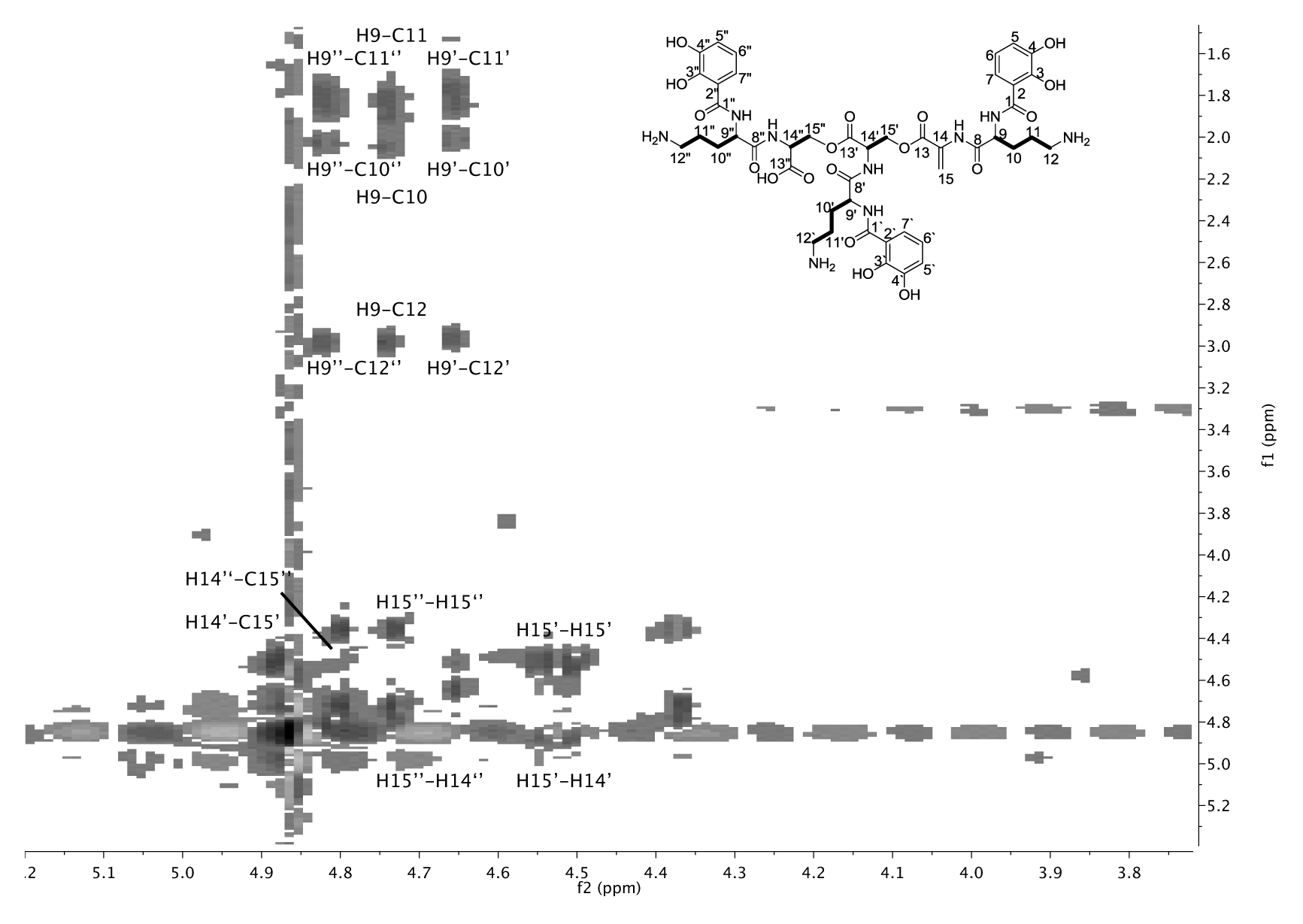

Supplement: Figure S45 — 5 1H-1H TOCSY spectrum (800 MHz) in CD3OD, expanded region. (TIF) [file pone.0076151.s045.tif]

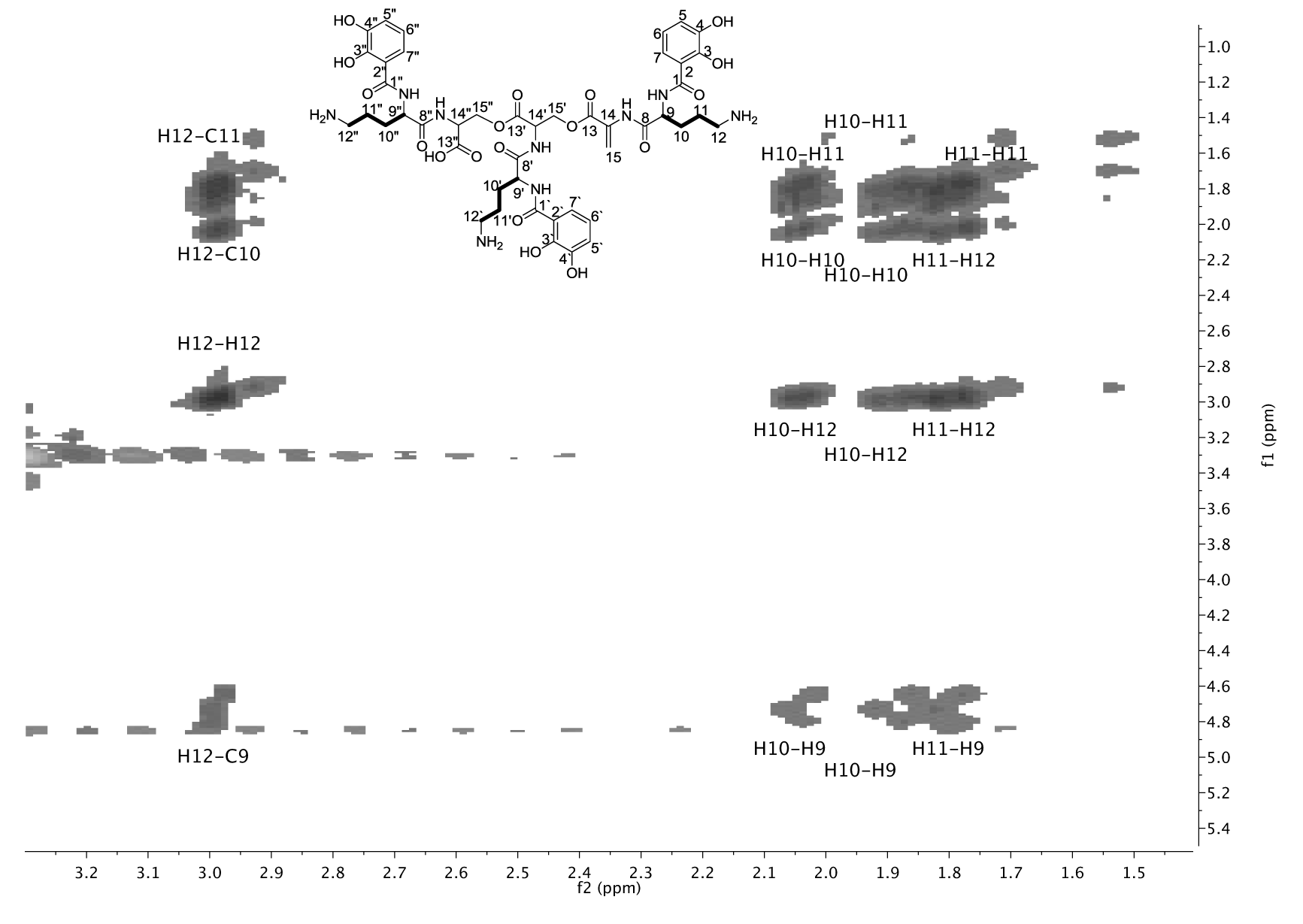

Supplement: Figure S46 — 5 1H-1H TOCSY spectrum (800 MHz) in CD3OD, expanded region. (TIF) [file pone.0076151.s046.tif]
