# Supplementary material for: Turnerbactin, a Novel Triscatecholate Siderophore from the Shipworm Endosymbiont Teredinibacter turnerae T7901
Source: PLoS One. 2013 Oct 11;8(10):e76151. doi: 10.1371/journal.pone.0076151 (PMC3795760; doi:10.1371/journal.pone.0076151)
Supplement: Table S1 — NMR data for (DHB-Orn-Ser)2 (2), Dehydrated (DHB-Orn-Ser)2 (4), and Dehydrated (DHB-Orn-Ser)3 (5) (800 MHz) in CD3OD. (PDF) [file pone.0076151.s047.pdf]

Supporting Information

Table S1.

	(DHB-Orn-Ser)$_2$ (**2**)			Dehydrated (DHB-Orn-Ser)$_2$ (**4**)				Dehydrated (DHB-Orn-Ser)$_3$ (**5**)			
Position	δH (J in Hz)	HSQC	HMBC	δC	δH (J in Hz)	TOCSY	HMBC	δC	δH (J in Hz)	TOCSY	HMBC
DHB											
1, 1', 1''			169	170.78; 170.88				170.74; 170.88; 170.98			
2, 2', 2''			116	117.21; 117.06				117.21; 117.16; 117.01			
3, 3', 3''			147.7	149.49; 149.37				149.33; 149.36; 149.50			
4, 4', 4''			145.7	147.26; 147.17				147.24; 147.15			
5, 5', 5''	6.98, dd (1.6, 2.4), [1H]; 6.97, dd (1.6, 2.4), [1H]	118.6	2, 3, 4, 6, 7	119.93; 119.86	6.99, dd (8.0, 1.6), [1H]; 6.95, dd (8.0, 1.6), [1H]	6, 7	2, 3, 4, 6; 2', 3', 4', 6'	119.97; 119.91; 119.90	6.95, dd (1.6, 8.0), [1H]; 6.93, dd (0.8, 8.0), [1H]; 6.99, dd (1.6, 8.0), [1H]	6, 7	2, 3, 4, 6; 2', 3', 4', 6'; 2'', 3'', 4'', 6''
6, 6', 6''	6.77, t (8.0) [2H]	118.8	1, 2, 3, 4, 5, 7	120.00; 119.89	6.78, t (8.0), [2H]; 6.72, t (8.0), [2H]	5, 7	2, 3, 4, 5, 7	120.04; 120.03	6.72, q (8.0), [2H]; 6.77, t (8.0), [1H]	5, 7	2, 3, 4, 5, 7
7, 7', 7''	7.38, dd (1.6, 8.0), [1H]; 7.36, dd (1.6, 8.0), [1H]	118.4	1, 3, 4, 5, 6	119.71; 119.66	7.37, dd (8.0, 1.6), [1H]; 7.31, dd (8.0, 0.8), [1H]	5, 6	1, 3, 4, 6; 1', 3', 4', 6'	119.81; 119.76; 119.69	7.33, dd (0.8, 8.0), [1H]; 7.30, dd (0.8, 8.0), [1H]; 7.36, dd (0.8, 8.0),	5, 6	1, 3, 4, 6; 1', 3', 4', 6'; 1'', 3'', 4'', 6''

									[1H]		
Ornithine											
8, 8', 8''			172.2; 172.0	173.67; 172.27				173.66; 173.89; 172.23			
9	4.79, m [1H]	51.93	1, 8, 10, 11	54.37	4.82, m, [1H]	10, 11, 12	1, 8, 10, 11	53.95	4.75, m, [1H]	10, 11, 12	1, 8, 10, 11
9'	4.76, m, [1H]	52.53	1', 8', 10', 11'	53.94	4.73, m, [1H]	10', 11', 12'	1', 8', 10', 11'	54.09	4.67, m, [1H]		1', 8', 10', 11'
9''								54.41	4.84, m, [1H]		1'', 8'', 10'', 11''
10, 10', 10''	2.05, m, [2H]; 1.88, m, [2H]	28.8	8, 9, 11, 12	29.89; 29.78	2.07, m, [2H]; 1.92, m, [2H]	9, 11, 12; 9, 11, 12	8, 9, 11, 12; 8, 9, 11, 12	29.99; 29.79; 29.70	2.05, m, [3H]; 1.89, m, [3H]	9, 11, 12	8, 9, 11, 12; 8, 9, 11, 12
11, 11', 11''	1.81, m [4H]	23.5	9, 10, 12	25.03, 24.97	1.82, m, [4H]	9, 10, 12	9, 10, 12	25.09; 25.01; 25.00	1.83, m, [3H]; 1.79, m, [3H]	9, 10, 12	9, 10, 12; 9, 10, 12
12, 12', 12''	2.99, m [4H]	38.9	10, 11	40.30, 40.27	3.00, m, [4H]	9, 10, 11	10, 11	40.31	3.00, m, [6H]	9, 10, 11	10, 11
Serine											
13			169.8	164.44				164.4			
13'			170.7	172.23				169.91			
13''								172.29			
14	4.58, t (4.0, 4.8), [1H]	54.96	8, 13, 15	133.02	4.88, m, [1H]		8', 13', 15'	132.7			
14'	4.80, m, [1H]	51.97	8', 13', 15'	53.12				52.99	4.89, m, [1H]	15'	8', 13', 15'
14''								53.22	4.82, m, [1H]	15''	8'', 13'', 15''

15	3.95, dd (4.8, 12.0), [1H]; 3.82, dd (4.8, 11.2), [1H]	61.23	13, 14; 13, 14	112.57	6.23, s, [1H]; 5.90, s, [1H]	15; 15	13, 14; 13, 14	112.81	6.12, s, [1H]; 5.80, s, [1H]	15; 15	13, 14; 13, 14
15'	4.75, m, [1H]; 4.43, dd (4.8, 11.2), [1H]	64.04	13, 13', 14'; 13, 13', 14'	66.09	4.68, dd (11.2, 6.4), [1H]; 4.53, dd (11.2, 6.4), [1H]	15'; 15'	13, 13', 14'; 13, 13', 14'	65.32	4.56, m, [1H]; 4.53, m, [1H]	15', 14'; 15', 14'	13, 13', 14'; 13, 13', 14'
15''								65.88	4.74, m, [1H]; 4.38, dd (6.4, 11.2), [1H]	15'', 14''; 15'', 14''	13', 13'', 14''; 13', 13'', 14''
